# Elucidating HER2-directed chimeric antigen receptor (CAR) activation mechanism using homology modeling and all-atom molecular dynamics simulation

**DOI:** 10.1016/j.csbj.2025.12.017

**Published:** 2026-01-02

**Authors:** Mariya Hryb, Leah Davis, Stefi Lao, Nicholas J. Paradis, Mary Staehle, Xiaoyang Mou, Chun Wu

**Affiliations:** a Department of Biomedical Engineering, Henry M. Rowan College of Engineering, USA; b Departments of Chemistry & Biochemistry and College of Science and Mathematics, Rowan University, Glassboro, NJ 08028, USA; c Departments of Biological Biomedical Sciences, College of Science and Mathematics, Rowan University, Glassboro, NJ 08028, USA

**Keywords:** Chimeric antigen receptor, Anti-HER CAR, Molecular dynamics simulations, Computational modeling, Computational oncology, Adoptive cell therapy, Immunotherapy

## Abstract

HER2-directed chimeric antigen receptor (CAR) T cells have demonstrated robust *in vivo* cytotoxic activity against HER2-positive tumors. However, the structural basis of CAR activation and signal transduction across the membrane remains poorly defined due to the lack of full-length, high-resolution CAR structures. Here, we used homology modeling and all-atom molecular dynamics simulations totaling 37.7 µs to probe the structural dynamics of a full-length anti-HER2 CAR embedded in an explicit POPC membrane, sampling both apo-form and antigen-bound (holo-form) states across three independent trajectories. The simulations reveal coordinated, antigen-dependent changes in the dynamics of the extracellular antigen-binding domain (AB) and the intracellular signaling domain (SI), coupled through the intrinsic disordered regions including hinge, transmembrane, and costimulatory domains (HI–TM–CS). In the apo state, AB exhibits broad extracellular conformational sampling consistent with antigen search, whereas intracellular SI sampling remains comparatively restricted. Upon antigen engagement, this dynamic coupling is altered in a manner reminiscent of an oscillating coupled pendulum, with reduced AB spatial sampling and increased intracellular SI mobility, consistent with a redistribution of domain dynamics that may facilitate accessibility of signaling motifs and downstream kinase engagement, like LCK phosphorylation. We refer to this antigen-dependent reversal in coupled domain dynamics as a Binding-Induced Domain Dynamics Switch (BIDDS). BIDDS differs from static safety on/off models of T cell receptor activation and is presented as a mechanistic hypothesis to guide future computational and experimental studies of CAR activation and related multi-domain receptor signaling, with potential relevance to other receptor tyrosine kinases including VEGFR, EGFR, and FGFR.

## Graphical abstract

**Figure:**
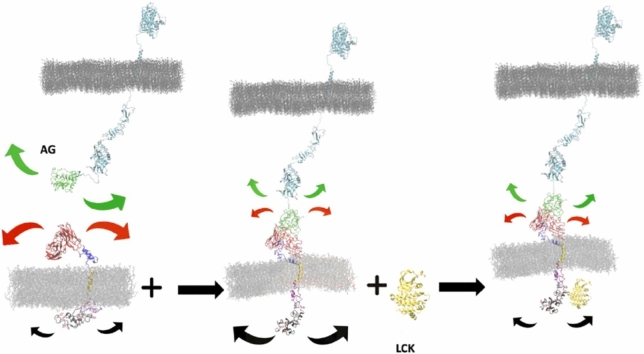
The putative early steps of the HER2-CAR activation that is critical in the immune response of the engineered CAR T-cells. The size of arrow suggests the magnitude of domain oscillation.

## 1 Introduction

Chimeric antigen receptors (CARs) are synthetic receptors that redirect T cells to recognize and eliminate tumor cells, representing one of the most promising strategies in adoptive cell therapy. CAR-T cell therapy has demonstrated remarkable success in treating hematological malignancies (Table S1) and is showing increasing promise for solid tumors. A defining feature of CARs is their modular design, which allows for the rational engineering of receptor components to improve specificity, sensitivity, and therapeutic efficacy. Typically, a CAR is composed of five key structural domains: an antigen-binding (AB) domain, a hinge (HI) domain, a transmembrane (TM) domain, a costimulatory (CS) domain, and a CD3ζ signaling (SI) domain. Each domain is derived from distinct proteins and fulfills specialized roles in mimicking the natural T cell receptor (TCR) signaling cascade. The AB domain, often a single-chain variable fragment (scFv), is responsible for antigen recognition and can be customized based on tumor antigen expression. The HI and TM domains support membrane localization and confer structural flexibility, commonly derived from CD8, IgG or CD28. The intracellular CS and SI domains facilitate robust T cell activation, with CD3ζ mediating signal transduction and the CS domain, commonly 4–1BB or CD28, amplifying the signal [1]. Any of these domains can be interchanged in numerous combinations, drawing from a variety of proteins to tailor signaling, stability, and expression. While the modularity of CARs offers great design flexibility, recent studies underscore the importance of CAR structure in determining therapeutic performance [2]. Despite this, our understanding of the structure-function relationship in CARs remains limited. A deeper structural insight is crucial for optimizing CAR designs to overcome current challenges, including off-target toxicity, limited persistence, and heterogeneous patient response relationship. [3]

Advances in computational biology offer powerful tools to model, predict, and analyze protein structure and function. Techniques such as homology modeling, molecular docking, and molecular dynamics (MD) simulations are widely used to study protein folding, interactions, and conformational changes [4]. However, these methods have traditionally focused on small, soluble, and well-ordered proteins for which high-resolution crystal structures are available. CARs, by contrast, often also include intrinsically disordered domains essential for transmitting signals from extracellular binding to intracellular activation, and their IC, TM, CS, and SI regions lack structural data due to disorder and challenges of membrane protein crystallization [5]. Protein structure predictors such as AlphaFold [6], I-TASSER [7], [8], and RosettaMP [9] offer viable alternatives for modeling full-length receptors when high-resolution solution NMR, X-ray and cryo-EM structure models are either incomplete or missing, but predicted models of disordered regions are often unstable and unreliable [10], [11], [12]. Physics-based, long timescale MD simulations with sufficient conformational sampling can refine these regions by allowing initially inaccurate models to relax and adopt physically realistic conformational ensembles [13], [14], [15]. Despite these capabilities, no MD study has yet characterized the complete structure or signaling mechanism of an anti-HER2 CAR; the only available work used short MD trajectories and examined only extracellular domains modeled from partial or mismatched templates [16]. Thus, comprehensive computational modeling of complete CAR structures, beginning with a full-length initial model and refined through long-timescale MD simulations, represents a critical next step in elucidating CAR activation and guiding rational CAR design.

Human epidermal growth factor receptor 2 (HER2) directed CARs are being considered because of HER2 overexpression in many solid tumors from multiple major systems such as the reproductive, respiratory, digestive, urinary, central nervous and other systems [17], [18]. Recently, anti-HER2 CAR T-cells have been developed and shown promising *in vitro* and *in vivo* anti-tumor activity using HER2-positive cancer cell lines and human xenograft mice models of breast-to-brain metastasis. [19] Interim results from a recent clinical trial, called BrainChild-01 (NCT03500991), demonstrate the promise of anti-HER2 CAR T-cells and the future of CAR T-cell therapy for solid cancers. [20] Although a crystal structure of mouse AB in complex with human HER2 AG is available (PDB ID: 3H3B) (Fig. S1, S5), the high-resolution structures of the other four domains are not available, most likely due to disordered regions that are also critical for their normal function (Table S2). Lack of the full receptor structure hinders our understanding of the receptor activation mechanism that requires long-ranged signal transduction across the membrane, which is a critical step in activating T-cells. This raises a crucial question: What roles do disordered regions play in signal transduction, specifically in influencing receptor conformational changes triggered by protein binding, and is there a general physical model to elucidate this phenomenon?

To date, no MD studies have been performed on an anti-HER2 CAR. One previous MD study analyzed a second-generation CAR consisting of camelid VHH, CD8α spacer/HI-TM, CD28/CS, and CD3ζ/SI. However, the model was constructed by using CD8α crystal structure of a different residue position (PDB ID: 1AKJ) and only the extracellular regions were analyzed during MD simulation. [16] In this study, the initial structural model of the full anti-HER2 CAR was built from a combination of protein crystal structure, NMR solution structure and homology modeling, and was packed in a solvated, physiological POPC lipid membrane, totaling ∼400 K atoms. Then, we performed a 37.7 µs total MD simulation using the world fastest supercomputer, Anton2, to probe and refine the structure and dynamics of this receptor in its apo-form and holo-form. Our MD data shows that the motion of extracellular AG is highly coupled with the motion of intracellular SI, with flexible support (TM-CS), just like a coupled pendulum. Oscillation of two pendulums (extracellular AB and intracellular SI) is coupled by a common support string (HI-TM-CS), and the “oscillation” pattern changes upon mass change due to AB-AG binding. These findings suggest a putative novel mechanism, in which we term the Binding Induced Domain Dynamics Switch (BIDDS). BIDDS could be helpful to explain the working mechanism of a large class of membrane receptor tyrosine kinases which have multiple extra- and intracellular domains that could influence the selectivity and sensitivity of this coupled pendulum model for signal transduction. If so, this insight opens the opportunity to design more selective and sensitive CARs for treating solid cancers and optimizing design.

## 2 Materials and methods

The apo-form of an anti-HER2 CAR consists of five human structural domains: the HER2 single chain fragment variable antigen-binding (AG) domain, the CD8α hinge (HI) and transmembrane (TM) domains, the CD137 (also named as 4–1BB) costimulatory (CS) domain, and the CD3ζ signaling (SI) domain (Table S2), following the architecture of the secondary generation CAR. [21] The critical SI domain is derived from the intracellular portion of the T-cell receptor CD3ζ chain, which plays an important role in coupling antigen recognition to intracellular signal-transduction (Fig. S4, S10). The SI includes six tyrosine residues in three immunoreceptor tyrosine-based activation motifs (ITAMs) (UniProt: P20963) and were suggested by Reth et al. to be involved in intracellular signaling. [22] It is believed that the lymphocyte-specific protein tyrosine kinase (Lck) will bind to ITAMs and phosphorylate the 6 tyrosine residues on the ITAMs upon AG-AB binding, leading to T-cell activation. [23] Lck, a member of Src-family protein kinases, plays a critical role in T lymphocyte activation and development and is required for cell-cycle regulation. [24] Additionally, three polybasic regions (PBRs) were identified and are contained between the ITAMs: PBR 1 (395−399), PBR 2 (406−410), PBR 3 (435−442) (Table S3). [25] Both ITAMs and PBRs were suggested to be conserved (YxxLx_(7−12)_YxxL) or (Yxx(L/I)x6–8Yxx(L/I)) signifying it is functionally important and allowing its interaction with acidic phospholipids. [1], [25]

## 3 Homology modeling

The apo-form of CAR consists of five structure domains: AB, HI, TM, CS, and SI. Conversely, the holo-form of CAR is a complex that additionally includes the HER2 AG domain (Table S2). While the crystal structure of mouse AB in complex with human AG is available (PDB ID: 3H3B), the other domains were built from homology modeling (Table S5-S7).

The crystal structure of the single-chain fragment variable (scFv) anti-HER2 antibody chA21 (chain C) in complex with residues of 1–192 of human HER2 extracellular domain (chain A) (PDB ID: 3H3B; resolution: 2.45 Å) was retrieved from the protein data bank (PDB). [26] The homology model of human anti-HER2 AG domain was built using Maestro’s Prime Homology Modeling program based (Fig. S20) on the crystal structure used in the query sequence (Table S4). Water molecules and non-interacting ions were removed, and missing hydrogen atoms were added to the protein. Maestro’s Protein Preparation Wizard workflow was used to prepare the protein for molecular dynamics (MD) simulations, including optimization of protonation state assignment and energy minimization carried out using the OPLS 2005 force field. [27], [28]

**Fig. 1:**
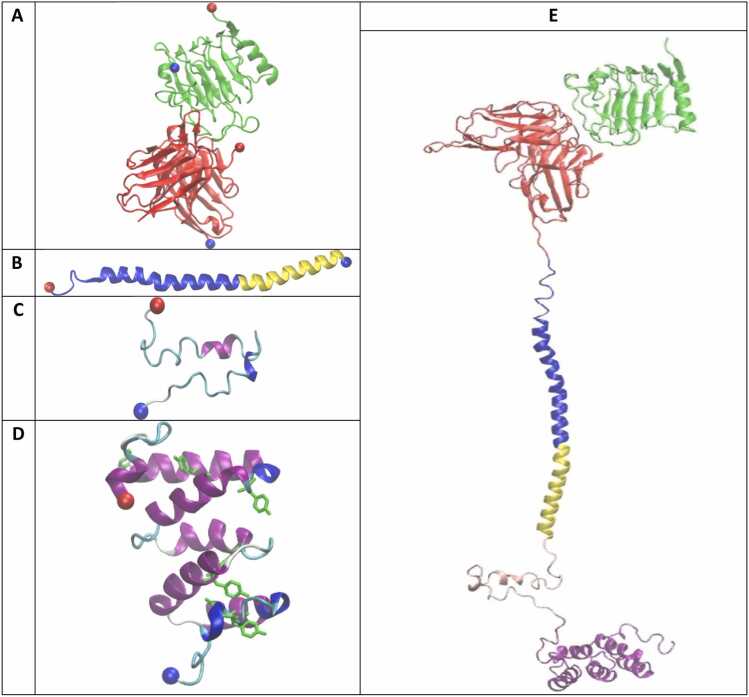
Domains (**A-D**) and full structure of the human anti-HER2 CAR in complex with the human HER2 antigen (**E**). **A:** AB in complex with AG (PDB ID: 3H3B). **B:** HI-TM. **C:** CS. **D:** SI. **E:** anti-HER2 CAR in complex AG. Homology models: AB, HI-TM, CS and SI. AG in green, AB in red, HI in blue, TM in yellow, CS in pink, SI in purple. N-terminal in a red, C-terminal in blue. *Domain abbreviations: AB: Antibody; AG: Antigen; HI: Hinge; TM: Transmembrane; CS: Costimulatory.

The protein sequences of HI (Uniprot ID: P01732), CS (Uniprot ID: Q07011), and SI (Uniprot ID: P20963) in FASTA file format were retrieved from the Uniprot database, and a target sequence was identified for each domain based on their structural topology **(**Table S5-S7**)**. The sequence of the SI domain was modified and had one deletion (ΔQ101). [19] The I-TASSER webserver was used to predict the 3D protein structure for target HI, TM, CS, and SI. [7], [8] The I-TASSER output represents the top 10 threading templates for HI-TM, CS, and SI (Fig. S17-S19) and the top three 3D structure models with global and local accuracy estimations were chosen based on their confidence metrics (C-score:-1.89 - −4.02, TM-score:0.49–0.28, Exp. RMSD 6.0–13.5) (Fig. S21). The first predicted structure model was used for the CS and the SI (Fig. 1**C-D**). Predicted HI structure model included a disordered (coiled) region, and TM was found to be helical. The predicted TM structure was kept for the anti-HER2 CAR structure, and the builder tool was used to build a full helix for the HI in Maestro 10.3. (Fig. 1**B**). The Maestro builder tool was used to connect generated parts to form the complete structure in apo-form and holo-form (Fig. 1**E**). The complete structures were also preprocessed, optimized and minimized in Protein Preparation Wizard. [28]

## 4 MD simulation

The anti-HER CAR structure was subjected to MD simulations in both apo-form and holo-form. Detailed methodology of the simulation setup, protocols and post-simulation analysis can be found in the supporting document. In brief, the Desmond simulation package was employed for the initial relaxation and a 10 ns production run, while the remaining production run was conducted using Anton 2. [22] Each system underwent a total of three simulation runs to ensure sufficient sampling of conformational ensembles and to reduce potential bias in individual runs and sample any slow conformational changes in the protein-membrane system (Table S8). The properties obtained from the simulation encompassed root mean square deviation (RMSD), root mean square fluctuation (RMSF), end-to-end distance, radius of gyration (Rgyr), ligand-protein contacts, and protein secondary structure elements (SSE). Post-MD simulation analysis involved calculating Cα protein and ligand RMSDs over the trajectories to assess sufficient sampling of conformational ensembles, followed by the utilization of the Simulation Interactions Diagram (SID) tool and VMD. [29] In addition, trajectory clustering analysis was used to group complex structures from the first MD trajectory of each system. [30] The main text presents the results obtained from the combined analysis of all three trajectories, while the results from each individual trajectory can be found in the supporting documents.

## 5 Results

### 5.1 Structure model of the apo-form and holo-form of the human anti-HER2 CAR

The structure model of the apo-form and holo-form of the human anti-HER CAR were built as described in the method section. In brief, a 57 % sequence identity between the human AB sequence and mouse AB sequences supports our approach of using homology modeling to create a human AB structure model based on the mouse AB structure. [31] The crystal structure of the mouse AB in complex with human AG (PDB ID: 3H3B; resolution: 2.45 Å) was used as the template to build the homology model of human AB in-complex with human AG (Fig. 1**A**). [26] The crystal structure lacked a linker portion (residues 109–139), which was subsequently filled in using the Maestro program of Schrödinger Software Package. The remaining section of the human AB displayed strong alignment with the mouse AB crystal structure (PDB ID: 3H3B) (Fig. S20), demonstrating the high quality of the homology model. High-resolution structures of HI-TM, CS and SI were not available and three homology models for each of these domains were built using the I-TASSER webserver (Fig. S17-S19). The first structure model of CS and SI was selected for building the anti-HER2 CAR receptor (Fig. 1C-D, S20)**.** The homology models of HI-TM buried the extracellular HI in the membrane by including a tightly packed, disordered coiled hinge, between extracellular HI and the helical TM (Fig. S21). This does not mimic the *in vivo* conformation, as it is understood that the hinge enhances the flexibility of antibody-binding. To fix this, the builder tool in Maestro 10.3 software was used to build a full helix for the HI domain, and the helical part of TM was kept for the anti-HER2 CAR receptor. [21], [22] Finally, the apo-form and the holo-form protein structures were prepared by using the Maestro Protein Preparation Wizard workflow described in the Methods section (Fig. 1**E**).

### 5.2 Assessing sampling of conformational ensembles from MD simulation

MD simulations were then used to relax and explore the structural dynamics of the HER2-CART systems over time. The stability of the anti-HER CAR complete structure in apo-form and holo-form was examined by 5.4 µs MD simulations averaged over three combined trajectories (Fig. S23A**-B)**. The Cα-RMSD values of receptor structure in apo-form and holo-form were calculated (Table S9) to check sufficient sampling of conformational ensembles of the MD simulations as defined in the methods section. To enhance the robustness of these observations, three independent trajectories were performed for each system, yielding a combined simulation time of 37.7 μs. Using multiple trajectories helps reduce potential bias in any single trajectory and allows sampling of slow conformational changes in the protein–membrane system. Relatively flat plots were observed after 4 µs for the apo-form and after 5 µs for the holo-form, and after an additional 5 µs of simulation in the first trajectory of the holo-form. This suggests that both apo-form and holo-form systems had amply sampled the representative conformational ensembles for the remainder of the simulations. We denote that due to the large, heterogeneous, multi-domain nature of the simulation system, additional simulation time is likely needed to observe its convergence towards the equilibrium state.

### 5.3 RMSD and Rgyr results reveal a significant conformational difference between the apo-form and holo-form of the anti-HER2 CAR

Average Cα-RMSD values over three trajectories were calculated for the apo-form (Fig. S23A) and holo-form (Fig. S23B) system. To decipher the conformational changes of the holo-form complex, Cα-RMSD values of the complex, receptor and ligand were calculated after fitting the complex and shown in Fig. S23B. The Cα-RMSD of the AG-AB complex was also calculated to assess the stability of AG-AB binding, AG, and the AB domain when aligned to the AG-AB complex, resulting in Cα-RMSD values of 5.8 ± 0.3, 5.7 ± 0.5, and 6.1 ± 0.3 Å, respectively. In addition, intra-domain and inter-domain Cα-RMSD changes were measured. Intradomain changes when aligned to their respective domains for AG and AB, and yielded Cα-RMSD values of 3.4 ± 0.1 Å and 4.2 ± 0.3 Å, as shown in Fig. S23C and Table S10. Interdomain change of AG and AB was calculated to be insignificant: the Cα-RMSD values were 2.3 Å for AG and 1.9 Å for AB, suggesting intra-domain change mostly contributes to the overall change in Cα-RMSD of the AG-AB complex (Table S10).

**Fig. 2:**
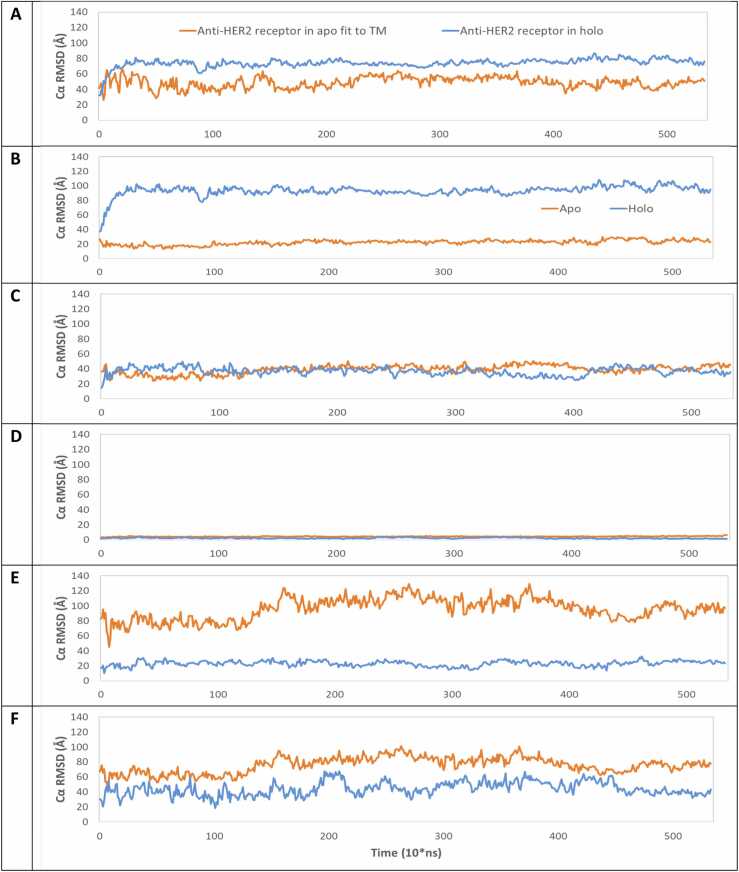
The development of the Cα-RMSD of the receptor parts in apo-form (orange) and holo-form (blue) by all fitting to TM of the CAR for the combined trajectories. **A:** CAR. **B:** AB. **C:** HI **D:** TM **E**: CS. **F**: SI.

When fitting to the TM (Fig. 2**A**), the receptor Cα-RMSD difference (∼25 Å) between the holo-form system and the apo-form system suggests that the binding to AG induces a large overall conformation change of the receptor (Table S9). Furthermore, the Cα-RMSD of each domain aligned to the TM (overall difference) and itself (intra-domain difference) was calculated by subtracting the intra-domain Cα-RMSD difference from the overall Cα-RMSD difference between both systems to estimate the contribution of inter-domain difference (Table S9). The inter-domain differences in Cα-RMSD values were as follows: 71.0 Å for AB, −2.0 Å for HI, 72.6 Å for CS, and 29.7 Å for SI. These measurements lead to four notable observations. First, concerning Cα-RMSD differences, the overall conformational difference in AB (71.5 Å) primarily stems from the inter-domain portion (71.0 Å), rather than the intra-domain component (0.5 Å). Second, the difference in HI is negligible. Third, the difference in CS (75.6 Å) results from inter-domain changes (72.6 Å) rather than intra-domain alterations (1.9 Å). Fourth, the difference in SI (32.3 Å) is predominantly attributed to inter-domain modifications (29.7 Å), with a minor contribution from the intra-domain segment (2.5 Å).

The Cα-RMSD of the CAR receptor and its domains in the apo-form and holo-form systems after fitting to TM only were compared to identify the detailed conformational difference of its domains due to AG binding (Fig. 2 **and** Table S9). The receptor Cα-RMSD of the holo-form (74.6 ± 4.0 Å) was significantly greater than the Cα-RMSD of the apo-form (48.7 ± 6.7 Å), however each domain exhibits different trends, as is seen with AB (apo-form: 22.7 ± 3.2 Å; holo-form: 92.4 ± 4.8 Å), HI (apo-form: 39.7 ± 5.3 Å; holo-form: 36.6 ± 4.9 Å), TM (apo-form: 4.5 ± 0.4 Å; holo-form: 2.3 ± 0.6 Å), CS (apo-form: 97.7 ± 13.5 Å; holo-form: 23.2 ± 3.4 Å) and SI (apo-form: 77.0 ± 10.1 Å; holo-form: 44.7 ± 9.2 Å) (Table S9). Clearly, AB, CS, and SI contribute the most to the overall receptor conformational difference between the apo-form and holo-form. The relative contributions of intra-domain differences (domain aligned to itself) and inter-domain differences (intra-domain difference - overall Cα-RMSD difference**) (**Table S9) between the apo-form and holo-form systems was also calculated (Fig. S27-S30). One note is that since both overall and intra-domain change was calculated by fitting to TM, the inter-domain difference for TM is 0 Å. The inter-domain differences for AB (71.0 Å), HI (-2.0 Å), CS (72.6 Å) and SI (29.7 Å) suggest that 1). The overall conformation difference between the two systems for AB (71.5 Å) is from the inter-domain part (71.0 Å); 2). the difference for HI is insignificant; 3). The difference for CS (75.6 Å) is from the inter-domain part (72.6 Å), 4). The difference for SI (32.3 Å) is mainly from the inter-domain part (29.7 Å) **(**Table S9).

Rgyr demonstrated lower values for N-terminal, and higher values for C-terminal CAR portions in the holo-form compared to apo-form. To characterize the size change of the receptor, Rgyr values of the CAR structure in apo-form and holo-form were calculated for all three combined trajectories and shown in Table S12 and Fig. S45. Overall, compared to the apo-form, the holo-form demonstrates smaller Rgyr values with a decrease by 2.6 Å for the receptor. However, Rgyr of N-terminus becomes smaller in holo-form confirmed by 1.0 Å decrease, while Rgyr of C-terminus increases by 1.1 Å in response to the binding of antigen.

AB-to-TM distance decreased while TM-to-SI distance increased in holo-form. Last snapshot structures of the anti-HER2 CAR holo-form and apo-form is shown in three view perspectives (Fig. S43). In the holo-form, AB in red exhibits a bending toward TM. Furthermore, both CS and SI appear to have a less extended conformation in holo-form compared to apo-form, thereby shortening the distance between TM and SI (Fig. S43). The distance between various CAR parts in the anti-HER2 CAR in apo-form and holo-form was measured for the combined three trajectories (Fig. S44) and their values following sufficient sampling of the conformational ensembles were calculated (Table S11). AB to TM distance was greater in the apo-form (54.8 ± 3.2 Å) than in the holo-form (49.9 ± 2.9 Å), with a difference of 4.9 Å (Table S11). However, the opposite was observed between SI to TM, as the distance was greater in the holo-form (56.1 ± 6.8 Å) compared to apo-form (43.7 ± 2.7 Å), with a difference of 12.4 Å (Table S11).

### 5.4 The holo-form displays lower RMSF values for AB and higher RMSF for CS and SI than the apo-form

**Fig. 3:**
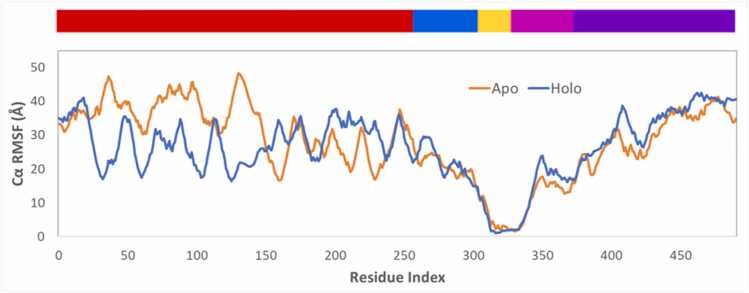
The average of Cα Root Mean Square Fluctuation (RMSF) profiles of the apo-form in orange and holo-form in blue for the combined trajectories. Five structure domains are color coded starting from N-terminal region: AB in red, HI in blue, TM in yellow, CS in pink, SI in purple. Individual trajectories are presented in Fig. S46.

RMSF is a measure of local atomic flexibility and is used to assess structural flexibility during MD simulation. The average Cα-RMSF profiles of the apo-form and holo-form fit to TM are shown in Fig. 3 and show that compared to the apo-form, the holo-form complex displays lower flexibility for AB (-5.2 Å) and higher for intracellular CS (+2.7 Å) and SI (+3.3 Å) (Table S14). This is consistent with the BIDDS model that the binding of AG with the AB of the anti-HER2 CAR in the holo-form reduces its flexibility, but increases the flexibility of the CS and SI of the CAR in the holo-form.

### 5.5 Clustering analysis

Clustering analysis reveals significant conformation differences between holo-form and apo-form. Clustering analysis for the first trajectory based on anti-HER2 Cα-RMSD between conformations grouped the holo-form into 38 clusters and the apo-form into 33 clusters, both with 2.5 Å RMSD as the cutoff distance. Representative structures of the most abundant clusters, whose population is ≥ 2 % of total population, are shown in Fig. S48**-49**. Two key differences were observed for HI. First, the hinge region is less extended in the holo-form, causing a smaller distance between AB and the membrane and a less extended conformation for the intracellular part (Fig. S48**-49**). Second, a smaller distance between the C-terminus and membrane in the holo-form is due to its more compressed conformation compared to the apo-form (Fig. S48**-49**). The difference in TM is small between the two systems. Overall, the difference between snapshot structures of holo-form and apo-form provided by the clustering analysis (Fig. S48**-49**) show similarity to the last snapshot structures of MD simulation for holo-form and apo-form in Fig. S43 as well as the end-to-end distance and Rgyr results in Table S11**-12**.

### 5.6 AG binding reduces the flexibility and the spatial sampling of AB but increases the flexibility and the spatial sampling of SI

**Fig. 4:**
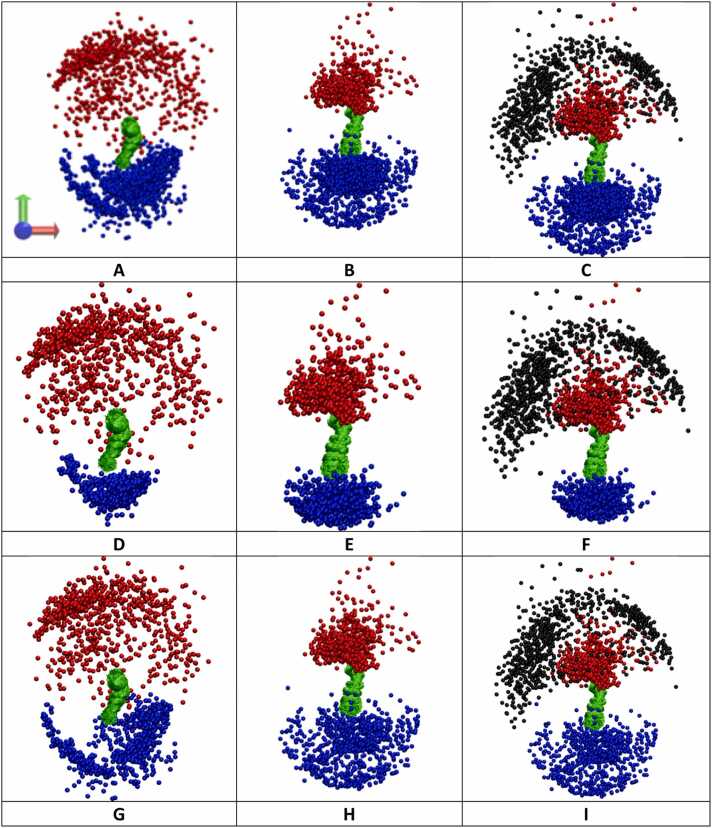
The center distribution change of the key domains of the receptor upon binding to AG for all three combined trajectory. Key domains presented from a top viewpoint (see Fig. S31**-32** for the other two viewpoints): AB (red); CS and SI (blue), and AG (black) with aligned TM (green). The side viewpoint is indicated by the three axes (A), in which Z (green) is the transmembrane direction; and X (red) and Y(blue) are the membrane plane (see bottom left-hand corner in Fig. 4**A)**. Domain distribution of CS and SI combined in apo-form (**A**) and holo-form without AG (**B**) for clarity and with AG (**C**), CS only in apo-form (**D**) and holo-form without AG (**E**) for clarity and with AG (**F**), and SI only in apo-form (**G**) and holo-form without AG (**H**) for clarity and with AG (**I**).

**Fig. 5:**
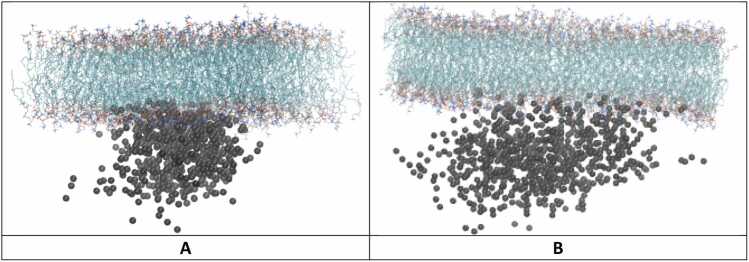
The center distribution of the positively charged amino group of key SI polybasic residues in the combined trajectory of apo-form (**A**) and holo-form (**B**) system. Nitrogen of the amino group of arginine and lysine of PBR 1 (residue 395–399) and PBR 2 (residue 406–410) in black.

To visualize the inter-domain difference between the apo-form and the holo-form system, the domain spatial distribution of the key domains were analyzed based on three different viewpoints, the side, top and bottom perspective, as shown in **Fig. 5, S31-32**, respectively. Fig. 4**A** includes three axes: X-axis in red, Y-axis in blue, and Z-axis in green. While the X- and Y-axes lie in the plane of the membrane, the Z-axis is perpendicular to the membrane (Fig. 4). The plane of the side viewpoint is composed of the Z- and X-axes and presents the spatial distribution in the lateral direction forming an arch shape for both the extracellular and intracellular domains due to overall three-dimensional spherical shape (Fig. 4). The top and bottom viewpoints both lie along the X- and Y-axes and show a circular shape of the overall three-dimensional spherical shape of the center domain distribution (Fig. S**31–32**). Each viewpoint presents the domain center distribution for the apo-form and holo-form system with and without the AG for clarity, and considers the intracellular domain as CS only, SI only, and CS and SI combined, for both apo-form and holo-form systems. Interestingly, while the apo-form demonstrates a greater distribution of the AB in the extracellular space and very narrow conformational sampling in the intracellular space for CS-SI (Fig. 4**A**), the holo-form demonstrates the opposite trend (Fig. 4**B-C**). Specifically, the spatial distribution of the extracellular AB is more flexible in the apo-form, forming an arch in the lateral direction (Fig. 4**A**) and a more spread-out circular shape from the top and bottom perspective (Fig. S31A, S32A). However, the conformational sampling is narrower in the lateral direction in holo-form, including a restricted circular distribution shown in the top view (Fig. S31B**-C**). To probe the individual response of intracellular CS and SI, the center distribution of CS and SI were analyzed separately. The CS demonstrated a less significant increase in comparison to CS and SI combined as well as SI only for the side, (Fig. 4**D-F**) top (Fig. S31D**-F**) and bottom perspectives (Fig. S32D**-F**). SI demonstrated a more significant increase from its restricted conformation in the apo-form (Fig. 4**G**) to a very flexible arch in the lateral direction in the holo-form with (Fig. 4**H**) and without AG (Fig. 4**I**). Additionally, the bottom perspective demonstrates its greater distribution in the holo-form with (Fig. S32I) and without the AG (Fig. S33H) in comparison to the small circular shape of the apo-form (Fig. S32G), thereby contributing to a larger spherical shape when considering its overall shape based on the three axes. These findings suggest the signal transduction mechanism in our BIDDS model, which will be presented in more detail in the discussion section.

### 5.7 Safety-on/off model suggestion of ITAM tyrosine sequestration in lipid bilayer not observed in apo-form despite increased bilayer contacts

**Fig. 6:**
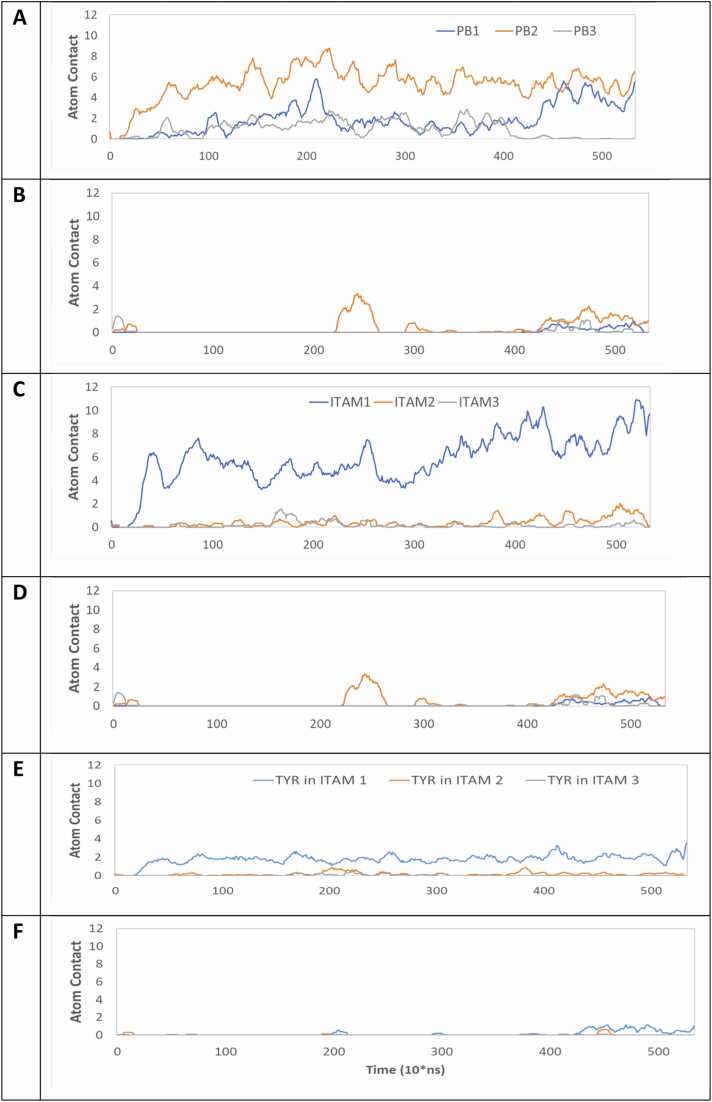
The atom contacts between the three PBRs, ITAMs and tyrosine residues (TYR) of ITAM with POPC membrane for apo-form and holo-form for all three combined trajectories. **A**: PBRs in apo-form; **B**: PBRs in holo-form; **C**: ITAMs in apo-form; **D**: ITAMs in holo-form; **E**: TYR in apo-form; **F**: TYR in holo-form.

To assess the interaction between CD3ζ and the membrane as suggested by the safety on/off model ^32^, membrane contacts between PBR, ITAM, TYR residues in ITAMs and the POPC membrane were visualized and calculated with a distance cutoff of 2.5 Å (Table S13). For atom contact between PBRs and the membrane, visualized by plotting the position of the positively charged amino group of key SI polybasic residues for the full trajectory of the two systems (Fig. 5)**,** the atom contacts for two systems are shown (Fig. 6**A-B).** With the average of nine contacts for the apo-form and only one contact for the holo-form, PBR1 and 2 have more interaction with membrane in the apo-form than in the holo-form (Table S13). For ITAMs and the membrane (Fig. 6**C-D**), there are seven average contacts in total in the apo-form compared to only one contact in the holo-form (Table S13). For the contacts between the tyrosine residues (TYR) and the membrane (Fig. 6**E-F**), although the apo-form demonstrated an increase by 2 atom contacts compared to the holo-form (Table S13), deep insertion of the key TYR residues into the hydrophobic core of the lipid bilayer as suggested by the safety on/off model was not observed. Because the membrane model included only the neutral lipid POPC, potential contributions from acidic phospholipids cannot be excluded and may influence the system’s behavior.

### 5.8 Secondary Structure Element (SSE) analysis suggests while AB and TM maintain order, HI becomes more disordered, and CS and SI significantly decrease in turn conformation

**Fig. 7:**
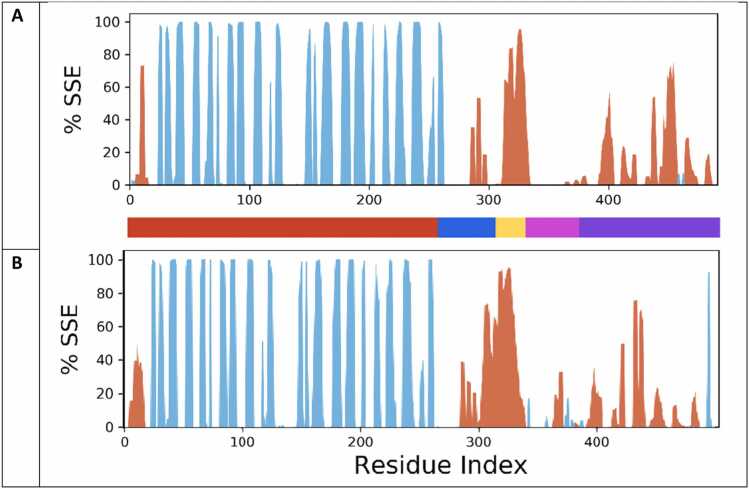
Secondary structure elements (SSE) of the apo-form and holo-forms of the receptor for all three combined trajectories**. A-B:** SSE distribution by residue index throughout the protein structure. **A:** apo-form. **B:** holo-form. The secondary structure is coded by color: α-helix in orange, β-sheet in blue, coil in white. Five structure domains are color coded starting from N-terminal region: AB in red, HI in blue, TM in yellow, CS in pink, SI in purple. Individual trajectories are presented in Fig. S47.

Protein SSE analysis for the two systems throughout the simulations was performed using Maestro’s Simulation Interaction Diagram tool to investigate differences in the secondary structure of the anti-HER2 CAR in apo-form and holo-form (Fig. 7). MD simulation of the apo-form demonstrated that the AB and TM were more ordered, but the HI, CS and SI were more disordered. During MD simulation, no significant difference in SSE was found for AB between apo-form and holo-form, maintaining its turn and β-extended conformation. More detailed SSE results for these domains (Table S15**-16)** are described in the supporting document. Moreover, the crystal structure of Lck was aligned with a tyrosine-substrate complex (PDB ID: 4IAC) and demonstrated that Lck exhibits a secondary structure similar to the complex of protein kinase A in complex with its substrate. This suggests a similar mechanism of phosphorylation of SI by Lck that would require SI to adopt an extended, coiled conformation (Fig. S52).

MD simulations reveal AB and TM were structurally ordered, HI became more disordered, and CS and SI significantly decrease in turn conformation and converted into α-helix and coil. To investigate differences in anti-HER2 CAR protein secondary structure, SSE analysis was performed for both apo-form and holo-form simulation systems for the combined (Fig. 7) and individual trajectories **(**Fig. S47). SSE values for the anti-HER2 CAR receptor and each domain were compared between the homology model (HM, initial structure) and the MD apo-form structure (Table S15). SSE analysis was also performed between the MD-apo-form and MD-holo-form structures (Table S16). Overall, AB did not show significant change, maintained its initial β-sheet rich conformation and remained ordered; HI and SI exhibited conversion of their helical content into coil and β-extended confirmation and became disordered; TM showed conversion of random coil into α-helix and became more ordered (Table S15). No significant difference in SSE was observed for AB between apo-form and holo-form (Table S16).

### 5.9 Dynamic network analysis reveals changes in dynamic behavior of the anti-HER2 CAR in complex with AG

A Dynamical network model was used to identify protein structural units exhibiting correlated movements (referred to as communities), pathways for allosteric activation, as well as critical residues involved in these interactions. [33] A comparison of the models between holo-form and apo-form of anti-HER2 CAR system is presented in Figure S52. 25 communities in the holo-form system and 23 communities in the apo-form system are depicted in different colors and additionally represented as domains per community for both systems in Figure S52. Considering communities grouped into the anti-HER2 CAR domains and HER2 AG, several domains demonstrate significant change in community number including a decrease by 6 communities for AB and by 2 for HI (Figure S52). However, there is an increase by 1 community for CS and by 3 for SI, and no change in community number for TM in the holo-form compared to the apo-form (Figure S52). Overall, a change in a community number indicates a change in dynamic behavior, primarily including a decrease in stimuli region and an increase in response region in the holo-form complex compared to the apo-form.

## 6 Discussion

An anti-HER2 CAR consists of an extracellular AB and HI domain, a TM domain and an intracellular CS and SI domain. When the anti-HER2 CAR binds to HER2, it triggers T-cell activation, leading to cytotoxicity and the elimination of the target cell. One critical component in this process is the SI contains three ITAMs with three PBRs between the ITAMs. Upon activation, the Src family kinases, especially Lck, become active and phosphorylate the ITAMs. The phosphorylated ITAMs serve as docking sites for ZAP-70 and are essential to T-cell activation. Xu and colleagues have suggested a Safety On/Off Model for CD3ζ activation of the T-cell receptor (TCR) in which the key tyrosines in ITAMs are buried in the membrane through putative ionic interactions between acidic phosphate lipids and PBRs. [25], [34] [32] This locking prevents the ITAMs from getting activated. When the TCR is engaged, CD3ζ gets "unlocked," making ITAMs available for activation by Lck. However, this model is inconsistent with *in vivo* cellular mutagenesis studies by Zhang et al. [35] and Deford-Watts et al. [1] In summary, the authors found that mutating the basic residues (arginine/R and lysine/K) to alanine/A in the PBR and thereby removing the ionic interactions that lock tyrosine’s in the membrane actually did not increase the baseline phosphorylation of ITAMs and attenuated downstream responses induced by TCR engagement. Therefore, the precise activation mechanism of TCR remains elusive. [35]

In this study, we built a homology model of a HER2-directed CAR and utilized all-atom MD simulations with an explicit POPC membrane to probe the structure and dynamics of this receptor in its apo-form and holo-form at the molecular level to decipher its activation mechanism. Before interpreting the mechanistic implications of our MD simulations, it is essential to first evaluate whether the long-timescale trajectories yielded structurally credible conformational ensembles. Although detailed experimental structural constraints for HI, CS and CD3ζ SI domains are not yet available, their global structural features (specifically, their intrinsic disorder regions) are well established from both biochemical studies and from our PONDR disorder predictions (Fig. S6-S9). Importantly, we denoted the inherent inaccuracy of initial homology models of IDRs; the initial homology models produced by I-TASSER artificially imposed more order than is biologically expected, particularly in the HI domain, where a continuous α-helix was used to avoid membrane burial artifacts in the raw I-TASSER output structure. During the relaxation step of our MD simulations, however, these artificially ordered regions spontaneously lost their α-helical secondary structure and transitioned into coil and β-extended secondary structure conformations (Table S15–S16; Fig. S47) consistent with the flexible, non-helical character of the CD8α hinge observed experimentally. Similarly, a disordering was observed for the intracellular SI domain, which is known to function as a highly dynamic signaling tail; MD simulation trajectories reproduced this expectation by reducing its initial α-helical content and sampling a predominantly disordered ensemble. By contrast, ordered domains bearing strong experimental support (the β-sheet rich AB domain and the helical TM region) remained structurally stable throughout the MD simulations. These observations demonstrate that the MD simulations did not merely propagate template artifacts but instead refined the receptor toward experimentally consistent disordered/ordered patterns across their respective domains. Establishing this agreement provides an essential foundation for interpreting the subsequent mechanistic differences observed between the apo-form and holo-form states and for evaluating the BIDDS model for the HER2 CAR system.

Although the tyrosines in ITAMs and PBRs have more atom contacts with the membrane in the apo-form than that in the holo-form, we found that the tyrosines in ITAMs are not buried in the membrane and the ITAMs and PBRs are not statically associated with membrane. In fact, we observed very dynamic behavior in the extracellular AB and intracellular CS-SI domains in both apo-form and holo-form. AB domain sampled larger extracellular space in the apo-form (Fig. 4**A-C**) likely allowing it to search for the AG domain to bind. The responding CS-SI domain was restricted when sampling intracellular space, likely allowing them to become hidden from their protein partners for signal transduction. In the holo-form, AB is restricted to sample smaller extracellular space, CS-SI is activated to sample larger intracellular space, likely allowing them to search for their protein partners to bind (Fig. 4**D-I**).

**Fig. 8:**
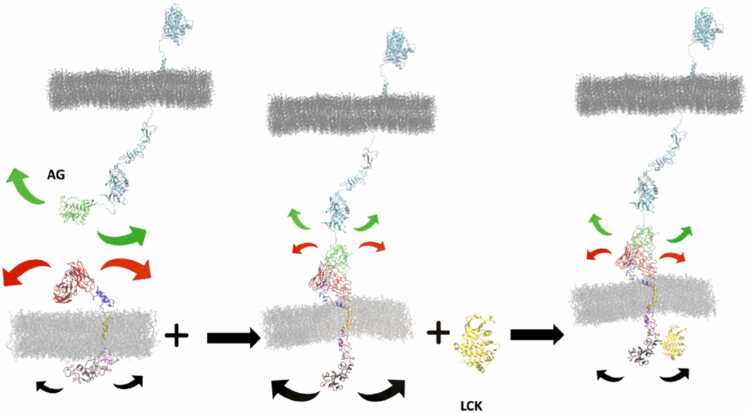
The putative early steps of the HER2-CAR activation that is critical in the immune response of the engineered CAR T-cells. The size of arrow suggests the magnitude of domain oscillation.

The foundation of our proposed BIDDS model is rooted in the dynamic behavior of the stimulating and response domains of the receptor, which bears resemblance to a coupled pendulum system. In this analogy, the oscillation of two pendulums, represented by the extracellular stimulating AB domain and the intracellular responding SI domain, is interconnected by a common supporting string (HI-TM-CS). Notably, the oscillation pattern undergoes alterations when there is a shift in mass due to the binding between AB and AG, as illustrated in Fig. 8. BIDDS might be further applied to kinase receptors such as VEGFR, EGFR, PDGFR, TrkA and FGFR (Fig. S55C**-H**) as they share a common disordered region to both ends of ordered TM. (Fig. S55I**-G**) This insight presents a great opportunity to design a more selective and sensitive CAR for treating solid cancers (Fig. 8). Accordingly, BIDDS is presented as a mechanistic hypothesis to guide future experimental and computational studies of CARs and related receptor tyrosine kinases, while acknowledging that quantitative validation remains to be established.

We emphasize the value of probing the detailed dynamics of disordered protein regions in CAR and its domains, which play a crucial role in its signaling mechanism, using MD simulations. In BIDDS, the disordered regions are attributed to HI and CS flexibility compared to ordered regions of TM, with its flexibility being modified upon AG binding. For the extracellular region, HI is a flexible linker in the apo-form and allows a greater special sampling of stable AB facilitating molecular recognition of AG for AB. Upon AG binding, flexibility of the linker is modified and becomes reduced for HI, therefore, AB is restricted in space sampling. In BIDDS, it is also true for the intracellular part, where binding modifies flexibility with an increase for holo-form. CS (with a short disordered cytoplasmic TM region) functions as a flexible linker, and, upon AG binding, allows SI to sample more space in the holo-form enabling SI engagement with Lck to trigger an intracellular phosphorylation cascade for SI. [36] Indeed, protein disordered regions lack a fixed 3D structure and were shown to contribute to 30 % of eukaryotic proteins. [26] Further, the majority of cancer-associated proteins and more than 70 % of signaling proteins were found to have long disordered regions. [37] Intrinsically disordered regions (IDRs) or intrinsically disordered proteins (IDPs) have been attributed to various vital cellular roles including signal transduction, transcription, and post-translational modifications. [38], [39], [40], [41] Overall, for many proteins, disordered regions and IDRs, such as the CD8 hinge and the CD3ζ cytoplasmic tail, play a critical role in their function. For example, for native CD3ζ, its polymerization, phosphorylation, and protein-protein binding function arises from its disordered state. [36] This intrinsic flexibility makes it notoriously challenging to determine the detailed structure of IDPs using conventional ensemble-averaging experimental methods. Computational modeling tools can help bridge this gap. However, homology modeling provides only approximate initial structures for IDRs and should not be interpreted as defining their functional roles. MD simulations can directly probe the structural ensemble of IDPs at atomistic resolution, and thus decipher the structural basis for the biological functions of IDPs by allowing the initial IDR starting conformations to relax into their disordered ensembles with sufficient simulation time (see Fig. S47)**.** Nevertheless, because IDR ensembles are sensitive to model assumptions, force fields, and sampling limitations, the BIDDS picture emerging from our simulations should be regarded as a qualitative mechanistic hypothesis rather than a fully validated model.

The deep penetration of key TYR residues into the lipid bilayer's hydrophobic core, as proposed by the safety on/off model, was not observed in our simulations. However, we observed that in the apo-form, PBR, ITAM, and the critical TYR residues within ITAMs demonstrate increased atomic interactions with the membrane compared to the holo-form system. This suggests a dynamic association with the membrane, rather than a static insertion, which acts to inhibit phosphorylation. This phosphorylation inhibition occurs probably due to the reduced affinity of Lck for CD3ζ in the apo-form, thereby impacting the phosphorylation process. Furthermore, mutating arginine and lysine residues in PBRs to alanine, while not affecting the disordered regions in CD3ζ as predicted by PONDR (as seen in Figs. S56 and S10), suggests that such mutations do not significantly alter dynamic association with membrane would not be affected and thus the binding affinity with Lck would not be increased to promote the tyrosine phosphorylation domain flexibility, but the mutations might impair BIDDS, thus reducing signal transduction. Therefore, our BIDDS could explain the facts that there is no baseline change in the phosphorylation and attenuate the downstream responses of TCR activation in the mutagenesis experiments. [1], [35] Because our simulations use a simplified POPC membrane that does not represent the anionic lipid environment of T cell membranes, we are unable to definitively assess Safety On/Off–type membrane-burial mechanisms. Incorporating anionic lipid components in future studies will be essential for further evaluating the proposed BIDDS framework.

Our quantitative analysis using RMSD, RMSF, inter-domain distances and Rgyr supports our dynamic interpretation. RMSD results of the difference between apo-form and holo-form suggest the intra-domain change is insignificant for AB (0.5 Å) which is consistent with no change in its secondary structure. Instead, the overall change (71.5 Å) comes from inter-domain change (Table S9), which is supported by a great decrease in RMSF values in holo-form as compared to apo-form (Table S10). The RMSD and RMSF difference between the apo-form and holo-form is less significant for HI and TM, further confirming its function of a common support string in the coupled pendulum model. Similarly, AB and both CS and SI demonstrated a great contribution of inter-domain difference, 72.6 Å and 15.8 Å, respectively, to the overall difference between the two apo-form and holo-form, 74.6 Å and 20.2 Å (Table S9). Consistent with RMSD data and distribution analysis, RMSF for CS and SI were higher in holo-form compared to apo-form (Table S14). CS and SI adopt a more disordered conformation with an increase in coil conformation allowing the intracellular part to become more solvent-exposed (Table S16). For the holo-form, the distance from TM to AB decreases by 4.9 Å, and the distance from TM to SI increases by 12.4 Å (Table S11). Similarly, Rgyr shows a small decrease for the N-terminal extracellular domains and a small increase for the C-terminal intracellular domains in holo-form compared to apo-form (Table S12). Dynamic network analysis also suggests the changes in community number between the apo-form and holo-form structures is a result of the change in the decreased stimulus and increased response region within the holo-form complex upon AG-to-AB binding compared to the apo-form structure, which could contribute to the change in flexibility of the holo-form structure compared to the apo-form structure.

Since this computational study just includes the AG domain of the HER2 kinase receptor, it raises the question of whether the observed BIDDS model for AG-CAR could be true if the entire system of HER2 is included to form a HER2-CAR complex, as depicted in Fig. 8**.** Encouragingly, the key structural motifs that support BIDDS like the flexible linkers, are also observed in the full HER2 structure (linker 1–3 in Fig. S5). In addition, upon AG-AB binding between the full HER2 and CAR, a larger intercellular complex HER2-CAR than AG-CAR would be formed to restrict the flexible motion of AB in CAR much more than the small AG alone. In other words, before the binding, AG and AB are both flexible facilitating the process of molecular recognition and interaction between CAR and HER2 as shown in big arrows in Fig. 8. Upon binding, due to the restraints of the full HER2 receptor that is embedded in membrane, the flexibility of AG-AB could drop greatly, thus likely inducing larger dynamic change of the intracellular part of CAR; the BIDDS effect of the AG-AB complex (holo-form) could be more pronounced than in its apo-form structure. Future experimental studies are required to validate our model and our interpretation that AG binding reduces AB flexibility, increases SI flexibility and enhances accessibility for access to Lck binding.

While this study provides an atomistic description of a single anti-HER2 CAR construct complexed with the HER2 AG domain, we denote several limitations of this study that do not explicitly represent important biological features of this construct. First, we utilize a pure POPC membrane not including acidic phospholipids (e.g. PS or PIP2), so our simulations may under-represent specific electrostatic interactions between polybasic regions and anionic lipids. Second, we only model extracellular AG of HER2 without HER2 dimerization, co-receptors, or other components of the immunological synapse for the sake of simplifying the construct’s design, which may not capture the full extent of the dynamic nature of this construct. Third, the CAR construct was not glycosylated and external mechanical forces or crowding effects are not included or considered for construct simplicity, which may miss other molecular phenomena not observed in our MD simulations. Finally, although the total simulation time is long for all-atom MD, the accessible timescales still limit exhaustive sampling of the enormous conformational space of this system. Considering these limitations, BIDDS should therefore be viewed as a mechanistic hypothesis consistent with the above simulated systems, which extensive experiments are needed to fully validate BIDDS as a model for *in vivo* CAR-T or TCR activation.

Classical models of protein–protein communication emphasize binding-induced structural changes to drive this communication, in which ligand engagement triggers a well-defined conformational rearrangement that propagates a signal. However, extensive biophysical evidence shows that this paradigm does not generalize to systems whose key functional regions are IDRs. Intrinsically disordered proteins (IDPs) and IDRs typically resist stable disorder-to-order transitions, even upon ligand binding, and instead form “fuzzy” or dynamically heterogeneous complexes that retain substantial conformational flexibility [42], [43]. Additionally, numerous studies demonstrate that IDRs often remain disordered despite ligand or partner engagement, and that small perturbations rarely induce the formation of stable secondary or tertiary protein structure [44]. For CARs, this distinction is critical: the intracellular CD3ζ tail is experimentally established to be both intrinsically disordered and highly dynamic, both in isolation and when associated with membranes [35], [45], making a classical structural “switch” mechanism biophysically unlikely. Instead, consistent with modern theories of dynamic allostery, our MD simulations indicate that antigen binding modulates the dynamical properties of the receptor, altering flexibility, spatial sampling, and long-range coupling, rather than inducing a stable conformational rearrangement. Such redistribution of domain dynamics, rather than formation of a new ordered structure, may represent the principal mechanism of signal propagation in CARs and other receptor systems governed by intrinsically disordered domains [46], [47].

Lastly, recent advances published in Computational and Structural Biotechnology Journal further underscore the growing role of computational modeling in oncology and therapeutic engineering. Huang et al. developed an AI-assisted framework for HER2 scoring in urothelial carcinoma, demonstrating how computational methods can resolve diagnostic ambiguities arising from heterogeneous membrane expression [48]. Liu et al. applied multi-omics integration and machine-learning classification to stratify HER2 + /ER+ breast cancers, illustrating the power of computational approaches for dissecting clinically relevant HER2-associated tumor heterogeneity [49]. Zhang et al. provided additional methodology perspectives relevant to CAR engineering and receptor modeling within MD/AI workflows [35]. Chen et al. further summarized how MD simulations, AI-augmented modeling, and multi-scale computational strategies are accelerating therapeutic protein design and optimization [50]. Together, these studies highlight a broader trend: computational techniques are increasingly indispensable for understanding HER2 biology, therapeutic targeting, and receptor behavior. Our work extends this trajectory by providing, to our knowledge, the first atomistic investigation of a full-length anti-HER2 CAR within a membrane environment, bridging diagnostics, molecular engineering, and receptor-level mechanistic modeling. By contextualizing CAR activation within this emerging computational HER2 landscape, our proposed BIDDS mechanism contributes a structural-dynamic hypothesis that complements and expands upon these AI- and MD-driven advances.

## CRediT authorship contribution statement

**Stefi Lao:** Writing – original draft, Formal analysis, Data curation. **Leah Davis:** Writing – original draft, Investigation, Formal analysis. **Mariya Hryb:** Writing – original draft, Investigation, Formal analysis. **Chun Wu:** Writing – review & editing, Writing – original draft, Supervision, Project administration, Funding acquisition, Conceptualization. **Xiaoyang Mou:** Writing – review & editing, Supervision. **Mary Staehle:** Writing – review & editing, Supervision. **Nicholas J Paradis:** Writing – original draft, Formal analysis.

## Declaration of Competing Interest

The authors declare that they have no known competing financial interests or personal relationships that could have appeared to influence the work reported in this paper.

## Footnotes

Appendix A Supplementary data associated with this article can be found in the online version at doi:10.1016/j.csbj.2025.12.017.
