# Supplementary material for: Elucidating HER2-directed chimeric antigen receptor (CAR) activation mechanism using homology modeling and all-atom molecular dynamics simulation
Source: Comput Struct Biotechnol J. 2026 Jan 2;31:276–88. doi: 10.1016/j.csbj.2025.12.017 (PMC12813887; doi:10.1016/j.csbj.2025.12.017)
Supplement: Supplementary file 1 — Supplementary material [file mmc1.pdf]

Supporting Information

Elucidating HER2-directed chimeric antigen receptor (CAR) activation mechanism using homology modeling and all-atom molecular dynamics simulation.

Mariya Hryb[2#], Leah Davis[1#], Stefi Lao[2#], Nicholas J Paradis[2#], Mary Staehle[1], Xiaoyang Mou[2] and Chun Wu[2,3*]

[1]Department of Biomedical Engineering, Henry M. Rowan College of Engineering

Departments of [2]Chemistry & Biochemistry and [3]Biological Biomedical Sciences, College of Science and Mathematics, Rowan University, Glassboro, NJ, 08028 USA

# Contribute equally

[*] To whom correspondence should be addressed: Chun Wu: wuc@rowan.edu

Supporting Experimental Methods

Prediction of natural disorder region by PONDR

Online Predictor of Natural Disordered Regions (PONDR)[1] was used to predict disordered protein regions for full HER2, CD8α, 4-1BB, and CD28 (**Fig. S6-S9**). The VL-XT output was used to describe disordered regions and refers to the merger of three predictors, one trained on **V**ariously characterized **L**ong disordered regions and two trained on **X**-ray characterized **T**erminal disordered regions.[1]

MD System Setup

The Maestro Membrane Builder was used to generate a membrane of phosphatidylcholine (POPC) lipids [2], place the TM of the anti-HER2 CAR with/without AG (apo-form and holo-form) into the POPC membrane and solvate the system in an orthorhombic water box with a buffer distance of 10 Å using simple point-charge (SPC) water model.[3] The membrane was constructed purely of POPC lipids and the TM domain of anti-HER2 CAR systems was inserted into the membrane bilayer parallel to the orientation of the POPC lipids. Then, each of the system was neutralized using Na^+ ions and a salt concentration was added at a concentration of 0.15 M NaCl. OPLS3 force field [4, 5] was used to model the receptor-ligand-lipid complex. The total system size after building was: (230Åx156Åx227Å) for the apo system and (230Åx165Åx257Å) for the holo system. The detailed information on the apo-form and the holo-form molecular dynamics systems is summarized in **Table S8**. While the pure POPC membrane composition does not faithfully represent those of real cell membranes and may not entirely account for any unobserved membrane-CAR-T protein interactions, we chose this system for two reasons. 1) Reported mutagenesis studies by DeFord-Watts et al. and Zhang et al. suggest that mutations of arginine and

lysine residues in PBRs to alanine likely do not significantly impact their associations with the biological membrane. Hence, we do not expect that changes in the membrane composition would significantly impact the membrane association observed in our MD simulations in each of the systems. 2) A POPC-pure membrane provides a neutrally-charged lipid environment to serve as a baseline for decoupling lipid electrostatic-protein interactions from MD simulations, which focus primarily on protein dynamics.

MD Simulation

All MD simulations were run using Desmond simulation program for the relaxation and the first 10 ns production run and using Anton2 for the remaining production run with a total of three independent runs per system (i.e., 5.4 μs for the apo-form system, and 10.7 or 5.4 μs for the holo-form system, for a combined simulation time of 37.7 μs). Multiple simulation runs were performed to 1) ensure convergence is achieved across each independent run, indicating system equilibration; 2) sample slow conformational modes within the protein-membrane system and 3) reduce potential bias arising from any individual simulation runs. The simulation protocol is the same as that in our previous studies of membrane receptors.[6] Here, we just give a brief description. The systems were relaxed using a relaxation protocol consisting of multiple stages: 1) Brownian dynamics simulation with a constant number of atoms, volume, and temperature (NVT) ensemble at a temperature of 10 K for 100 ps.; 2) Simulation under NVT ensemble at a temperature of 10 K with restraints on solute heavy atoms for 12 ps.; 3) Simulation under NVT ensemble with a temperature at 10 K and restraints on solute heavy atoms for 12 ps.; 4) Simulation with a constant number of atoms, pressure, and temperature (NPT) ensemble with a temperature at 10 K, a pressure of 1 bar and restraints on solute heavy atoms for 12 ps.; 5) Simulation under NPT with a

temperature at 310 K, pressure 1 bar and no restraints for 10 ns. starting from different random seeds to generate different initial velocities for each independent run. The default protocol was used to carry out a production run under the NPT ensemble at Anton 2. The Nosé-Hoover chain coupling scheme [7] and thermostat with a coupling constant of 1.0 ps. was used to control the temperature. The Martyna-Tuckerman-Klein chain coupling scheme [7] and barostat was used to control the pressure with a coupling constant of 2.0 ps. M-SHAKE [8] applied to constrain all bonds connecting hydrogen atoms, enabling a 2.0 fs. time step in the simulations. The k-space Gaussian split Ewald method [9] was used to treat long-range electrostatic interactions under periodic boundary conditions (charge grid spacing of ~1.0 Å, and direct sum tolerance of 10^{-9}). The cutoff distance for short-range non-bonded interactions was 10 Å, and the long-range van der Waals interactions was based on a uniform density approximation. Non-bonded forces were calculated using an r-RESPA integrator [10] to reduce the computation, where the short range forces were updated every step and the long range forces were updated every three steps. Frames were saved at 500.0 ps. intervals for trajectory analysis.

Post MD Simulation Analysis

To check the convergence of MD simulations, stabilization of Cα protein and ligand Root-mean squared deviation (RMSD) values, Radius of Gyration (Rgyr) and secondary-structure content over time was checked (i.e. no significant, random fluctuations in Cα protein and ligand RMSD values). In short, RMSD is the computed average of the deviation of select atomic coordinates at any timepoint relative to their initial atomic coordinates at the start of the simulation trajectory. The RMSD values are plotted across the simulation timespan. The start of the production run can reveal large deviations in RMSD values (spikes: increases; valleys: decreases)

as the system explores the simulation space. Given sufficient simulation time, the RMSD values for the system should stabilize and produce a relatively flat RMSD line, indicating the simulation has reached convergence. The Simulation Interactions Diagram (SID) tool in the Schrodinger package and VMD[11] was also used to analyze the simulation data. The properties obtained from the simulation include: RMSD, End to End distance, Rgyr, ligand-protein contacts (hydrogen bond, hydrophobic, ionic, and water-bridge contacts), protein Secondary Structure Element (SSE), and Root Rean Square Fluctuation (RMSF). The distribution of extracellular (AB) and cytoplasmic regions (CS and SI) was obtained using the position of the middle resides for extracellular (113), intracellular region including CS and SI combined (393), CS only (337), and SI only (414) for apo-form and holo-form systems.

Trajectory Clustering Analysis

Desmond trajectory clustering tool[36] was used to group complex structures from the first MD trajectories of each system (5.4 μs for the apo-form system and 10.7 μs for the holo-form system). A backbone RMSD matrix was used as a structural dissimilarity metric, and the average-linkage hierarchical clustering was selected as the clustering method with 2.5 Å merging distance cutoff. The centroid structure in the most abundant clusters was used to represent the structural family.

Residue Interaction Network Analysis

For creation of the network map consisting of nodes and edges, Cα atoms in the protein were considered as nodes and edges were drawn if nodes were within a cutoff distance of 4.5 Å for at least 75% of the MD simulation trajectory. The minor effect of this cutoff distance of 4.5 Å on network properties is already supported.[46] The CARMA tool[47] was used to calculate the cross

correlation map of Cα atoms of the CAR holo-form complexes over the entire trajectory timespan. Edge distances (d_{ij}), which define the probability of information transfer across a given edge, are derived from the pairwise correlations (C_{ij}) between the Cα atoms, where C_{ij} is the probability of transfer information across a given edge [46]:

$$d_{ij} = -\log(|C_{ij}|)$$

$$C_{ij} = \frac{\langle \Delta^{\rightarrow} r_i(t) \cdot \Delta^{\rightarrow} r_j(t) \rangle}{\left(\Delta^{\rightarrow} r_i(t)^2 \cdot \Delta^{\rightarrow} r_j(t)^2\right)^{1/2}}$$

Where $\Delta^{\rightarrow} r_i(t) = \vec{r}_i(t) - \langle \vec{r}_i(t) \rangle$ and $\vec{r}_i(t)$ is the position of the atom corresponding to the ith node[46]. Protein domain networks were visualized using the "NetworkView" module[48] of the VMD. We then selected the start and sink nodes before implementing the "subopt" program to calculate optimal and suboptimal paths between these nodes. The networks are weighted based on 1) the correlated motions of the system in the simulation trajectory and on 2) the strength on interactions within a single structure as demonstrated by Vishveshwara and cowworkers.[49, 50] Dynamic network models have been demonstrated to effectively decipher allosteric and communication pathways in transmembrane proteins[51].

Supporting Results

RMSD results reveal a significant conformational difference of CAR between the apo-form and holo-form at the domain and subdomain level. Cα-RMSD of the CAR receptor and its domains of apo-form and holo-form systems after fitting to TM only were compared to identify the detailed conformational difference at its domains due to AG binding (**Fig. 2**). The receptor

RMSD of the holo-form (74.6±4.0 Å) was significantly greater than the apo-form (48.7±6.7 Å), however each domain exhibits different trends, as is seen with AB (apo-form: 22.7±3.2 Å; holo-form: 92.4±4.8 Å), HI (apo-form: 39.7±5.3 Å; holo-form: 36.6±4.9 Å), TM (apo-form: 4.5±0.4 Å; holo-form: 2.3±0.6 Å), CS (apo-form: 97.7±13.5 Å; holo-form: 23.2±3.4 Å) and SI (apo-form: 77.0±10.1 Å; holo-form: 44.7±9.2 Å) (**Table S9**). Clearly, AB, CS, and SI contribute the most to the overall receptor conformational difference between apo-form and holo-form. The relative contributions of intra-domain differences (domain aligned to itself) and inter-domain differences (intra-domain difference - overall RMSD difference**) (Table S9**) between the apo-form and holo-form systems was also calculated (**Fig. S27-S30**). One note is that since both overall and intra-domain change was calculated by fitting to TM, the inter-domain difference for TM is 0 Å. Inter-domain differences for AB (71.0 Å), HI (-2.0 Å), CS (72.6 Å) and SI (29.7 Å) suggest that 1). The overall conformation difference between the two systems for AB (71.5 Å) is from the inter-domain part (71.0 Å); 2). the difference for HI is insignificant; 3). The difference for CS (75.6 Å) is from the inter-domain part (72.6 Å), 4). The difference for SI (32.3 Å) is mainly from the inter-domain part (29.7 Å) **(Table S9**).

AB-to-TM distance decreased while TM-to-SI distance increased in holo-form. Last snapshot structures of the anti-HER2 CAR holo-form and apo-form is shown in three view perspectives (**Fig. S43**). In the holo-form, AB in red exhibits a bending toward TM. Furthermore, both CS and SI appear to have a less extended conformation in holo-form compared to apo-form, thereby shortening the distance between TM and SI (**Fig. S43**). The distance between various CAR parts in the anti-HER2 CAR in apo-form and holo-form was measured for the combined three trajectories (**Fig. S44)** and their values at converged were calculated (**Table S11)**. AB to

TM distance was greater in the apo-form (54.8±3.2 Å) than in the holo-form (49.9±2.9 Å), with a difference of 4.9 Å (**Table S11**). However, the opposite was observed between SI to TM, as the distance was greater in the holo-form (56.1±6.8 Å) compared to apo-form (43.7±2.7 Å), with a difference of 12.4 Å (**Table S11**).

Rgyr demonstrated lower values for N-terminal, and higher values for C-terminal CAR portions in the holo-form compared to apo-form. To characterize the size change of the receptor, Rgyr values of the CAR structure in apo-form and holo-form were calculated for all three combined trajectories and shown in **Table S12** and **Fig. S45**. Overall, compared to the apo-form, the holo-form demonstrates smaller Rgyr values with a decrease by 2.6 Å for the receptor. However, Rgyr of N-terminus becomes smaller in holo-form confirmed by 1.0 Å decrease, while Rgyr of C-terminus increases by 1.1 Å in response to the binding of antigen.

MD simulations reveal AB and TM were structurally ordered, HI became more disordered, and CS and SI significantly decrease in turn conformation and converted into α-helix and coil. To investigate differences in anti-HER2 CAR protein secondary structure, SSE analysis was performed for both apo-form and holo-form simulation systems for the combined (**Fig. 7**) and individual trajectories (**Fig. S47**). SSE values for the anti-HER2 CAR receptor and each domain were compared between the homology model (HM, initial structure) and the MD apo-form structure (**Table S15)**. SSE analysis was also performed between the MD-apo-form and MD-holo-form structures (**Table S16**). Overall, AB did not show significant change, maintained its initial β-sheet rich conformation and remained ordered; HI and SI exhibited conversion of their helical content into coil and β-extended confirmation and became disordered; TM showed

conversion of random coil into α-helix and became more ordered (**Table S15**). No significant difference in SSE was observed for AB between apo-form and holo-form (**Table S16**).

Clustering analysis reveals significant conformation differences between holo-form and apo-form. Clustering analysis for the first trajectory based on anti-HER2 Cα-RMSD between conformations grouped the holo-form into 38 clusters and the apo-form into 33 clusters, both with 2.5 Å RMSD as the cutoff distance. Representative structures of the most abundant clusters, whose population is ≥ 2% of total population, are shown in **Fig. S48-49**. Two key differences were observed for HI. First, the hinge region is less extended in the holo-form, causing a smaller distance between AB and the membrane and a less extended conformation for the intracellular part (**Fig. S48-49**). Second, a smaller distance between the C-terminus and membrane in the holo-form is due to its more compressed conformation compared to the apo-form (**Fig. S48-49**). The difference in TM is small between the two systems. Overall, the difference between snapshot structures of holo-form and apo-form provided by the clustering analysis (**Fig. S48-49**) show similarity to the last snapshot structures of MD simulation for holo-form and apo-form in **Fig. S43** as well as the end-to-end distance and Rgyr results in **Table S11-12**.

PBRs in ITAMS are highly conserved in CD3ζ and non-mammalian species. Sequence analysis for the protein family of PF02189 and PF11628 of CD3ζ ITAMs was done to confirm ITAMs are indeed conserved as shown by the HMM logo (**Fig. S14**) and the seed sequence alignment (**Fig. S15**). Additionally, SI PBRs previously defined[12] were also matched with non-mammalian organisms and demonstrated that key residues within PBRs, including arginine and lysine, are generally highly-conserved (**Fig. S16**).

The list of e-component files and their short description.

Filename	Content
desmond_setup_1_HER2_CAR_T_apo.mae	Initial structure of the apo protein
desmond_setup_1_HER2_CAR_T_Holo.mae	Initial structure of the holo protein
HER2-car-t_apo-out.cms	Initial file for MD simulation of the apo system
HER2-car-t-out.cms	Initial file for MD simulation of the holo system
MD_mem.cfg	MD simulation protocol
Apo_clust[1-12]_aligned.mae	Representative apo protein structures of top 12 clusters
Holo_Clust[1-22].mae	Representative holo protein structures of top 22 clusters

Table S1. FDA-approved CAR T cell therapies.

CAR T cell	Brand name	Company	Approval Date	Target
tisagenlecleucel	Kymriah	Novartis Pharmaceuticals Corp.	08/30/2017	CD19
axicabtagene ciloleucel	Yescarta	Kite Pharma, Inc.	10/18/2017	CD19
brexucabtagene autoleucel	Tecartus	Kite Pharma, Inc.	07/24/2020	CD19
lisocabtagene maraleucel	Breyanzi	Bristol-Myers Squibb Company	02/05/2021	CD19
idecabtagene vicleucel	Abecma	Bristol-Myers Squibb Company	03/26/2021	BCMA
ciltacabtagene autoleucel	Carvykti	Janssen Biotech, Inc.	02/28/2022	BCMA

Table S2. Various Properties of HER2 antigen and five anti-HER2 CAR domains.

**Domain Name**	**Code**	**Length of sequence**	**Range**	**Original Human Protein**	**UniProt ID**	**PDB ID**
Extracellular domain of the HER2 antigen	AG	192	1-192	HER2	P04626	3H3B
Anti-body	AB*	247	1-247	Anti-HER2-scFv	N/A	3H3B (Mouse Anti-HER2-scFv)
Hinge	HI	45	248-292			No
Transmembrane	TM	24	293-316	CD8α	P01732	No
Costimulatory	CS	42	317-358	CD137	Q07011	No
Signaling	SI	112	359-470	CD3ζ	P20963	No

*AB: Light chain motif (1-109) and linker (110-124) and heavy chain motif (125-247).

Table S3. The sequence of the five anti-HER2 CAR domains.

Name	pfam ID (range)	Sequence
**AG**	P04626 (22-214)	GSTQVCTGTDMKLRLPASPETHLDMLRHLYQGCQVVQGNLELTYLPTNASLSFLQDIQEVQGYVLIAHNQVRQVPLQRLRIVRGTQLFEDNYALAVLDNGDPLNNTTPVTGASPGGLRELQLRSLTEILKGGVLIQRNPQLCYQDTILWKDIFHKNNQLALTLIDTNRSRACHPCSPMCKGSRCWGESSEDCQS
**AB**		DIQMTQSPSSLSASVGDRVTITCRASQDVNTAVAWYQQKPGKAPKLLIYSASFLYSGVPSRFSGSRSGTDFTLTISSLQPEDFATYYCQQHYTTPPTFGQGTKVEIKRTGGGGSGGGGSGGGGSEVQLVESGGGLVQPGGSLRLSCAASGFNIKDTYIHWVRQAPGKGLEWVARIYPTNGYTRYADSVKGRFTISADTSKNTAYLQMNSLRAEDTAVYYCSRWGGDGFYAMDYWGQGTLVTVSSAAA
**HI and TM**	PF07686 (138-182, 183-206)	TTTPAPRPPTPAPTIASQPLSLRPEACRPAAGGAVHTRGLD<br>FACDIYIWAPLAGTCGVLLLSLVITLYC
**CS**	Q07011 (214-255)	KRGRKKLLYIFKQPFMRPVQTTQEEDGCSCRFPEEEEGGCEL
**SI**	P20963 (52-164)	RVKFSRSADAPAYKQGQNQLYNELNLGRREEYDVLDKRRGRDPEMGGKPQRRKNPQEGLYNELQKDKMAEAYSEIGMKGERRRGKGHDGLYQGLSTATKDTYDALHMQALPPR

*AB Linker (110-124) in pink. TM in yellow. Polybasic region (PBR) 1 (395-399), 2 (406-410) and 3(435-442) in blue and Q of PBR2 in red was deleted. Immunoreceptor tyrosine-based activation motif (ITAM) 1 (379-393), 2 (418-432) and 3 (446-462) in green.

Table S4. HER2 antigen domain (AG) and PDB structures (UniProt ID: P04626).

PDB ID	Method	Resolution	Position
AG	n/a	n/a	22-214
1MFG	X-ray	1.25 Å	1247-1255
1MFL	X-ray	1.88 Å	1247-1255
1MW4	NMR	n/a	1135-1144
1N8Z	X-ray	2.52 Å	23-629
1QR1	X-ray	2.40 Å	654-662
1S78	X-ray	3.25 Å	23-646
2A91	X-ray	2.50 Å	22-530
2JWA	NMR	n/a	641-684
2KS1	NMR	n/a	641-684
2L4K	NMR	n/a	1135-1144
2N2A	NMR	n/a	644-700
3BE1	X-ray	2.90 Å	23-646
**3H3B**	**X-ray**	**2.45 Å**	**23-214**
3MZW	X-ray	2.90 Å	23-646
3N85	X-ray	3.20 Å	23-646
3PP0	X-ray	2.25 Å	703-1029
3RCD	X-ray	3.21 Å	713-1028
3WLW	X-ray	3.09 Å	23-586
3WSQ	X-ray	3.50 Å	23-586
4GFU	X-ray	2.00 Å	1246-1252
4HRL	X-ray	2.55 Å	24-219
4HRM	X-ray	3.20 Å	24-219
4HRN	X-ray	2.65 Å	529-625
4NND	X-ray	2.50 Å	1109-1114
5K33	X-ray	3.30 Å	23-629
5KWG	X-ray	4.30 Å	23-653
5MY6	X-ray	2.25 Å	24-645
5O4G	X-ray	3.00 Å	23-628
5OB4	NMR	n/a	641-684

5TQS	X-ray	1.88 Å	1218-1228
6ATT	X-ray	3.77 Å	23-652
6BGT	X-ray	2.70 Å	1-652
6J71	X-ray	2.92 Å	22-639
6LBX	X-ray	2.03 Å	531-626
6OGE	EM	4.36 Å	23-644
7JXH	X-ray	3.27 Å	703-1024
7MN5	EM	2.93 Å	1-1029
7MN6	EM	3.09 Å	1-1029
7MN8	EM	3.45 Å	1-1029
7PCD	X-ray	1.77 Å	703-1029

Table S5. Target CD8α signaling domain and PDB structures (UniProt ID: P01732).

PDB ID	Method	Resolution	Position
n/a	n/a	n/a	138-182 (HI)
n/a	n/a	n/a	183-206 (TM)
1AKJ	X-ray	2.65 Å	22-141
1CD8	X-ray	2.60 Å	22-135
1Q69	NMR	n/a	209-227
2HP4	X-ray	2.10 Å	22-135
3QZW	X-ray	2.80 Å	22-135

*CD8α HI and TM.

Table S6. Target CD137 (4-1BB) signaling domain and PDB structures (UniProt ID: Q07011).

Target/PDB ID	Method	Resolution	Positions
CD137(4-1BB)	n/a	n/a	214-255
6A3V	X-ray	3.39 Å	24-180
6A3W	X-ray	2.00 Å	24-180
6BWV	X-ray	2.40 Å	24-160
6CPR	X-ray	2.70 Å	23-160
6CU0	X-ray	3.20 Å	23-160
6MGP	X-ray	2.13 Å	25-162
6MHR	X-ray	2.80 Å	25-162
6MI2	X-ray	2.72 Å	25-162
6Y8K	X-ray	2.01 Å	24-160
7D4B	X-ray	3.14 Å	25-162

Table S7. Target CD3ζ signaling domain and PDB structures (UniProt ID: P20963).

Target/PDB ID	Method	Resolution	Positions
CD3ζ	n/a	n/a	52-164
1TCE	NMR	n/a	24-180
1YGR	X-ray	2.90 Å	24-180
2HAC	NMR	n/a	24-160
2OQ1	X-ray	1.90 Å	23-160
3IKS	X-ray	2.05 Å	23-160
3IOZ	X-ray	3.70 Å	25-162
4XZ1	X-ray	2.80 Å	25-162
6JXR	EM	3.70 Å	1-164* (22-57)

*The full length of CD3ζ was used in the study, but only 22-57 was modeled in the PDB.

Table S8. Molecular dynamics simulations system information of the anti-HER2 CAR in apo-form and holo-form.

System ID	No. of atoms	CAR	AG	No. of Water molecules	No. of ions	No. of POPC	Box Size ABC (Å)*	No. of run	Time (μs)
**Apo-1**	353464	1	0	94968	264 Cl-/Na+	452	137.1/ 115.6/ 232.3	3	5.4
**Holo-1**	414594	1	1	111942	312 Cl-/Na+	505	161.2/ 106.0/ 252.5	1/2	10.7/5.4

*Systems were constructed in an orthorhombic-shaped box.

Table S9. Mean and standard deviation of Cα-RMSD values (Å) of the receptor parts in apo-form and holo-form over the converged region for all three combined trajectories.

	**Fit to TM**			**Fit to domain self**			**Interdomain difference* (Holo-Apo)**
**Domain**	**Holo**	**Apo**	**Overall difference |Holo-Apo|**	**Holo**	**Apo**	**Intradomain difference (Holo-Apo)**	
**anti-HER2 CAR**	74.6±4.0	48.7±6.7	**25.9**	46.4±4.4	46.0±3.8	0.4	25.5
**AB**	94.2±4.8	22.7±3.2	**71.5**	3.8±0.3	2.3±0.3	**0.5**	**71.0**
**HI**	36.6±4.9	39.7±5.3	**3.2**	9.9±1.0	15.1±0.9	**5.2**	**-2.0**
**TM**	2.3±0.6	4.5±0.4	**2.2**	2.3±0.6	4.5±0.4	**2.2**	**0.0**
**CS**	23.2±3.4	97.7±13.5	**74.5**	8.5±0.8	10.4±0.5	**1.9**	**72.6**
**SI**	44.7±9.2	77.0±10.1	**32.3**	8.7±0.8	11.3±0.9	**2.5**	**29.7**

*RMSD values for interdomain difference was calculated by subtracting RMSD fitting to domain self from that fitting to TM.

Table S10. Mean and standard deviation of Cα-RMSD values (Å) of the AG-AB complex in holo-form and its decomposition over the converged region for all three combined trajectories.

Domain	Overall Change (Fit to AG-AB complex)	Intradomain change (Fit to itself)	Interdomain change (overall-intradomain)
**AG-AB complex**	5.8±0.3	N/A	N/A
**AG**	5.7±0.5	3.4±0.1	2.3
**AB**	6.1±0.3	4.2±0.3	1.9

Table S11. Mean and standard deviation of distance values between various parts of the receptor in apo-form and holo-form over the converged regions for all three combined trajectories.

**anti-HER2 CAR Structure Domain**	**Distance (Å)**		
	**Holo**	**Apo**	**Difference |Apo-Holo|**
**AB to TM***	49.9±2.9	54.8±3.2	4.9
**SI to TM***	56.1±6.8	43.7±2.7	12.4

*Distance between the centers of the mass.

Table S12. Mean and standard deviation of radius of gyration (Rgyr) values of the receptor parts in apo-form and holo-form over the converged regions for all three combined trajectories.

anti-HER2 CAR part	Radius of Gyration (Å)		
	Holo	Apo	Difference |Apo-Holo|
All	47.3±3.2	44.6±2.0	2.6
N-terminal	19.7±0.2	20.7±0.2	1.0
C-terminal	19.7±1.0	18.6	1.1

Table S13. Average atom contacts between the three PBRs, ITAMs and tyrosine residues (TYR) of ITAM with POPC membrane for apo-form and holo-form for all three combined trajectories.

	Apo	Holo
**PBR 1**	2.1	0.1
**PBR 2**	5.7	0.4
**PBR 3**	1	0.1
**Total**	8.8	0.6
**ITAM1**	6.1	0.1
**ITAM2**	0.4	0.5
**ITAM3**	0.2	0.1
**Total**	6.7	0.6
**TYR in ITAM1**	1.9	0.2
**TYR in ITAM2**	0.2	0
**TYR in ITAM3**	0	0
**Total**	2.1	0.2

Table S14. Mean and standard deviation of Root mean squared fluctuation (RMSF) values of the receptor domains in apo-form and holo-form over the converged regions for all three combined trajectories.

**Domain Name**	**RMSF (Å)**		
	**Holo**	**Apo**	**Difference (Holo-Apo)**
**anti-HER2 CAR complex with the HER2 antigen**	N/A	N/A	N/A
**AG**	N/A	N/A	N/A
**anti-HER2 CAR**	25.9±9.4	31.6±10.9	-5.6
**AB**	27.3±5.4	32.5±8.1	-5.2
**HI**	18.0±6.1	18.0±5.0	0.0
**TM**	1.9±0.8	2.6±0.7	0.7
**CS**	17.4±4.3	14.7±3.6	+2.7
**SI**	34.5±5.8	31.2±6.3	+3.3

Table S15. Secondary Structure Elements (SSEs) of the apo-form receptor during MD simulation and of the homology model (HM) for all three combined trajectories.

Domain Name	α-helix (%)			Turn (%)			β-extended conformation (%)			Coil (%)		
	Apo MD	Apo HM	Diff (MD-HM)	Apo MD	Apo HM	Diff (MD-HM)	Apo MD	Apo HM	Diff (MD-HM)	Apo MD	Apo HM	Diff (MD-HM)
**AB**	1.0	0.0	1.0	49.7	32.4	17.3	27.6	44.9	-17.3	15.7	22.7	-7.0
**HI**	23.8	77.8	-54.0	1.0	20.0	-19.0	43.8	0.0	43.8	31.3	2.2	29.1
**TM**	74.8	95.8	-21.0	0.0	0.0	0.0	19.9	0.0	19.9	5.3	4.2	1.1
**CS**	4.0	16.7	-12.7	1.0	71.4	-70.4	72.1	0.0	72.1	22.9	11.9	11.0
**SI**	24.2	56.3	-32.1	1.1	35.7	-34.6	57.6	0.0	57.6	17.1	8.0	9.1
**Total**	12.7	27.2	-14.5	26.5	33.8	-7.3	43.1	23.6	19.5	17.6	15.3	2.3

Table S16. SSEs in apo-form and holo-form of the receptor during MD simulation for all three combined trajectories.

Domain Name	α-helix (%)			Turn (%)			β-extended conformation (%)			Coil (%)		
	Holo MD	Apo HM	Diff (MD-HM)	Holo MD	Apo HM	Diff (MD-HM)	Holo MD	Apo HM	Diff (MD-HM)	Holo MD	Apo HM	Diff (MD-HM)
**AB**	1.2	1.0	0.2	47.9	49.7	-1.8	29.7	27.6	2.1	15.2	15.7	-0.5
**HI**	29.4	23.8	5.7	1.6	1.0	0.6	39.6	43.8	-4.3	29.3	31.3	-2.0
**TM**	82.2	74.8	7.3	0.0	0.0	0.0	14.1	19.9	-5.8	3.7	5.3	-1.6
**CS**	11.2	4.0	7.3	3.7	1.0	2.7	59.3	72.1	-12.9	25.8	22.9	2.9
**SI**	21.0	24.2	-3.2	1.3	1.1	0.2	55.1	57.6	-2.5	22.6	17.1	5.5
**Total**	13.6	12.7	0.9	26.0	26.5	-0.6	41.7	43.1	-1.4	18.7	17.6	1.0

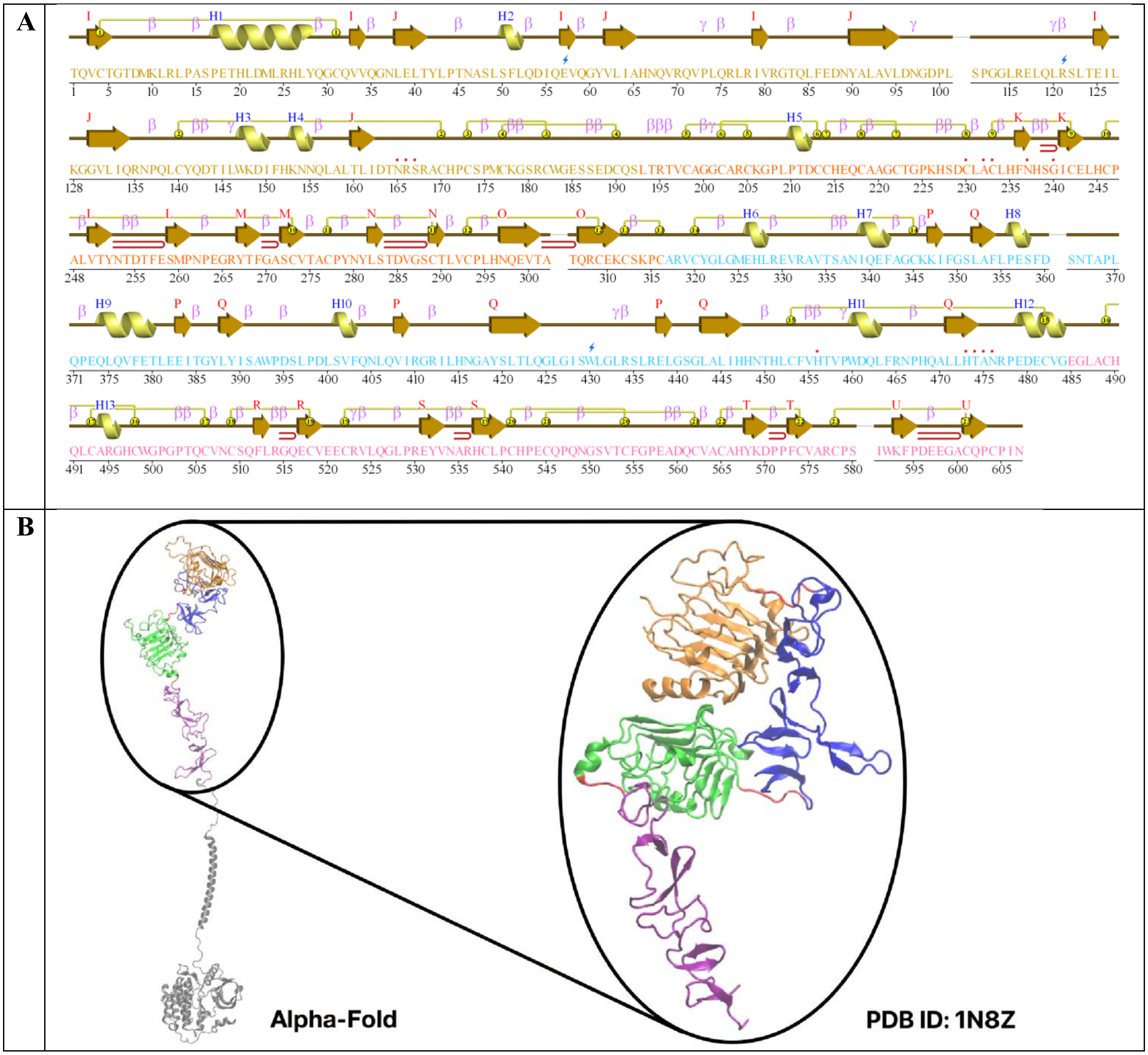

Figure S1. Primary, secondary (**A**) and tertiary structure (**B**) of HER2. PDB structure and AlphaFold prediction (P04626). **A**: Primary and Seconary Structure of the extracelllular domain of human HER2 (**PDB ID**: **1N8Z**). **B**: AlphaFold structure of full HER2 (left) and crystal structure of extracellular domain of human HER2 (**PDB ID: 1N8Z**) (right). Extracellular domain I in orange (1-192), domain II blue (199-315), domain III in green (321-485), domain in purple IV (490-607); linkers (192-198, 316-320, 486-489) in red, transmembrane and intracellular domains in grey (629-1010).

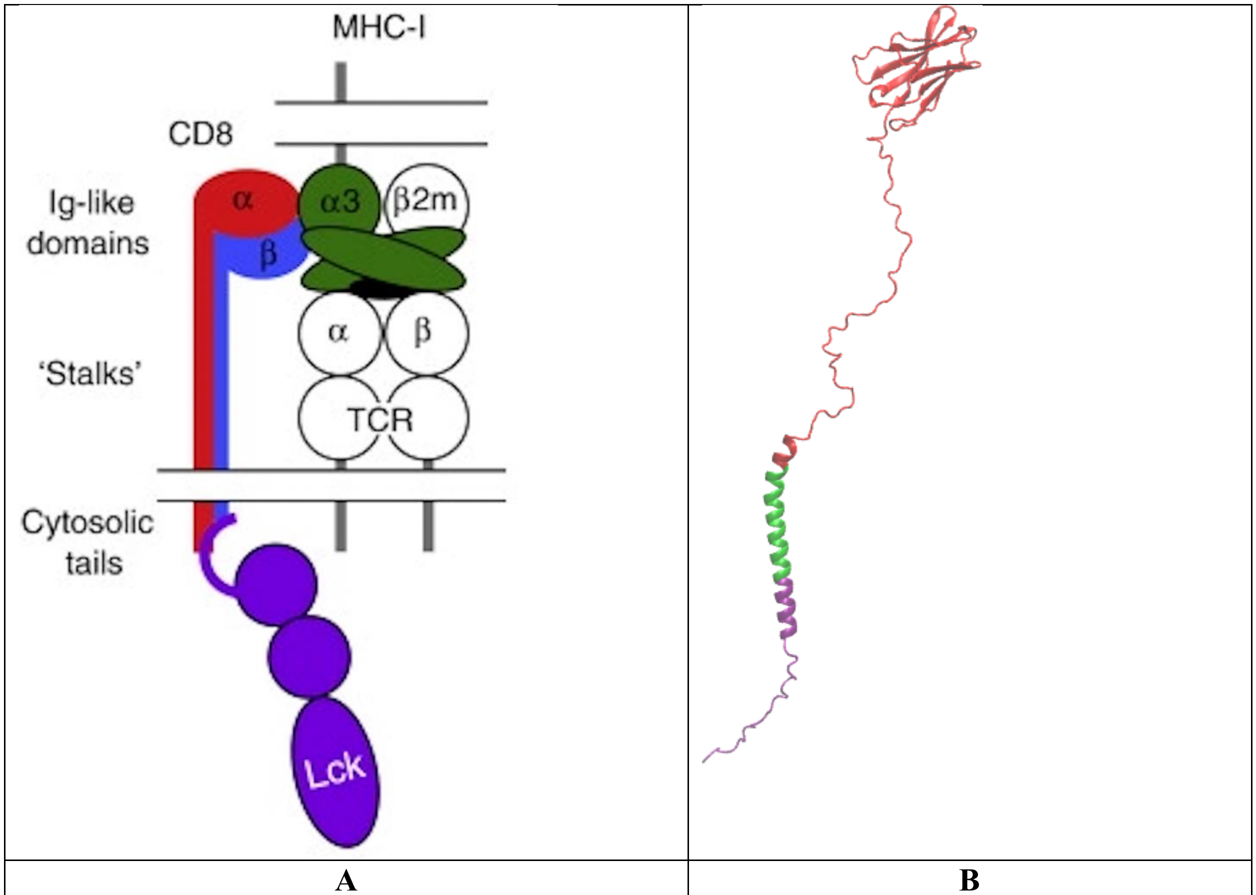

A B

Figure S2. Diagram of CD8α complex (**A**) and its high-resolution structure modified from AlphaFold prediction (**B**). **A**: CD8 dimer structure together with TCR-CD3 complex: CD8α (red) and CD8β (blue) bound to the α3 domain of MHC-I molecules (green) and Lck (purple).[13] **B**: AlphaFold prediction for CD8α. CD8α topological domains based on UniProt features (UniProt ID: P01732): extracellular domain (22-182) in red, transmembrane domain (129-166) in green, cytoplasmic domain (209-234) in purple.

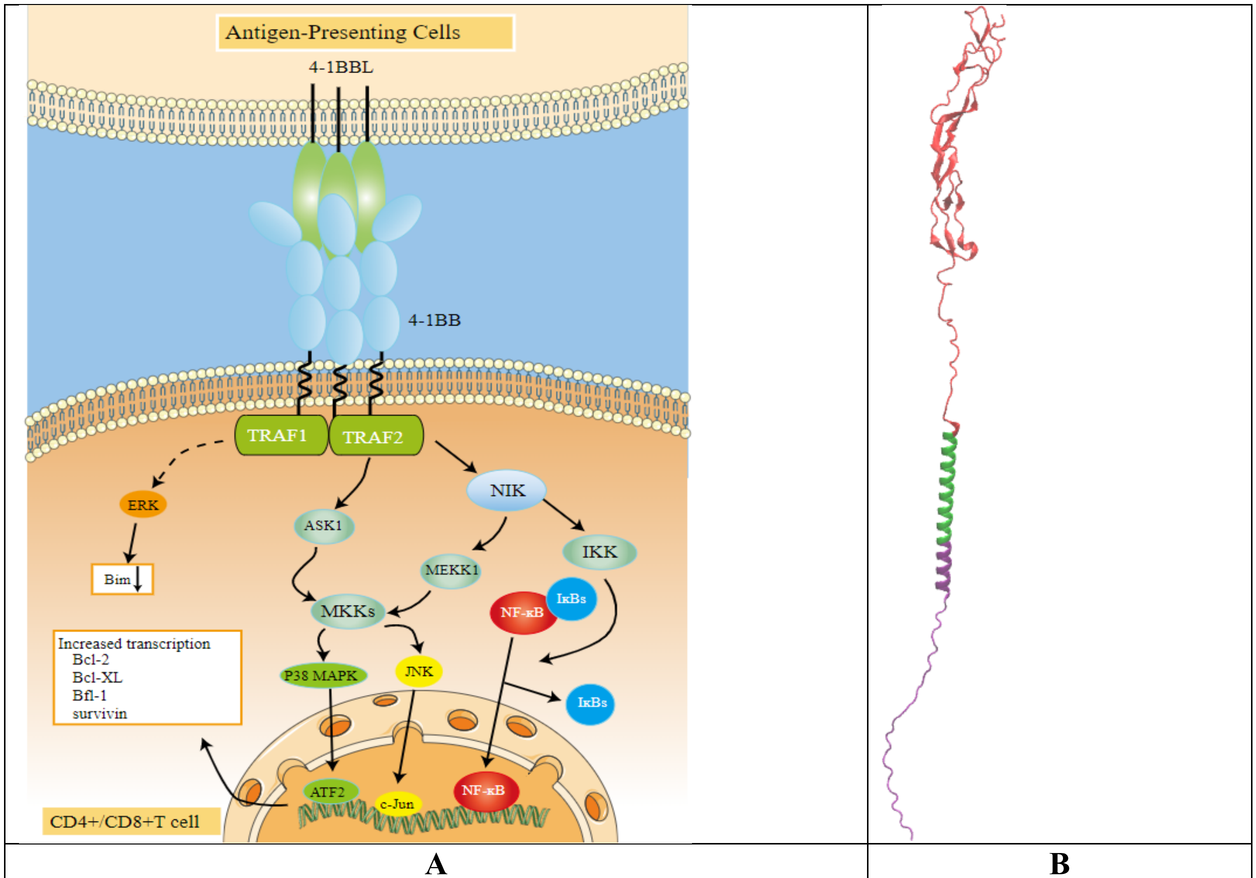

A B

Figure S3. Diagram of 4-1BB receptor activation pathways (**A**) and its high-resolution structure modified from the AlphaFold prediction (**B**). **A**: 4-1BB/4-1BBL signaling pathway: 4-1BB (blue) in complex with its ligand, 4-1BBL (green) bound to Antigen Presenting Cell. Molecular adapters, TRAF1 and TRAF2 (green), bound to 4-1BB by activating the NF-κB, AKT, p38 MAPK, and ERK pathways leading to increased production of survival genes encoding survivin, Bcl-2, Bcl-XL, and Bfl-1 and decreased expression of pro-apoptotic Bim (https://www.creative-diagnostics.com/4-1bb-4-1bbl-signaling-pathway.htm). **B**: AlphaFold prediction for 4-1BB. 4-1BB topological domains based on UniProt features (UniProt ID: Q07011): extracellular domain (24-186) in red, transmembrane domain (187-213) in green, cytoplasmic domain (214-255) in purple.

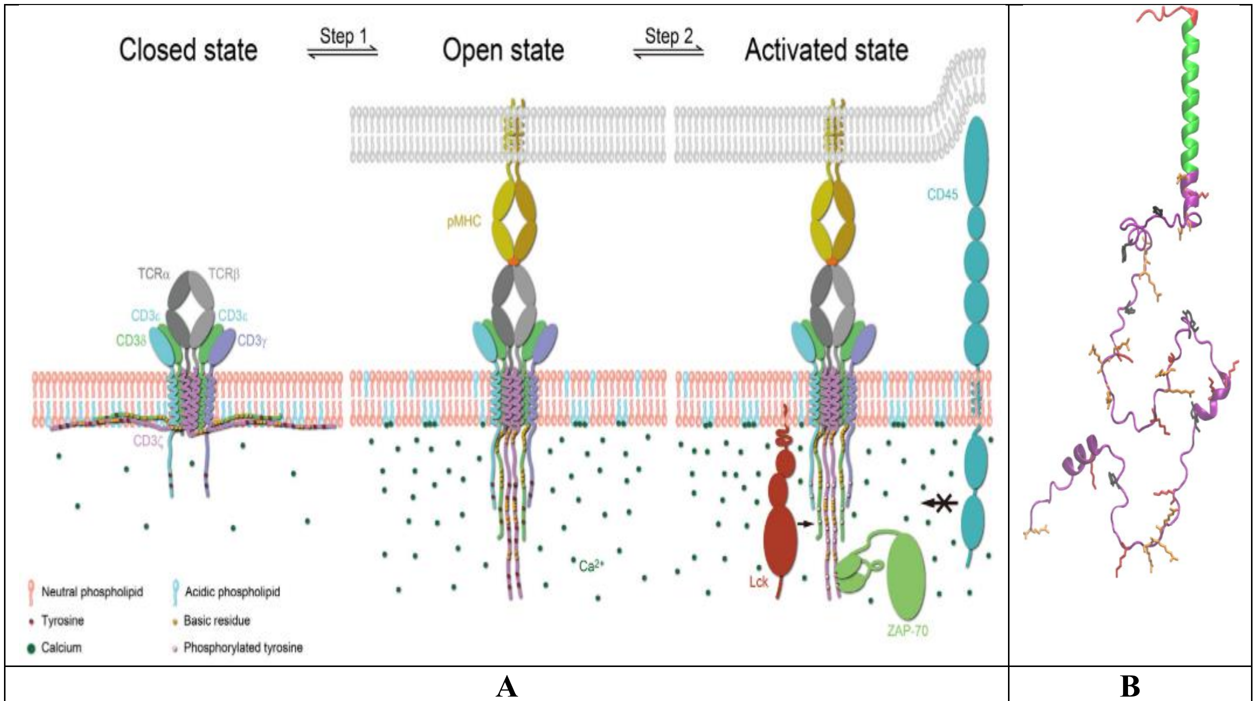

A	B

Figure S4. Two-step activation mechanism of TCR[12] Wu, W. et al and the structure of CD3ζ. **A:** Transition from closed state to open state via dissociation of CD3 cytoplasmic domains from the membrane upon AG binding via making ITAMs available for phosphorylation. Upon transition to the activated state, ITAM tyrosine residues can be phosphorylated by Lck and can further recruit downstream signaling molecule ZAP70.[12] **B:** The structure of CD3ζ based on AlphaFold prediction for CD3ζ. CD3ζ topological domains based on UniProt features (UniProt ID: **P20963**): extracellular domain (22-30) in red, transmembrane domain (31-51) in green, cytoplasmic domain (52-164) in purple, arginine residues in orange, lysine residues in red, tyrosine residues in black.

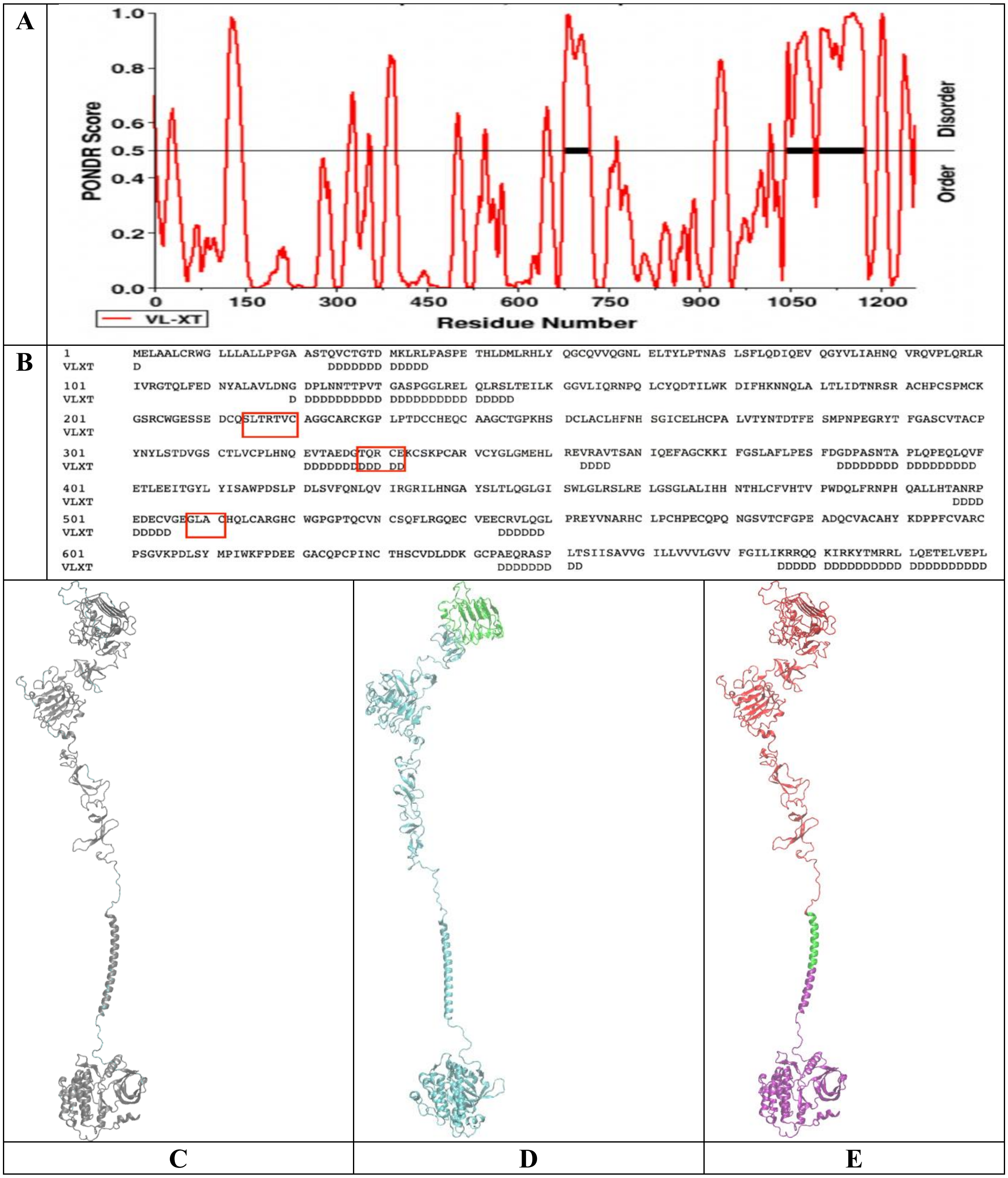

Figure S5. Disordered regions (**A-B**) of HER2 predicted by PONDR and its high-resolution structure predicted by AlphaFold (P04626) in the different color schedules (**C-E**). **A**: PONDR prediction diagram of ordered and disordered regions. **B**: PONDR sequence output of ordered and disordered regions with linkers in red (linker 1: 192-198; linker 2: 316-320, linker 3: 486-489). **C**: HER2 AlphaFold structure with PONDR predicted disordered regions highlighted in cyan (1, 24-35, 120-145, 320-331, 352-355, 382-398, 496-504, 543-548, 643-651, 675-719, 761-763, 926-943, 1014-1019, 1041-1088, 1095-1172, 1193-1209, 1232-1248, 1254). **D**: HER2

AlphaFold structure with HER2 regions used in anti-HER2 CAR as AG (22-214). **E**: HER2 AlphaFold structure with topological domains based on UniProt features (UniProt ID: P04626): extracellular domain in red (23-652), transmembrane domain in green (653-675), cytoplasmic domain in blue (676-1255).

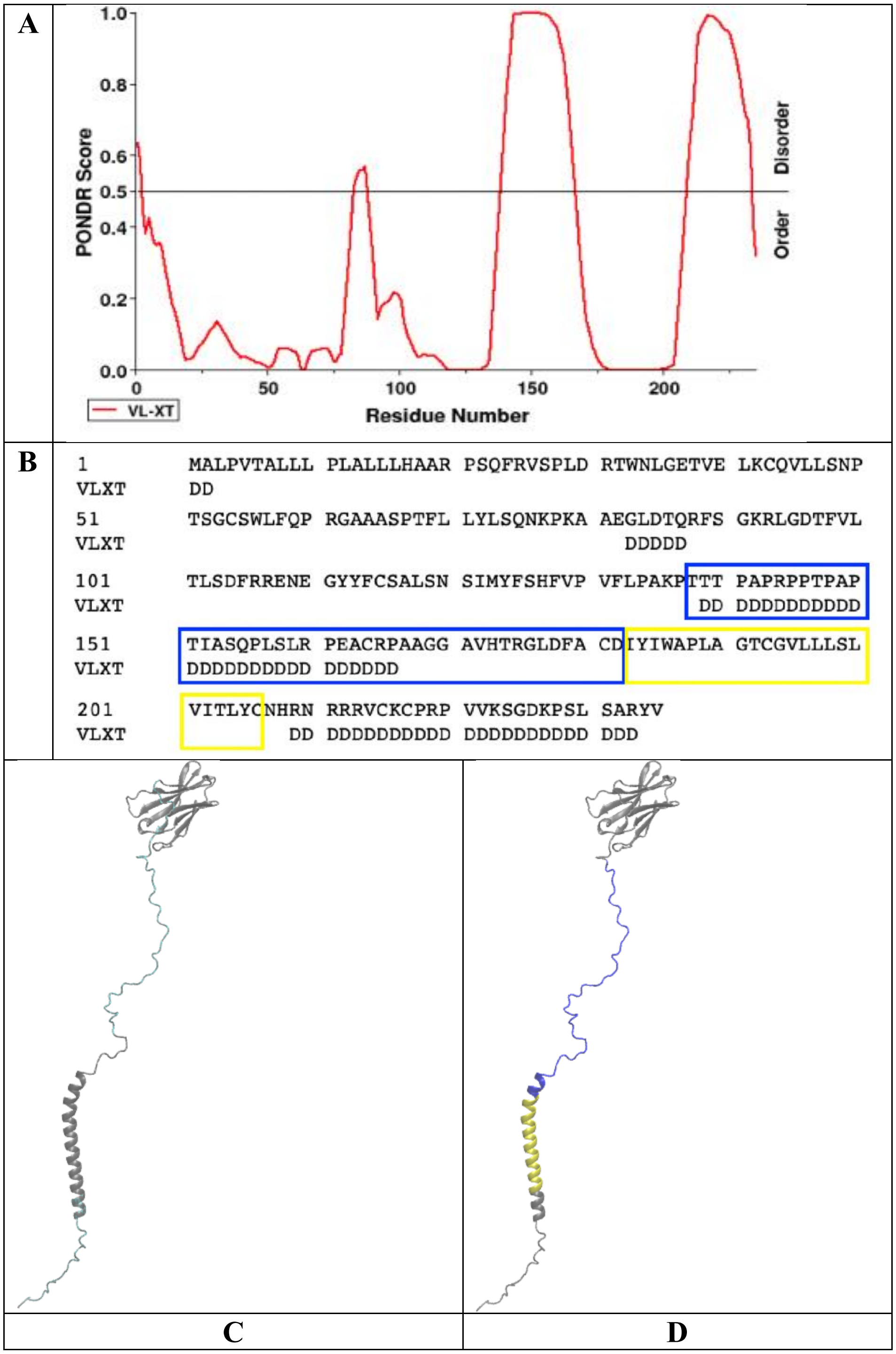

Figure S6. Disordered regions (**A-B**) of CD8α predicted by PONDR and its high-resolution structure predicted by AlphaFold (P01732) in the different color schedules (**C-D**). **A**: PONDR prediction diagram of ordered and disordered regions. **B**: PONDR sequence output of ordered and disordered regions with CD8α parts used as HI (blue) and TM (yellow) in anti-HER2 CAR. **C**: CD8α AlphaFold structure with PONDR predicted disordered regions (83-87, 129-166, 209-234) in cyan. **D**: CD8α AlphaFold structure with CD8α regions used in anti-HER2 CAR as HI (138-182) in blue and TM (183-206) in yellow.

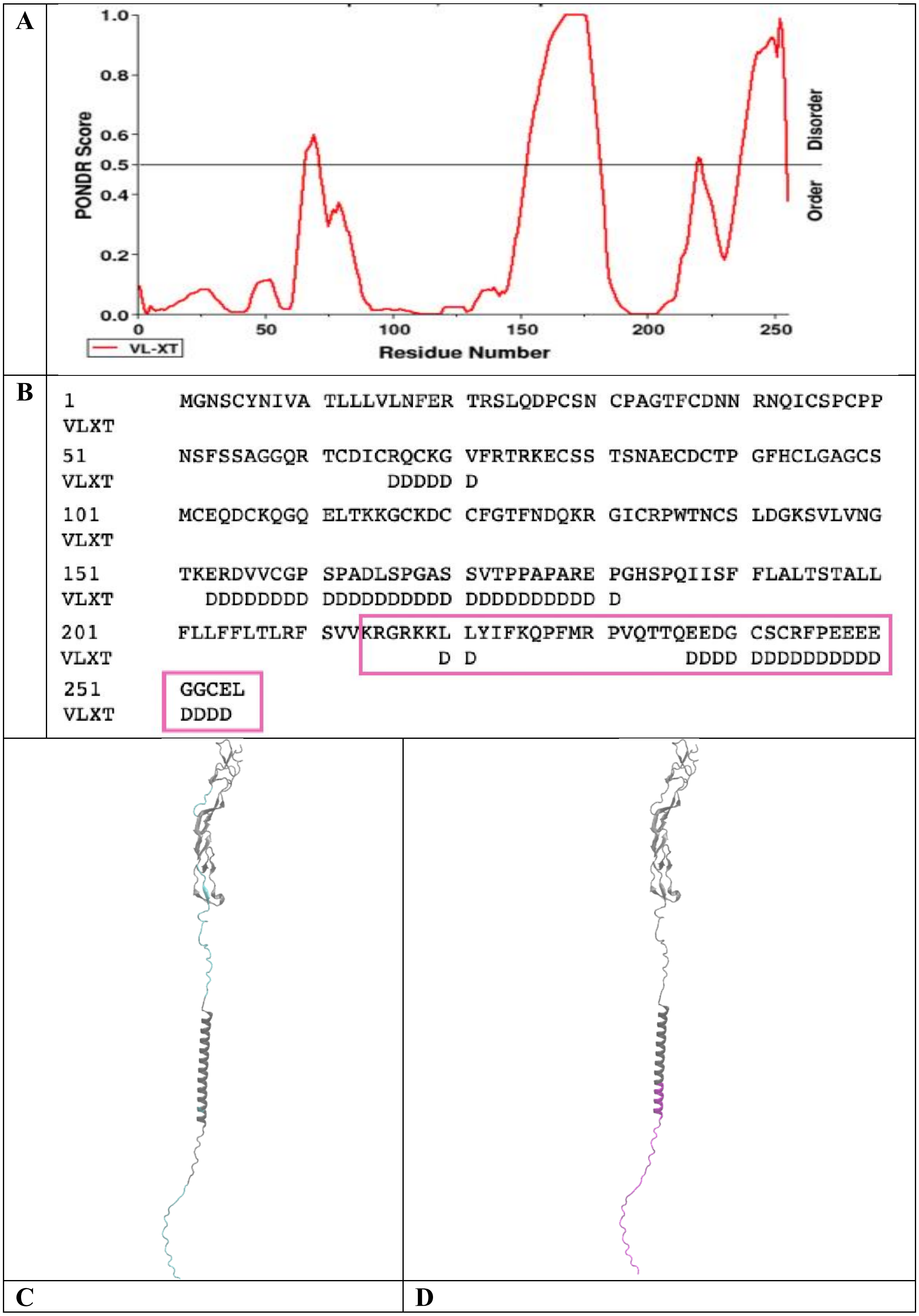

Figure S7. Disordered regions (**A-B**) of 4-1BB predicted by PONDR and its high-resolution structure predicted by AlphaFold (Q07011) in the different color schedules (**C-D**). **A**: PONDR prediction diagram of ordered and disordered regions. **B**: PONDR sequence output of ordered

and disordered regions with 4-1BB part used as CS in anti-HER2 CAR **C**: 4-1BB AlphaFold structure with PONDR predicted disordered regions (66-71, 153-181, 220-221, 237-254) in cyan. **D**: 4-1BB AlphaFold structure with 4-1BB region (214-256) used in anti-HER2 CAR as CS in pink.

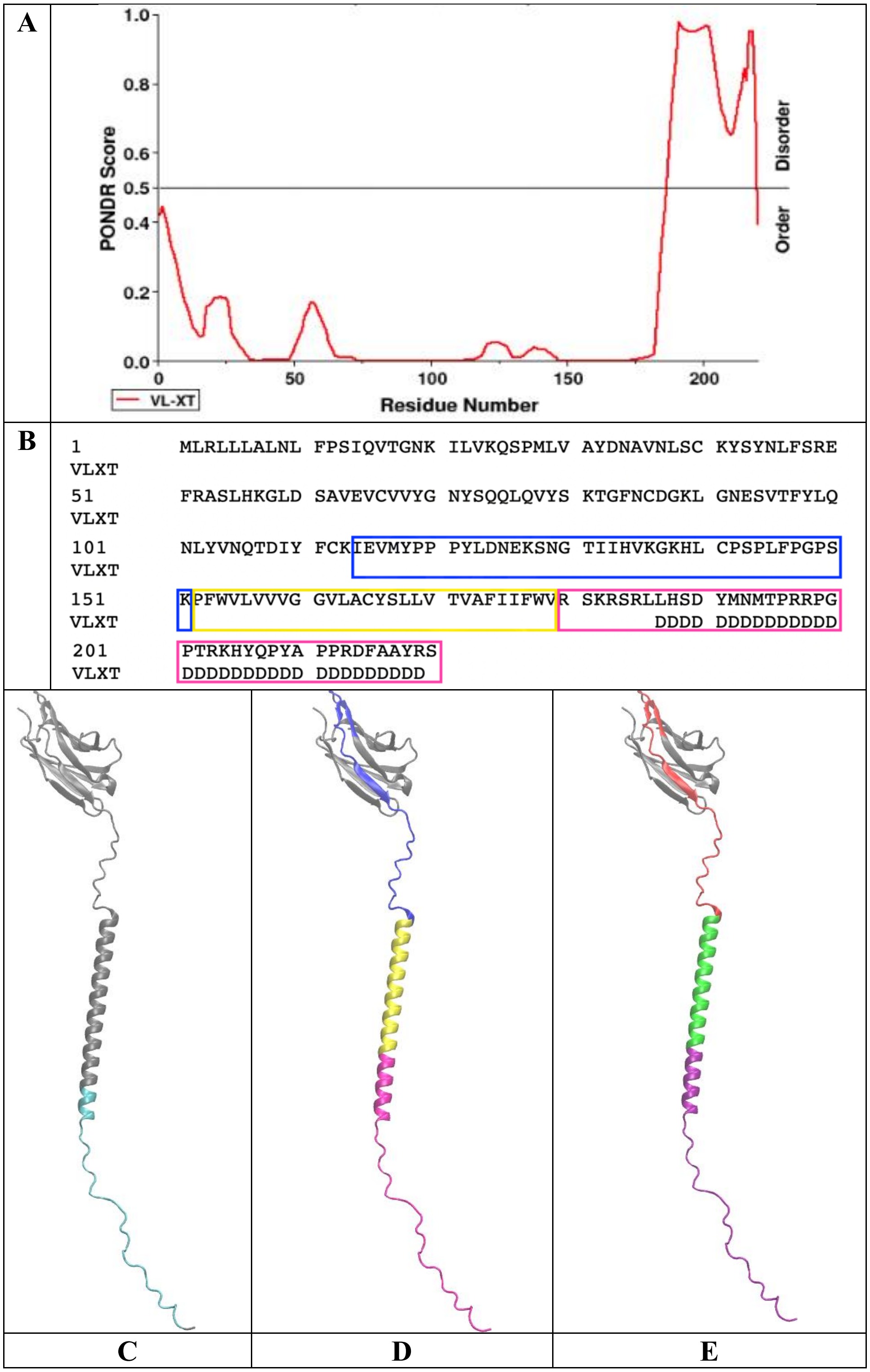

Figure S8. Disordered regions (**A-B**) of CD28 predicted by PONDR and its high-resolution structure predicted by AlphaFold (P10747) in the different color schedules (**C-D**). **A**: PONDR prediction diagram of ordered and disordered regions. **B**: PONDR sequence output of ordered and disordered regions with CD28 parts used in different anti-HER2 CARs as HI (blue), TM (yellow), CS (pink). **C**: CD28 AlphaFold structure with PONDR predicted disordered regions

(187-219) in cyan. **D**: CD28 AlphaFold structure with the region (52-165) used in different anti-HER2 CARs as HI (114-152) (blue), TM (153-179) (yellow), CS (180-220) (pink). **E**: CD28 AlphaFold structure with topological domains based on UniProt features (UniProt ID: P10747): extracellular in red (19-152), transmembrane in green (153-179), cytoplasmic in blue (180-220).

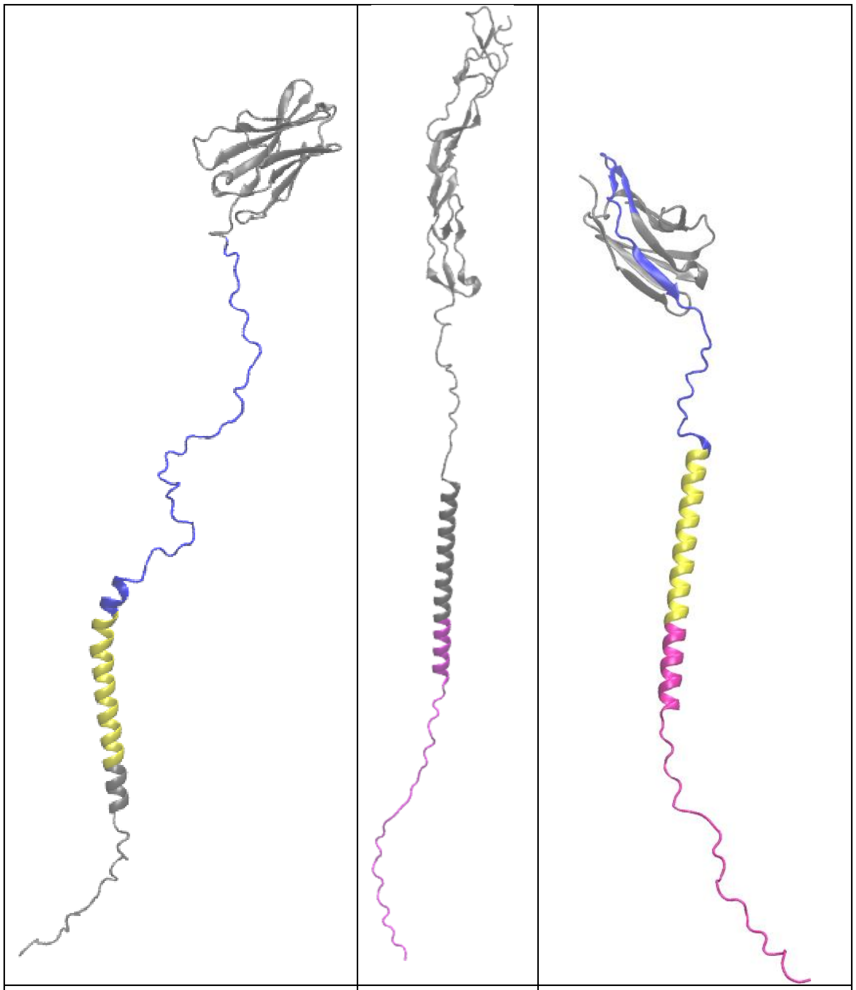

A (CD8α) | B (4-1BB) | C (CD28)

Figure S9. High-resolution structure of transmembrane proteins used in anti-HER2 CAR. **A**: CD8α AlphaFold structure with CD8α regions used in anti-HER2 CAR as HI (138-182) in blue and TM (183-206) in yellow. **B**: 4-1BB AlphaFold structure with 4-1BB region (214-256) used in anti-HER2 CAR as CS in pink. **C**: CD28 AlphaFold structure with the region (52-165) used in different anti-HER2 CARs as HI (114-152) (blue), TM (153-179) (yellow), CS (180-220) (pink).

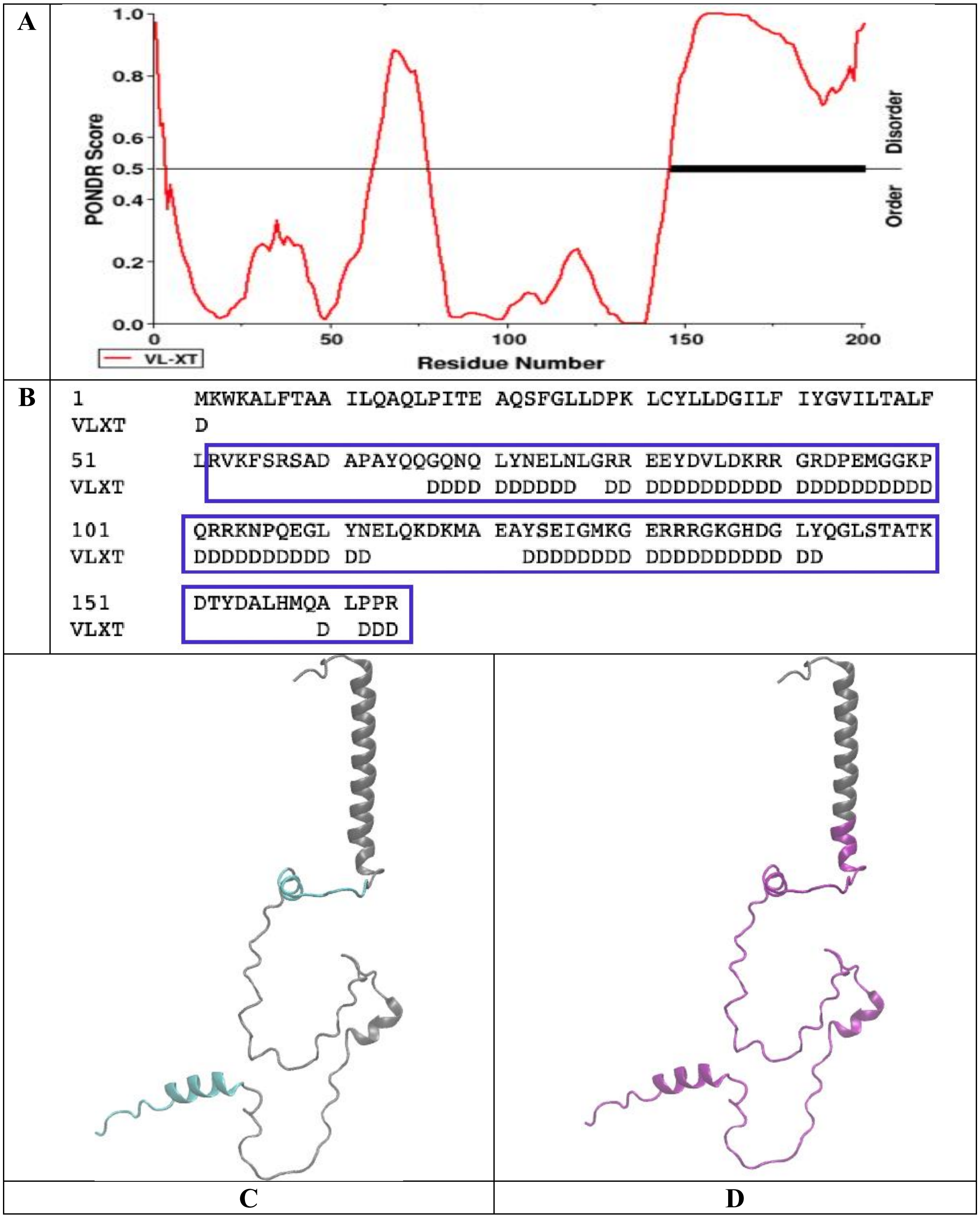

Figure S10. Disordered regions (**A-B**) of CD3ζ predicted by PONDR and its high-resolution structure predicted by AlphaFold (P20963) in the different color schedules (**C-D**). **A**: PONDR prediction diagram of ordered and disordered regions. **B**: PONDR sequence output of ordered and disordered regions with CD3ζ part used as SI in anti-HER2 CAR. **C**: CD3ζ AlphaFold structure with PONDR predicted disordered regions (63-77, 146-201) in cyan. **C**: CD3ζ AlphaFold structure with PONDR predicted disordered regions (67-112, 123-142, 160, 162-164)

in cyan. **D**: CD3ζ AlphaFold structure with the region (52-165) used as SI in anti-HER2 CAR in purple.

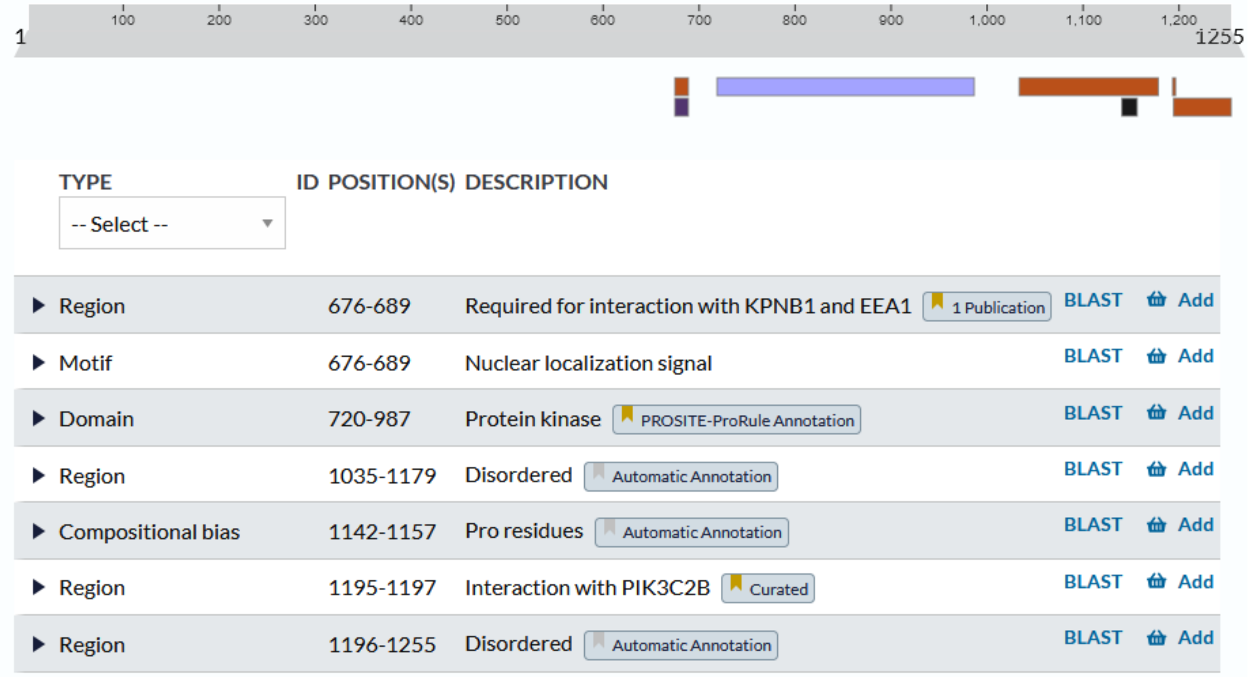

TYPE	ID	POSITION(S)	DESCRIPTION		
-- Select --					
Region		676-689	Required for interaction with KPNB1 and EEA1 1 Publication	BLAST	Add
Motif		676-689	Nuclear localization signal	BLAST	Add
Domain		720-987	Protein kinase PROSITE-ProRule Annotation	BLAST	Add
Region		1035-1179	Disordered Automatic Annotation	BLAST	Add
Compositional bias		1142-1157	Pro residues Automatic Annotation	BLAST	Add
Region		1195-1197	Interaction with PIK3C2B Curated	BLAST	Add
Region		1196-1255	Disordered Automatic Annotation	BLAST	Add

Figure S11. Domains of HER2 (UniProt: P04626).

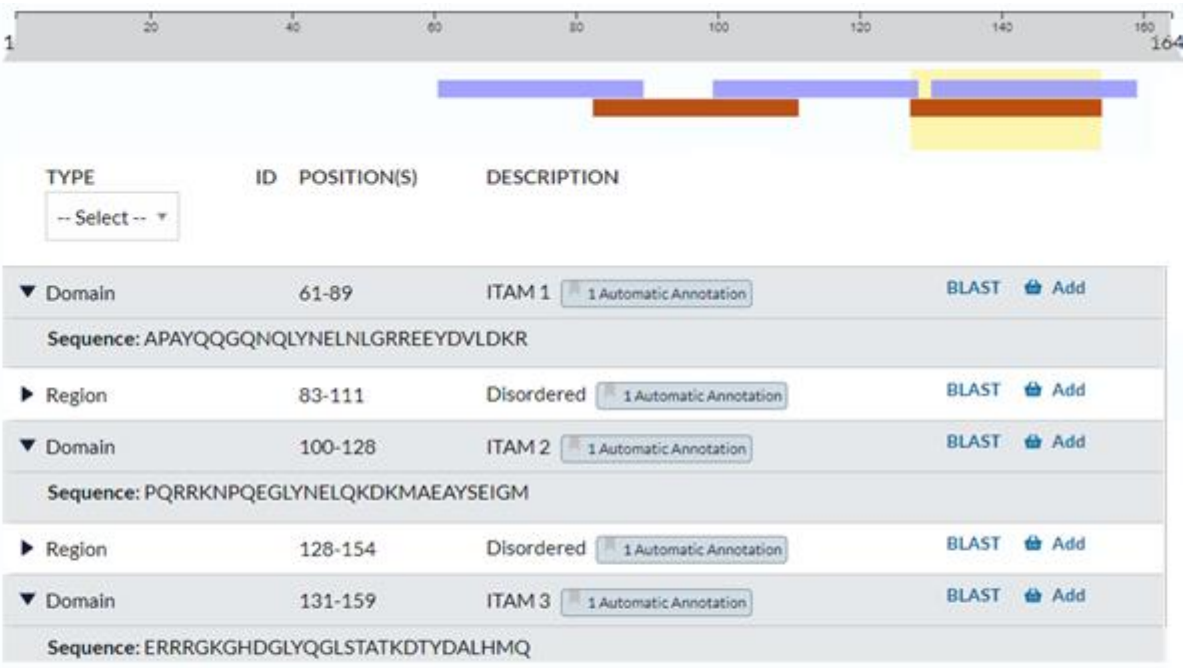

Figure S12. Domains of CD3ζ (UniProt: P20963).

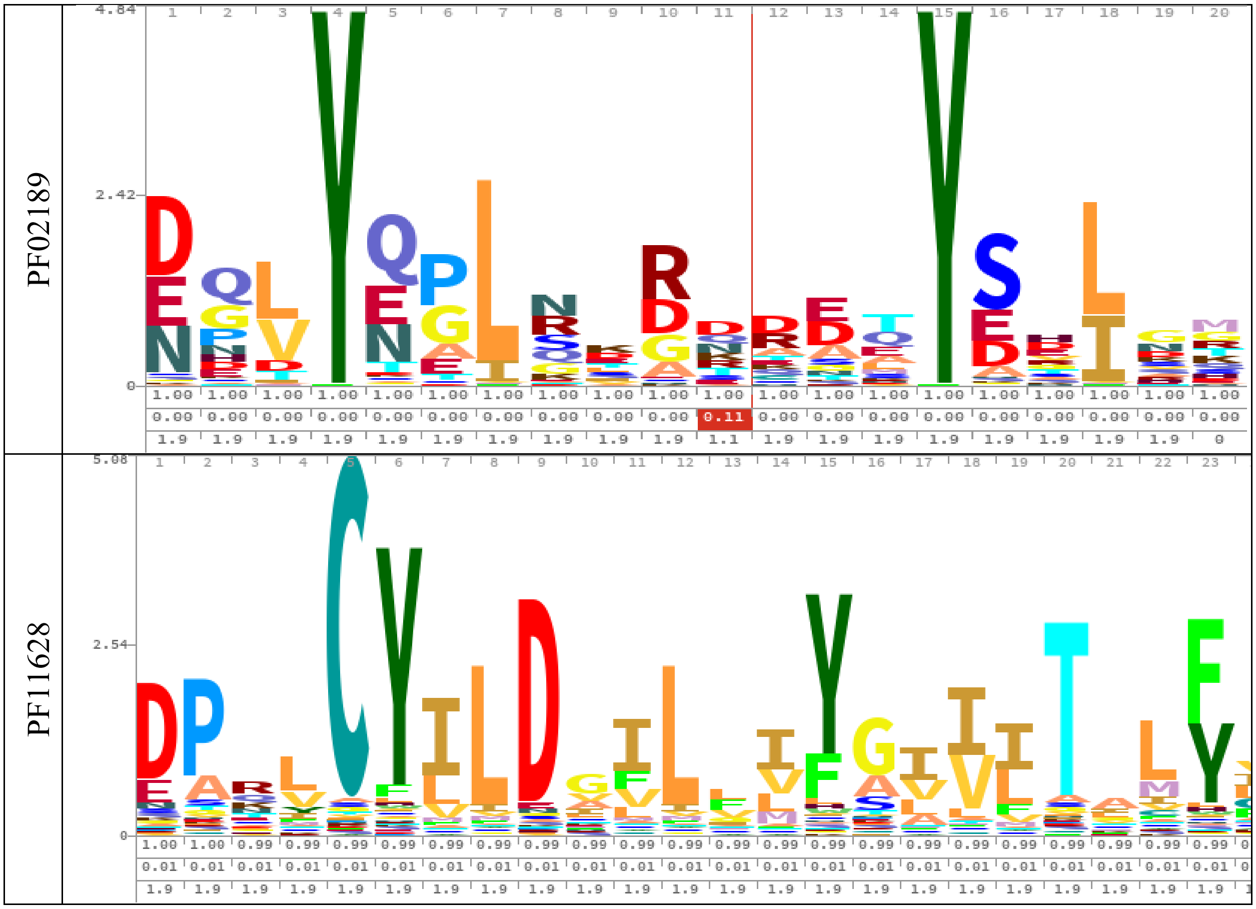

Figure S13. HMM logo for protein family PF02189 and PF11628 of CD3ζ ITAMs.

Family	Sequence	Alignment
PF02189	CD3E_SHEEP/170-189	NPDYEPIRKGQ.RDLYSGLNQ
	Q4V7G0_RAT/69-88	NQLYNELNLGR.REEYDVLDK
	W5PR78_SHEEP/51-70	NPVYNELNVGR.REEYAVLDR
	CD3G_SHEEP/127-146	DQLYQPLKERE.DDQYSHLRK
	F6VBE7_XENTR/144-163	NDLYQPLGQRS.EDTYSHLNS
	Q4V7G0_RAT/139-158	DGLYQGLSTAT.KDTYDALHM
	G3HCU4_CRIGR/175-194	DHTYEGLNIDQ.TATYEDIVT
	CD79A_MOUSE/179-198	ENLYEGLNLDD.CSMYEDISR
	Q4V7G0_RAT/108-128	EGVYNALQKDKMAEAYSEIGM
	I3JR30_ORENI/125-144	EQVYQGLSSAT.RDTYDSLQM
	FCERG_PIG/62-81	DGVYTGLSTRN.QETYETLKH
	F1N9E2_CHICK/152-171	DQLYQPLGERN.DGQYSQLAT
	F7F8B6_MONDO/225-244	DELYQPLRDRD.DNSYSHIGG
	F6X8V5_ORNAN/349-368	DQLYQPLGDRD.DGQYSHIGG
	G1TP12_RABIT/141-160	DQLYQTLQDRD.DAQYSRIGG
	W5PHC2_SHEEP/142-161	DQLYQPLRERN.DAQYSRLGD
	G3IMA2_CRIGR/125-144	EQLYQPLRDRD.DTQYSRLGG
	E9PMT5_HUMAN/73-92	DQVYQPLRDRD.DAQYSHLGG
PF11628	H3A8A0_LATCH/28-58	DPRLCYILDGILFIYGIVITALYLKNKLSAP
	Q4T375_TETNG/12-42	DANVCYILDGILVMFGAILTILFCRLKMNDA
	E2RAM4_CANLF/21-51	EPQLCYILDAILFLYGIVLTLLYCRLKIQVR
	F1QHD5_DANRE/41-71	DPTYCYILDVILLVYSIIFTALYFREKFLKE
	W5MU19_LEPOC/27-57	DPRLCYILDGVLLIYGIIITALYFKDKFSKP
	K7GS60_PIG/28-58	DPKLCYLLDGILFIYGVIITALFLREKFSRS
	G1KJ09_ANOCA/28-58	DPRLCYILDGILLIYAIVITACFVKTKLSKG
	G3UV56_MELGA/29-59	DPRLCYLLDGFLFIYAVIITALFVKAKLSQS
	H3CM88_TETNG/27-57	DPRVCYILDIFLGVYGLVITGMFIREKFFRS
	I3JR30_ORENI/27-57	DPQLCYILDGFLGIYGLIITGMFIKEKFFKS

This alignment is coloured according to the ClustalX colouring scheme:

- Glycine (G)
- Proline (P)
- Small or hydrophobic (C,A,V,L,I,M,F,W)
- Hydroxyl or amine amino acids (S,T,N,Q)
- Charged amino-acids (D,E,R,K)
- Histidine or tyrosine (H,Y)

For UniProt-based alignments, we also add some additional mark-up to the alignments where appropriate. Active site information is shown as follows:

- Active site (residue annotated in SwissProt as an active site)
- Predicted active site (residue aligns in a Pfam alignment with a SwissProt active site)
- Predicted active site (residue annotated in SwissProt as a potential active site)

Some UniProt sequences can be mapped to protein structures, in which case we also show the secondary structure definition. These lines are shown below the sequence to which they apply and are marked (SS). The meaning of each of the symbols is as follows:

C Random coil
H Alpha-helix
G 3(10) helix
I Pi-helix
E Hydrogen bonded beta-strand (extended strand)
B Residue in isolated beta-bridge
T H-bonded turn (3-turn, 4-turn, or 5-turn)
S Bend (five-residue bend centered at residue i)

Figure S14. Seed sequences and protein family PF11628 of CD3ζ ITAMs.

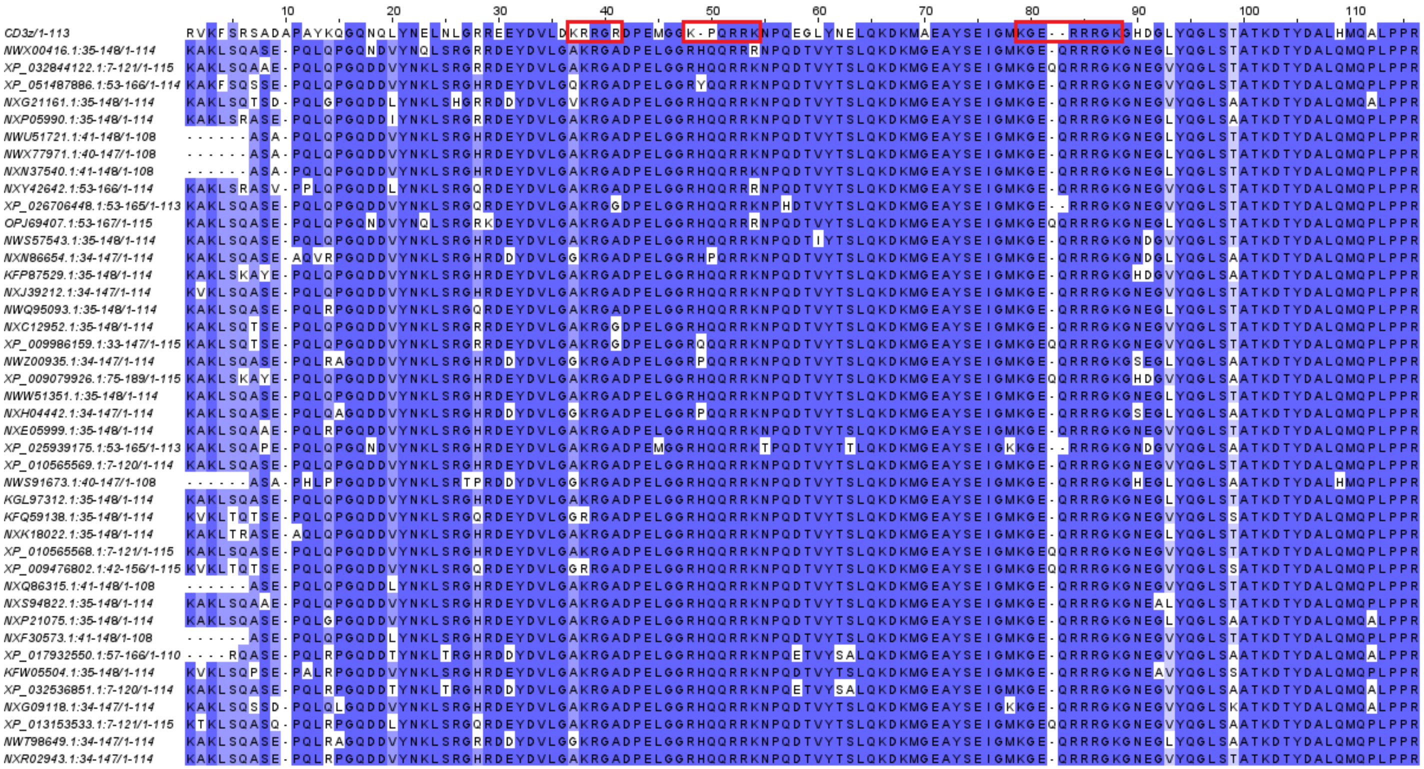

Figure S15. Multiple sequence alignment of CD3ζ (359-470) with 48 different non-mammalian organisms. PBRs are in red.

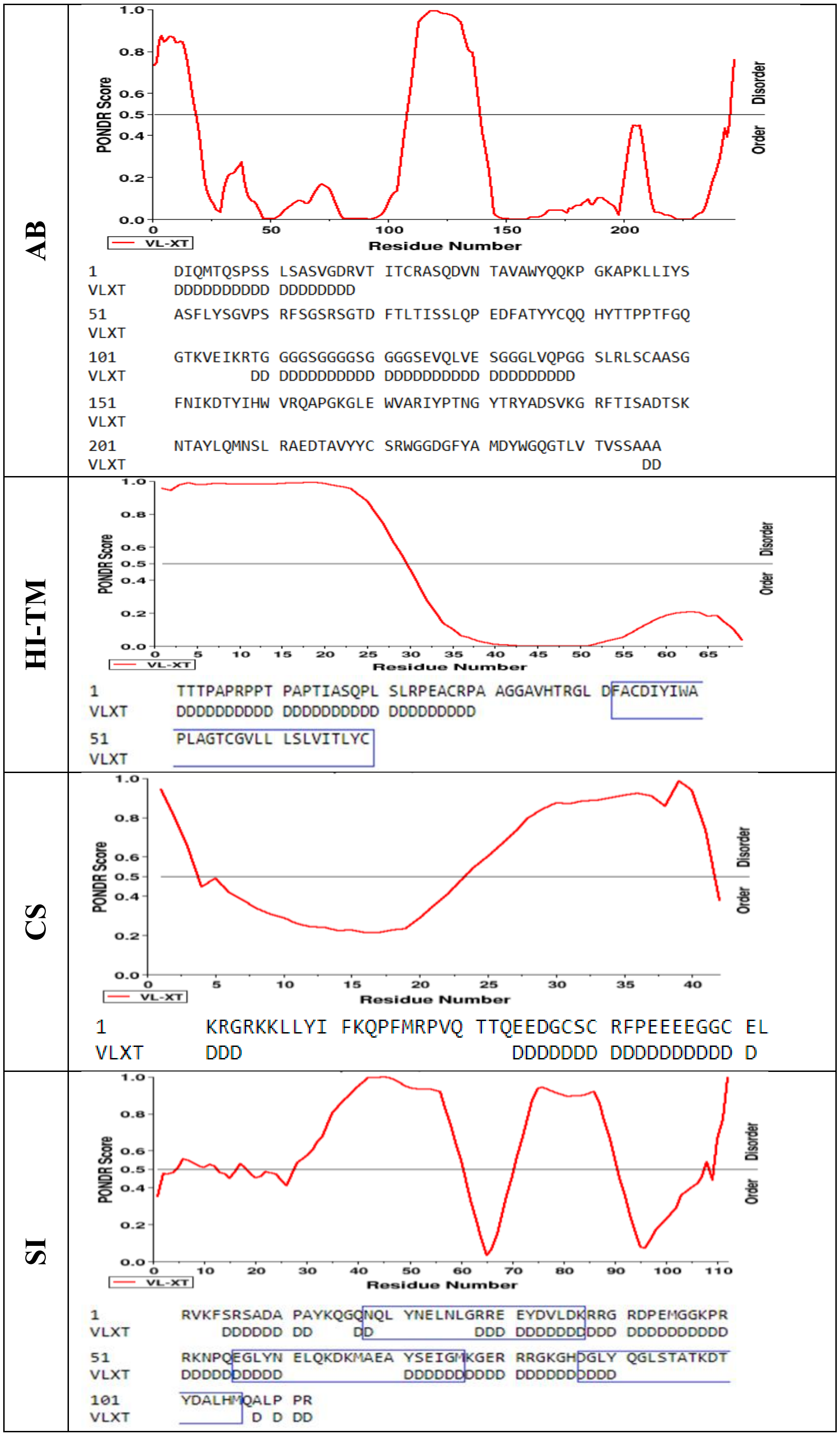

Figure S16. Prediction of Natural Disordered Regions for AB, HI-TM, CS, SI by PONDR. VLXT refers to the merger of three predictors, one trained on ***V***ariously characterized ***L***ong disordered regions and two trained on ***X***-ray characterized ***T***erminal disordered regions. Blue box of HI-TM: the transmembrane helical part; Blue box of SI: three ITAMs.

Top 10 threading templates used by I-TASSER

(I-TASSER modeling starts from the structure templates identified by LOMETS from the PDB library. LOMETS is a meta-server threading approach containing multiple threading programs, where each threading program can generate tens of thousands of template alignments. I-TASSER only uses the templates of the highest significance in the threading alignments, the significance of which are measured by the Z-score, i.e. the difference between the raw and average scores in the unit of standard deviation. The templates in this section are the 10 best templates selected from the LOMETS threading programs. Usually, one template of the highest Z-score is selected from each threading program, where the threading programs are sorted by the average performance in the large-scale benchmark test experiments.)

Rank	PDB Hit	Iden1	Iden2	Cov	Norm. Z-score	Download Align.	20 / 40 / 60
						Sec.Str	CCCCCCCCCCCCCCCCCCCCCCCCCCCCCCCCCCCCCCCCCCCCSSSSSHHHHHHHHHHHHHHHHSSSC
						Seq	TTTPAPRPPTPAPTIASQPLSLRPEACRPAAGGAVHTRGLDFACDIYIWAPLAGTCGVLLLSLVITLYC
1	7vu5	0.17	0.13	0.35	1.29	Download	--SKPFWVLVVVGGVLAFYSLLVTVA-
2	3gauA	0.16	0.23	0.81	1.00	Download	--ELPPRRNTEILTGSWSDQTYPEGTQAIYKPGYRSLGNVIMVCRKGEWVALNPLR-----------KC
3	7vu5	0.15	0.13	0.39	1.06	Download	--GPSKPFWVLVVVGGVLAFYSLLVTVAF
4	7vu5	0.15	0.13	0.39	1.12	Download	--GPSKPFWVLVVVGGVLAFYSLLVTVAI
5	6lumA	0.14	0.29	0.84	0.48	Download	-----GRPAPVMEREHDRPAALDHPRAPRKPRGIPYFEKYAWLFM-----RFSGIALVFLALGHLFIM-
6	7t62A	0.09	0.25	0.93	0.81	Download	AVAPPARPPRPPYPPRRDGSGGK----GGGGSARYNQGRSRSGGASIGFHTQTILILSLSALALLGPR-
7	1nayA	0.15	0.17	0.78	0.88	Download	PGPPGPPGPPGPPGPPGPPGPPGPPGSGYIPEAPRDGQAYVRKDG--EWVLLSTFL-------------
8	2wfk	0.23	0.30	0.19	0.77	Download	---------------------------------------KKAY-YLYVWIPAV----------------
9	7m6eA	0.25	0.22	0.94	0.66	Download	VITPGTNTSNQVAVLQDVNCTEVPNVFQTRAGGAEHVNN-SYECDIPIG---AGICASYQQSIIAYTMS
10	4cbfB	0.06	0.23	1.00	0.96	Download	ALTPHSGMGLETRAETWMSSEGAWKHAQRVESWILRNPGFALLAGFMAYMIGQTGIQRTVFFVLMMLVA

(a) All the residues are colored in black; however, those residues in template which are identical to the residue in the query sequence are highlighted in color. Coloring scheme is based on the property of amino acids, where polar are brightly coloured while non-polar residues are colored in dark shade. (more about the colors used)

(b) Rank of templates represents the top ten threading templates used by I-TASSER.

(c) Ident1 is the percentage sequence identity of the templates in the threading aligned region with the query sequence.

(d) Ident2 is the percentage sequence identity of the whole template chains with query sequence.

(e) Cov represents the coverage of the threading alignment and is equal to the number of aligned residues divided by the length of query protein.

(f) Norm. Z-score is the normalized Z-score of the threading alignments. Alignment with a Normalized Z-score >1 mean a good alignment and vice versa.

(g) Download Align. provides the 3D structure of the aligned regions of the threading templates.

(h) The top 10 alignments reported above (in order of their ranking) are from the following threading programs:

1: HHSEARCH 2: Neff-PPAS 3: HHSEARCH2 4: HHSEARCH I 5: FFAS-3D 6: SPARKS-X 7: SP3 8: HHSEARCH 9: wdPPAS 10: Neff-PPAS

Figure S17. The output from the homology modeling of HI and TM using I-TASSER webserver.

Top 10 threading templates used by I-TASSER

(I-TASSER modeling starts from the structure templates identified by LOMETS from the PDB library. LOMETS is a meta-server threading approach containing multiple threading programs, where each threading program can generate tens of thousands of template alignments. I-TASSER only uses the templates of the highest significance in the threading alignments, the significance of which are measured by the Z-score, i.e. the difference between the raw and average scores in the unit of standard deviation. The templates in this section are the 10 best templates selected from the LOMETS threading programs. Usually, one template of the highest Z-score is selected from each threading program, where the threading programs are sorted by the average performance in the large-scale benchmark test experiments.)

Rank	PDB Hit	Iden1	Iden2	Cov	Norm. Z-score	Download Align.		20 40
							Sec.Str	CHHHHHHHHHHHHHHCCCCHHHHHHCCCCCCCCCHHCCCCCC
							Seq	KRGRKKLLYIFKQPFMRPVQTTQEEDGCSCRFPEEEEGGCEL
1	6itcB	0.17	0.21	1.00	1.11	Download		AKKTAIAIAVALAGFATVASYAQYEDGCSGELERDSPHSYHS
2	6itcB	0.23	0.21	0.93	1.01	Download		AKKTAIAIAVALAGFATVASYAQYEDGCSGELPHSYHSG---
3	6c0fI	0.14	0.21	1.00	1.13	Download		KLKRQEIFADIKHEKNKERHTMRRKRAKEERENPELREQRLK
4	1jj2A	0.14	0.14	1.00	1.01	Download		KATKKRLAKLDNQNSRVPAWVMLKTDERNHKRRHWRRNDTDE
5	6exnD	0.10	0.12	0.98	1.06	Download		NLRKKNLEMSQRAEMINRSKHAQQEKAVTTDDLDNLVDQVF-
6	5w9fA	0.17	0.21	0.95	1.05	Download		QETRKKCTEMKKKFKNCEVRCDESNHCVEVRCSDTKYTLC--
7	7lveA	0.15	0.14	0.98	1.04	Download		MQDPQQQYHRCQRRCQIQEQSPERQRQCQCERQYKEQQGRE-
8	3hd6A	0.23	0.38	0.74	0.26	Download		------VGLILRLPFWGQPSDENCEDAVYWEMPEGNS-----
9	7msjA	0.19	0.26	0.86	0.83	Download		------GVCDPDMGLLTWQECSSWKDTCRCYFCENQDGSCLQ
10	1lu0A	0.17	0.17	0.69	0.88	Download		------------RVCPRILLECKKDSDCLAECVCLEHGYCG-

(a) All the residues are colored in black; however, those residues in template which are identical to the residue in the query sequence are highlighted in color. Coloring scheme is based on the property of amino acids, where polar are brightly coloured while non-polar residues are colored in dark shade. (more about the colors used)

(b) Rank of templates represents the top ten threading templates used by I-TASSER.

(c) Ident1 is the percentage sequence identity of the templates in the threading aligned region with the query sequence.

(d) Ident2 is the percentage sequence identity of the whole template chains with query sequence.

(e) Cov represents the coverage of the threading alignment and is equal to the number of aligned residues divided by the length of query protein.

(f) Norm. Z-score is the normalized Z-score of the threading alignments. Alignment with a Normalized Z-score >1 mean a good alignment and vice versa.

(g) Download Align. provides the 3D structure of the aligned regions of the threading templates.

(h) The top 10 alignments reported above (in order of their ranking) are from the following threading programs:

1: SP3 2: wdPPAS 3: Neff-PPAS 4: SP3 5: Neff-PPAS 6: Neff-PPAS 7: Neff-PPAS 8: FFAS-3D 9: SPARKS-X 10: SP3

Figure S18. The output from the homology modeling of CS using I-TASSER webserver.

Top 10 threading templates used by I-TASSER

(I-TASSER modeling starts from the structure templates identified by LOMETS from the PDB library. LOMETS is a meta-server threading approach containing multiple threading programs, where each threading program can generate tens of thousands of template alignments. I-TASSER only uses the templates of the highest significance in the threading alignments, the significance of which are measured by the Z-score, i.e. the difference between the raw and average scores in the unit of standard deviation. The templates in this section are the 10 best templates selected from the LOMETS threading programs. Usually, one template of the highest Z-score is selected from each threading program, where the threading programs are sorted by the average performance in the large-scale benchmark test experiments.)

Rank	PDB Hit	Iden1	Iden2	Cov	Norm. Z-score	Download Align.	20 / 40 / 60 / 80 / 100
						Sec.Str	CCCCCCCCCCHHHHCCCHHHHHHHCCCCCHHHHHHHCCCCCCHHHCCCHHCCCCCHHHHHHHHCCCCHHHHHHHCCCCHHHCCCCCCHHHHCCCCCCCCHHHHHHCCCCCCC
						Seq	RVKFSRSADAPAYKQGQNQLYNELNLGRREEYDVLDKRRGRDPEMGGKPRRKNPQEGLYNELQKDKMAEAYSEIGMKGERRRGKGHDGLYQGLSTATKDTYDALHMQALPPR
1	2k4f	0.32	0.15	0.20	1.03	Download	----------------PNPDYEPIRKGQRDLYSGLNQR--
2	6x0vF	0.16	0.18	0.85	1.02	Download	-----------RIHHDVNELLSLLRVHAEVYIDLLQKNRTPYVT-----TTVSAHSAKVKIAEFSRTPEDFLKKYDELKSKNTRNLDPLVYLLSKLTEDKETLQYLQQNAK-
3	2k4f	0.20	0.15	0.36	1.50	Download	KAKAKPVRNKERPPPVPNPDYEPIRKGQRDLYSGLNQRA-V---
4	6exnD	0.10	0.19	0.87	1.02	Download	KTAVESIDETLQRLVREKEMEQNEKGIKEQADDKMDLLEKRLAKIQQEQEDDE------------ELENLRKKNLEMSQRAEMINRSKHAQQEKAVTTDDLDNLVDQVF---
5	3oa7A	0.11	0.25	0.84	0.37	Download	--------------EEHEDILNKLLDPELAQSERTEALQQLRVNYGSFVSEYNDLTKSHNTLSKELDNLRSRFGNLEGNTSERITIKNILQSRPDISAEECNFL-VEQI---
6	7wb4I	0.11	0.35	1.00	0.65	Download	EQAMDSALPAEDDNAIREHLCIRAYLESHEAFNEWFKHINSPPQKPTLVGQASFTEKVAHEHKEKKYEMDFGIWKGHLDALTSVDGGWMVDVREDTEEDPERSHQMVLLRRL
7	2ctwA	0.19	0.16	0.87	0.68	Download	----GSSGSSGRQRS--------LSTSGESLYHVLGLDKNATSDDIKKSYRKNPDNPEAADKFK-EINNAHAILTDATKRNIKYGSLGLYVAEQFGEENVNTYFVSG--PSS
8	7cg4	0.31	0.13	0.12	0.76	Download	--VRNKLVAAYQEVM-------------------------------------
9	2mphA	0.18	0.16	0.85	0.52	Download	-----MAAAVPQRAWTVEQLRSE-QLPKKDIIKFL--QEHGSDSFLAEHKLLGNIKNVAKTANKDHLVTAYNHL----ETKRFKGTE----SISKVSEQV-KNVKLNEDKPK
10	5hmoA	0.14	0.22	0.99	0.96	Download	-QKQLRGQIARRVYRQLLAEKRAEEEKRKREEEEKRKREEEEEREREREREAELRAQQEEAARKQRELEALQQESQRAAELSKQKENKQVEEILRLEKEIEDLQRMKERQEL

(a) All the residues are colored in black; however, those residues in template which are identical to the residue in the query sequence are highlighted in color. Coloring scheme is based on the property of amino acids, where polar are brightly coloured while non-polar residues are colored in dark shade. (more about the colors used)

(b) Rank of templates represents the top ten threading templates used by I-TASSER.

(c) Ident1 is the percentage sequence identity of the templates in the threading aligned region with the query sequence.

(d) Ident2 is the percentage sequence identity of the whole template chains with query sequence.

(e) Cov represents the coverage of the threading alignment and is equal to the number of aligned residues divided by the length of query protein.

(f) Norm. Z-score is the normalized Z-score of the threading alignments. Alignment with a Normalized Z-score >1 mean a good alignment and vice versa.

(g) Download Align. provides the 3D structure of the aligned regions of the threading templates.

(h) The top 10 alignments reported above (in order of their ranking) are from the following threading programs:

1: HHSEARCH 2: Neff-PPAS 3: HHSEARCH2 4: Neff-PPAS 5: FFAS-3D 6: SPARKS-X 7: SP3 8: HHSEARCH 9: wdPPAS 10: Neff-PPAS

Figure S19. The output from the homology modeling of SI using I-TASSER webserver.

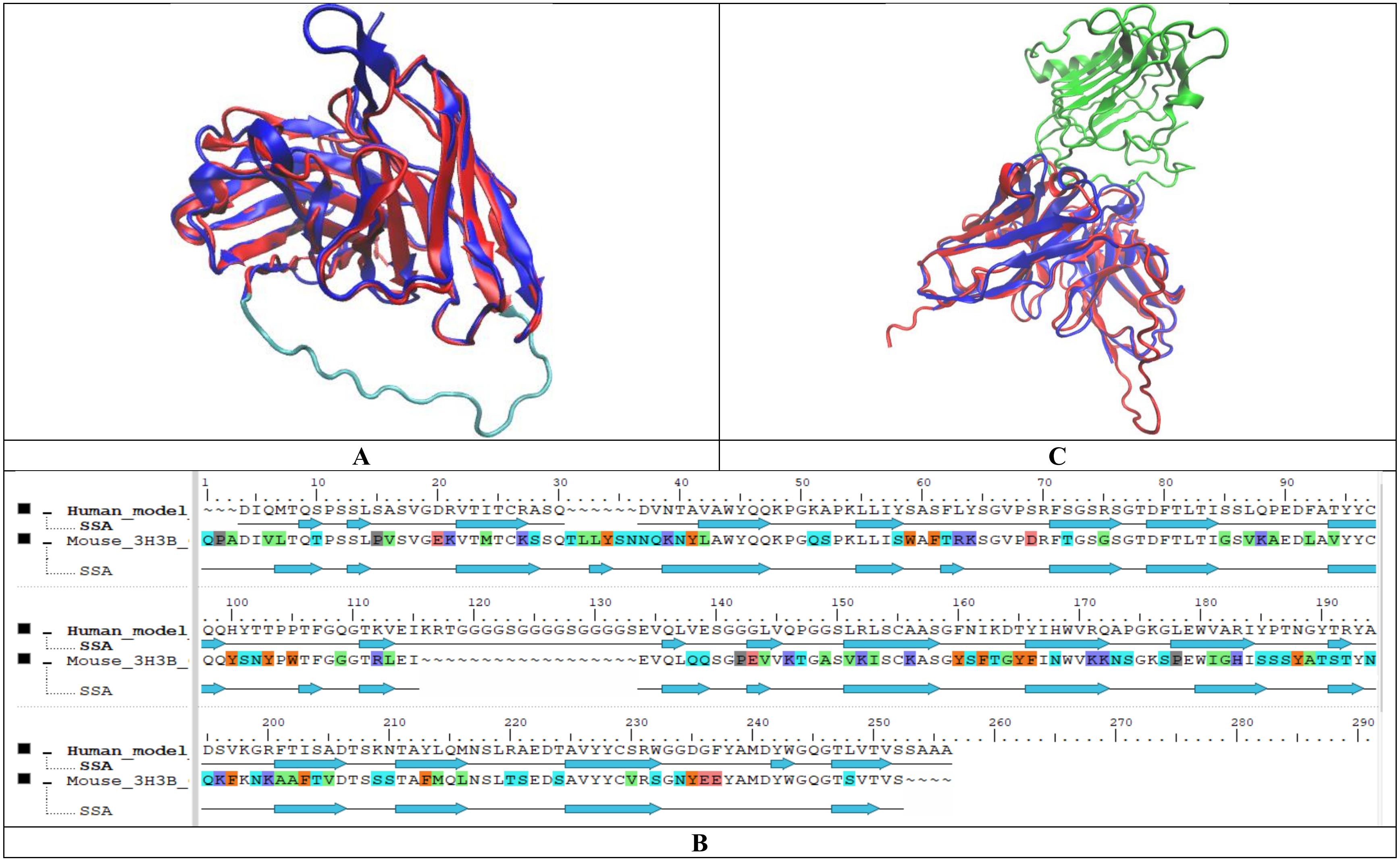

Figure S20. Homology model of human anti-HER2 CAR AB (red) in complex with human AG (green). A: Overlapping of human anti-HER2 CAR AB homology model (red) with the mouse Anti-ErbB2 antibody crystal structure (PDB ID: 3H3B, blue) including the filled linker (in cyan). B: Sequence alignment between human anti-HER2 CAR AB and mouse Anti-ErbB2 in crystal structure (PDB ID: 3H3B), C: the human AG (green) from the crystal structure (3H3B) was included.

	Model 1	Model 2	Model 3
**A**	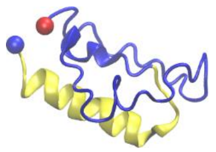	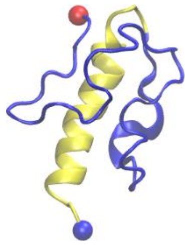	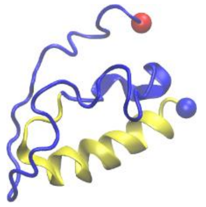
	C-Score=-1.89,<br>TM-score=0.49±0.15<br>Exp. RMSD= 6.0±3.7	C-Score = -3.82	C-Score =-3.88
**B**	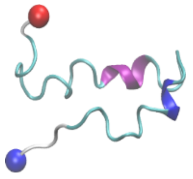	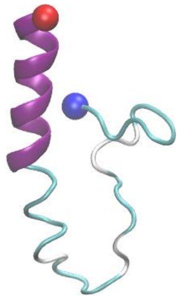	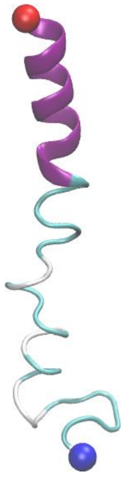
	C-Score=-2.10,<br>TM-score= 0.46±0.15<br>Exp. RMSD= 6.5±3.9	C-Score = -3.62	C-Score = -2.82
**C**	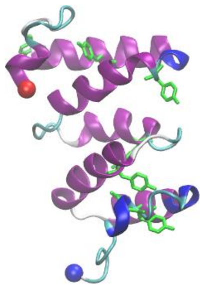	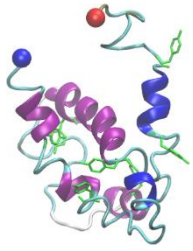	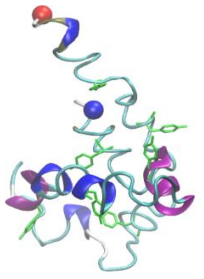
	C-score = -4.02,<br>TM-score = 0.28±0.09<br>Exp. RMSD = 13.5±4.0	C-Score = -4.44	C-Score = -4.01

Figure S21. Top three homology models with assessment scores (C-score, TM-Score, Exp. RMSD) for the anti-HER2 CAR structure domains built from I-TASSER webserver. **A**: HI (blue) and TM (yellow). **B**: CS. **C**: SI. N-terminal in red, C-terminal in blue.

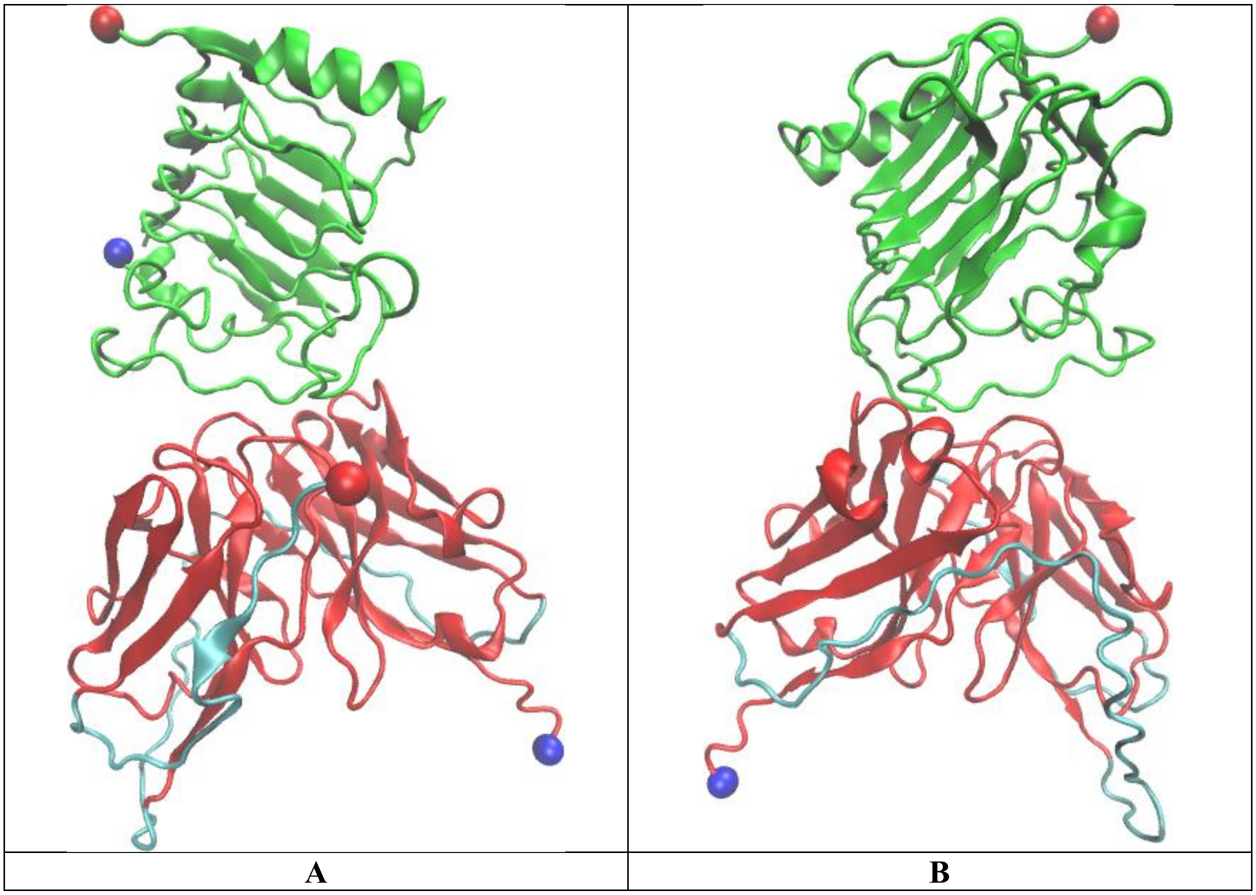

Figure S22. The complex of AG (in green) and AB (in red) with disordered linker region (109-139) predicted by PONDR (in cyan) in two perspectives. N-terminal in red, C-terminal in blue.

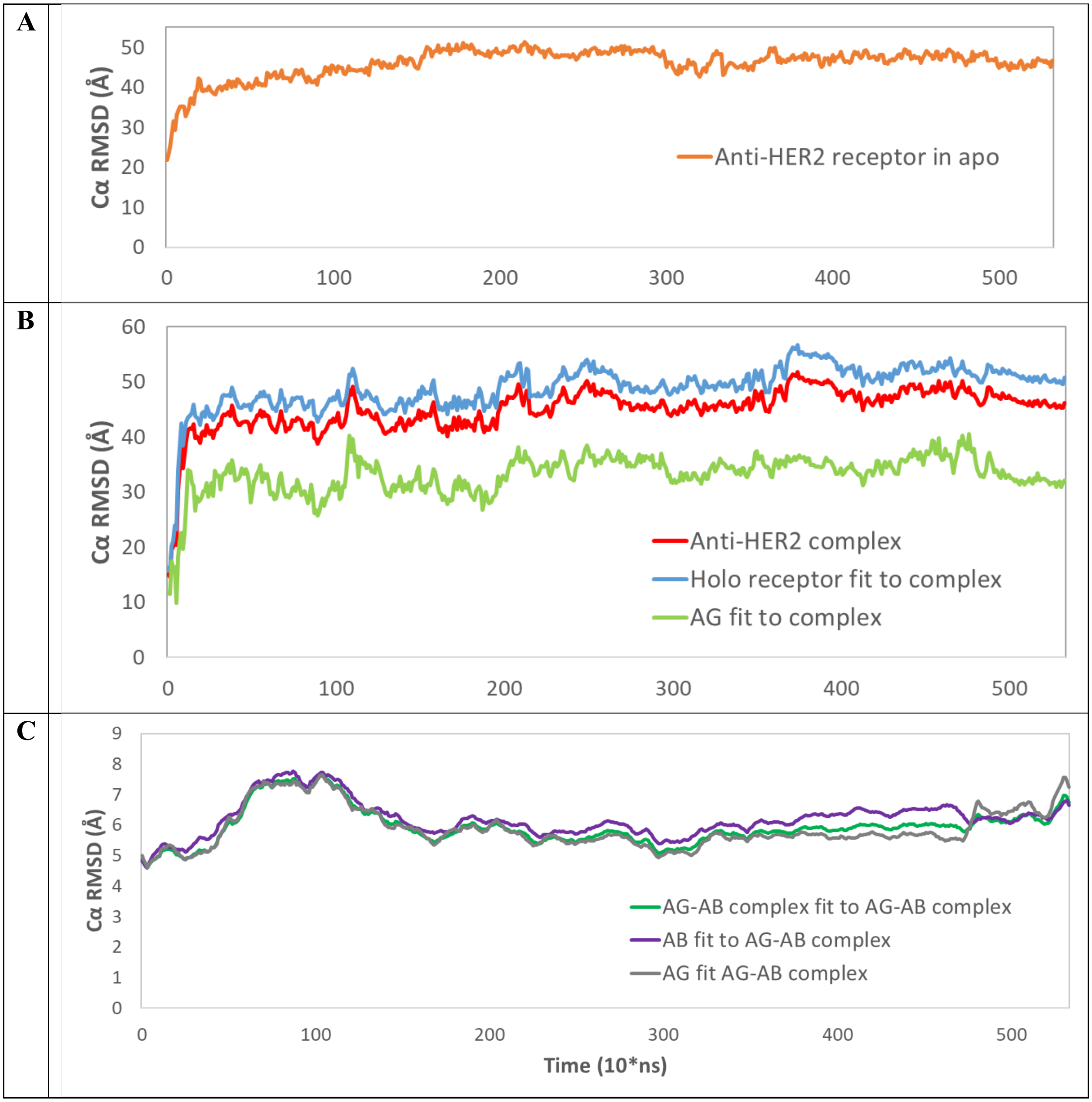

Figure S23. The development of the Cα-RMSD of the receptor in apo-form and holo-form **for all three combined trajectories**. **A**: Cα-RMSD of the anti-HER2 CAR in the apo-form in orange and in the holo-form complex in red with AG by fitting to its full initial structure (receptor for apo-form and full complex for holo-form). **B:** Decomposition of the holo-form: Cα-RMSD of the complex in red, ligand of the complex in green, and the receptor of the complex in blue when fitting to the holo-form complex. **C**: The AG and AB complex: Cα-RMSD of the AG-AB complex in green, AB fit to AG-AB complex in purple, AG fit to AG-AB complex in grey. The summary over the converged regions is shown in **Table S9-10.**

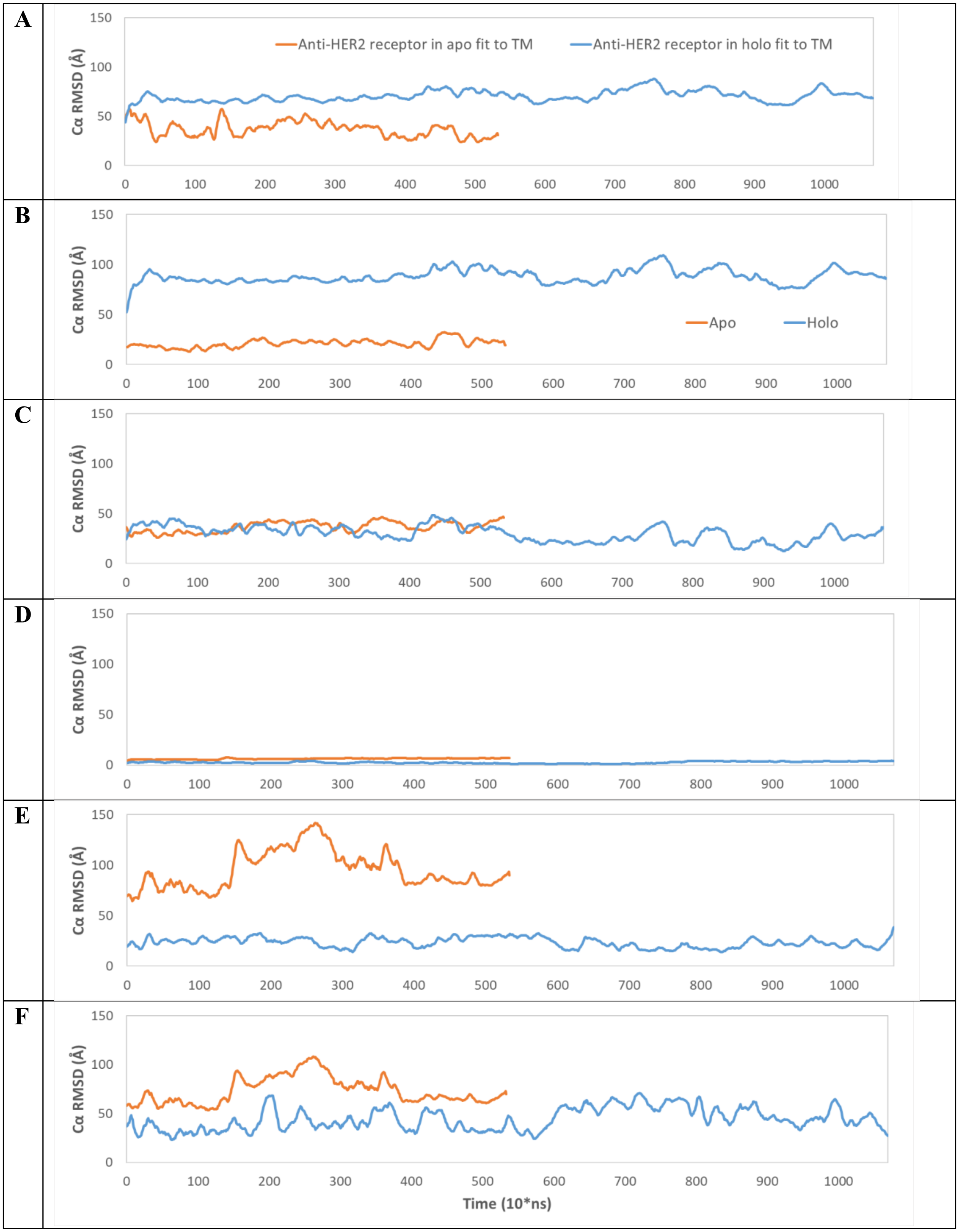

Figure S24. The development of the Cα-RMSD of the receptor parts in apo-form (orange) and holo-form (blue) by all fitting to TM of the anti-HER2 CAR for the first trajectory. **A**: anti-HER2 CAR. **B**: AB. **C:** HI **D**: TM. **E**: CS. **F**: SI.

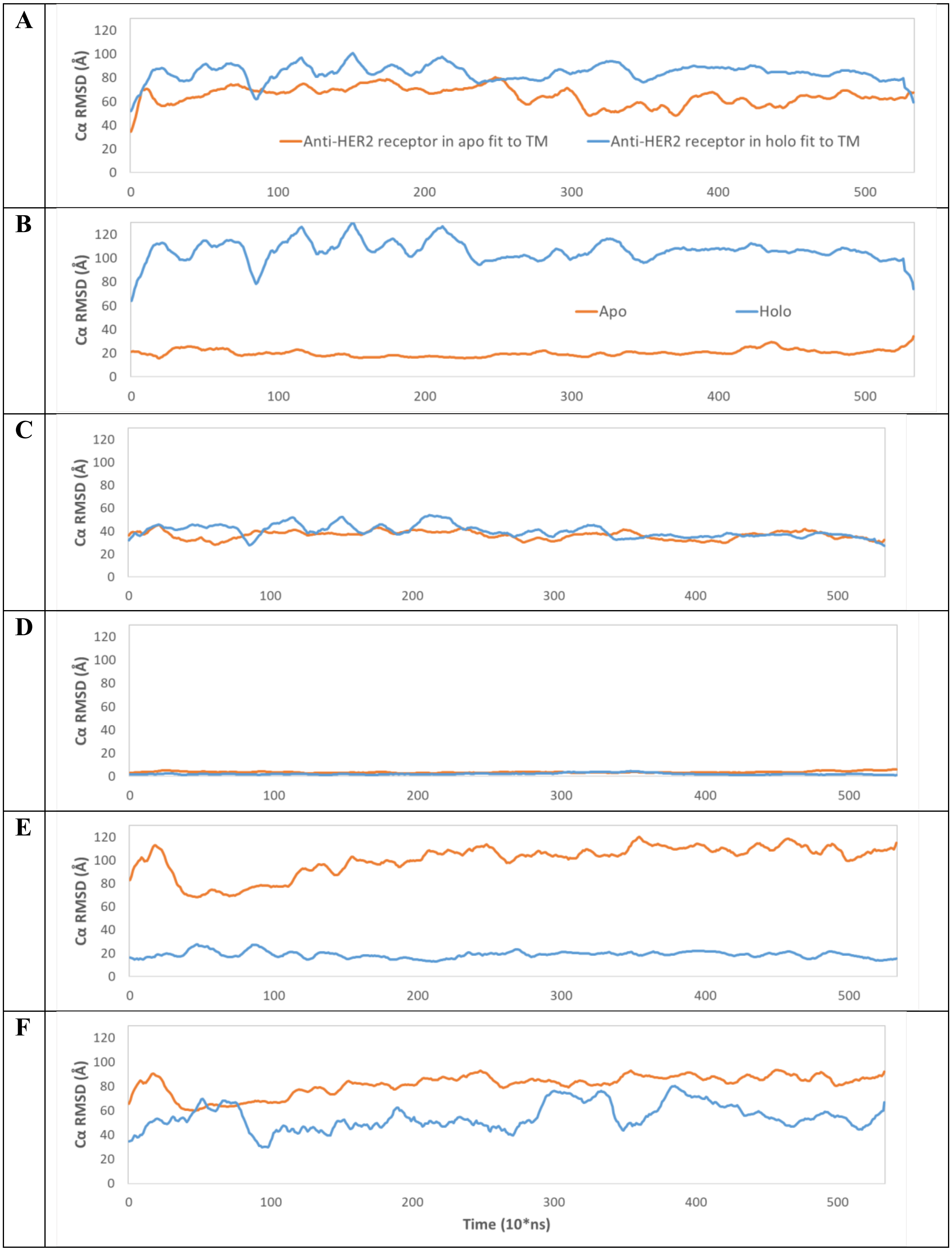

Figure S25. The development of the Cα-RMSD of the receptor parts in apo-form (orange) and holo-form (blue) by all fitting to TM of the anti-HER2 CAR for the second trajectory. A: anti-HER2 CAR. B: AB. C: HI D: TM E: CS. F: SI.

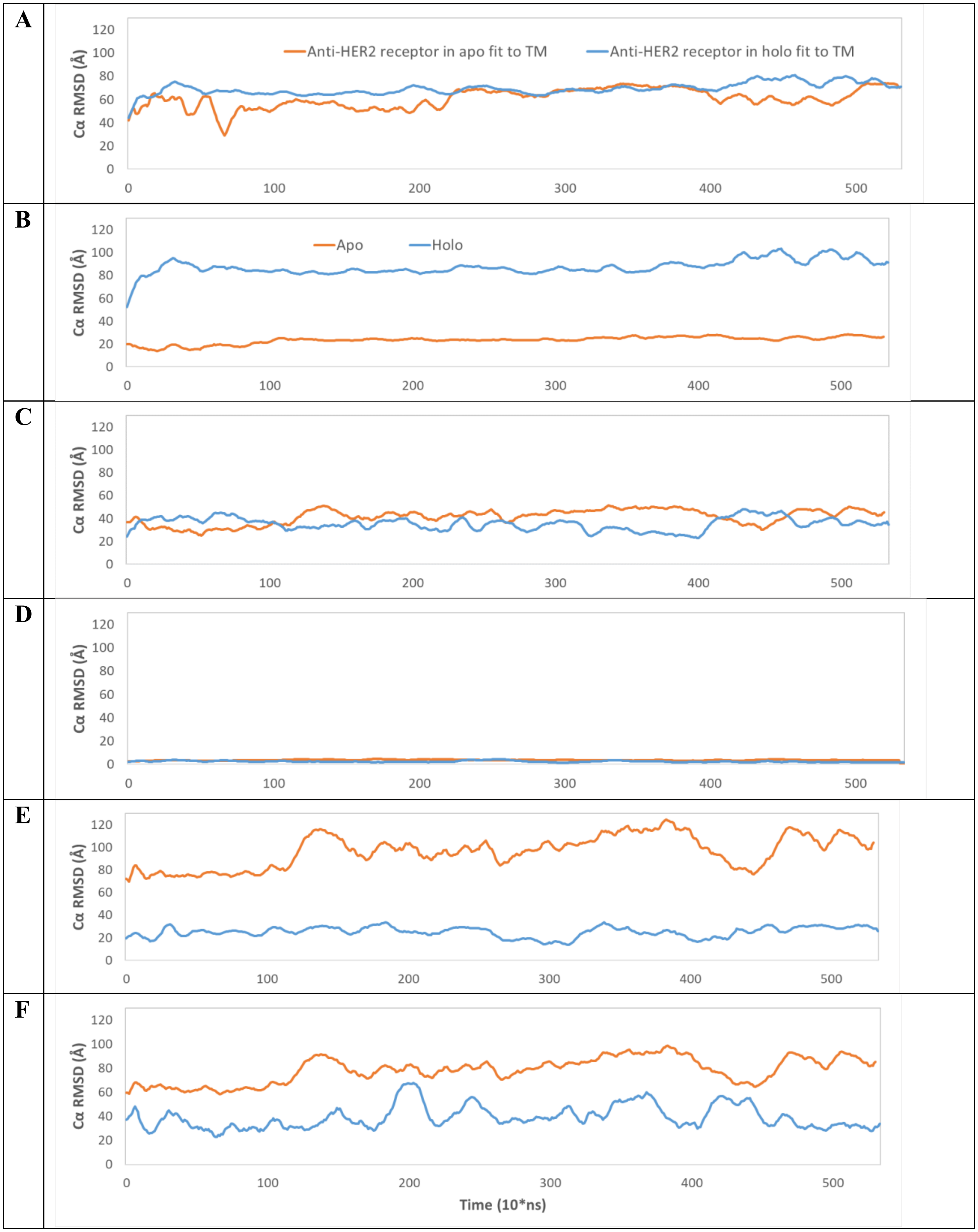

Figure S26. The development of the Cα-RMSD of the receptor parts in apo-form (orange) and holo-form (blue) by all fitting to TM of the anti-HER2 CAR for the third trajectory. A: anti-HER2 CAR. B: AB. C: HI D: TM **E**: CS. **F**: SI.

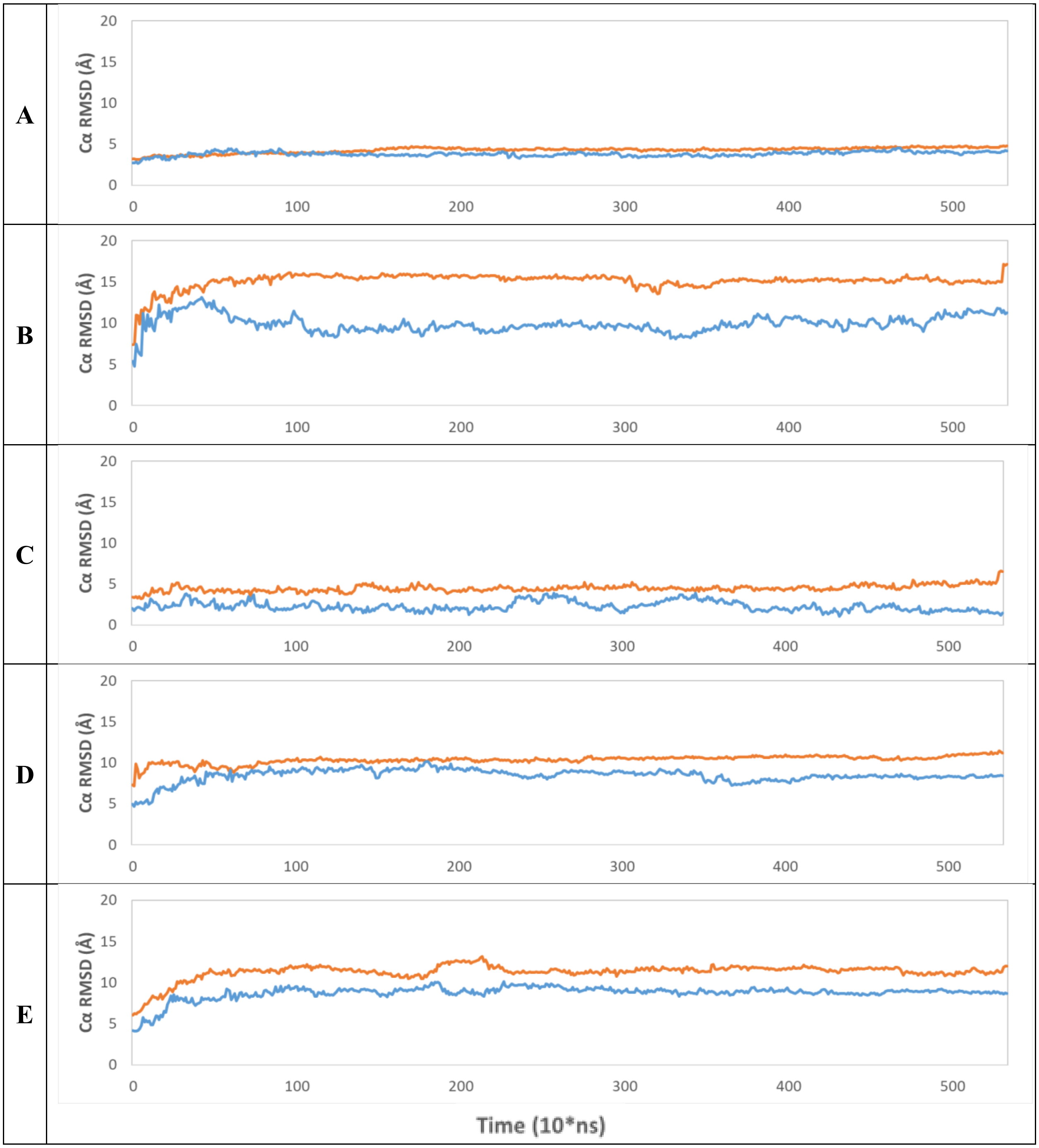

Figure S27. The development of the Cα-RMSD of the receptor domains in apo-form (orange) and holo-form (blue) by fitting to the domain self for all three combined trajectories. **A:** AB. **B**: HI. **C**: TM. **D**: CS. **E**: SI.

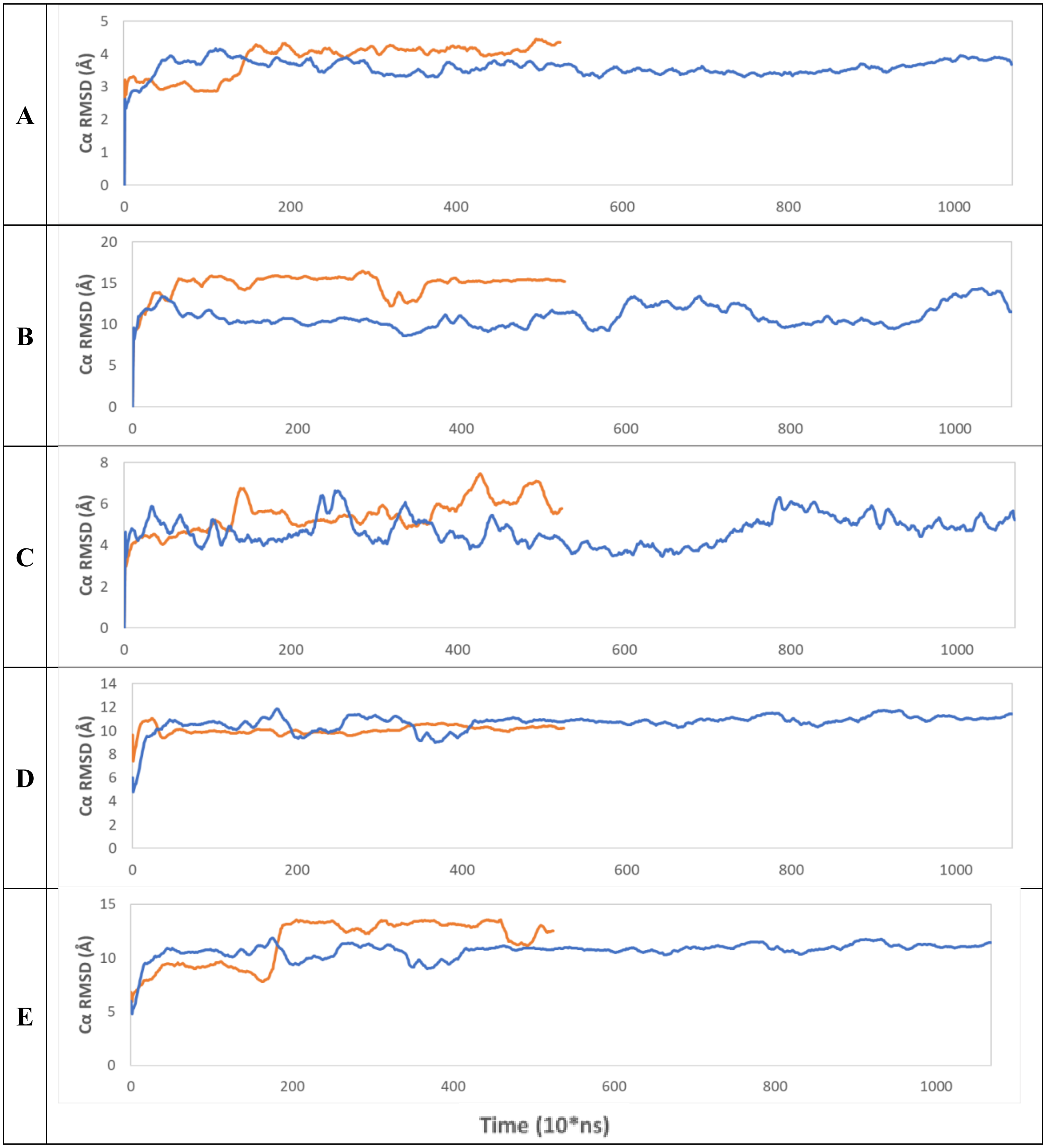

Figure S28. The development of the Cα-RMSD of the receptor domains in apo-form (orange) and holo-form (blue) by fitting to the domain self for the first trajectory. **A:** AB. **B**: HI. **C**: TM. **D**: CS. **E**: SI.

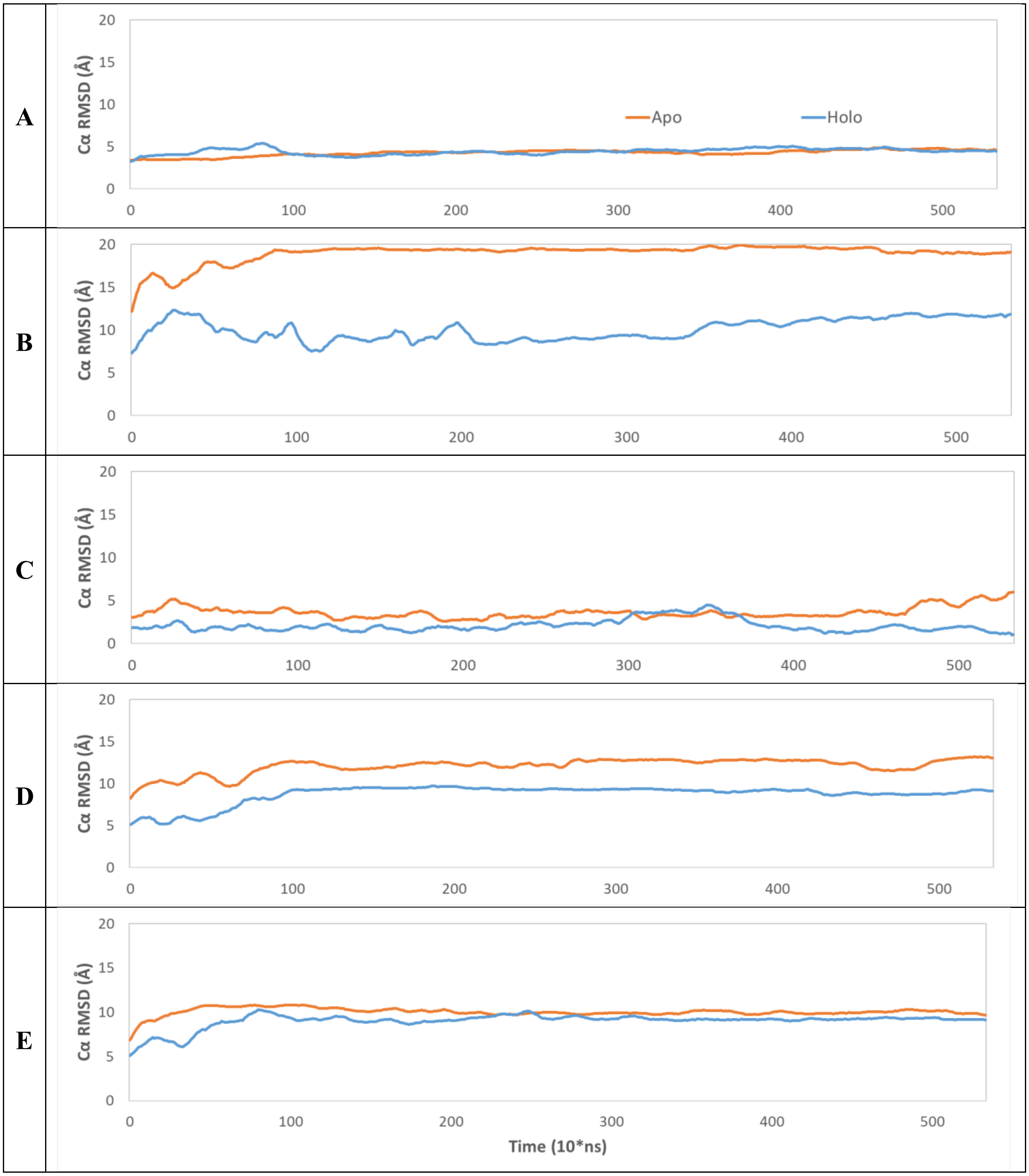

Figure S29. The development of the Cα-RMSD of the receptor domains in apo-form (orange) and holo-form (blue) by fitting to the domain self for the second trajectory. **A:** AB. **B**: HI. **C**: TM. **D**: CS. **E**: SI.

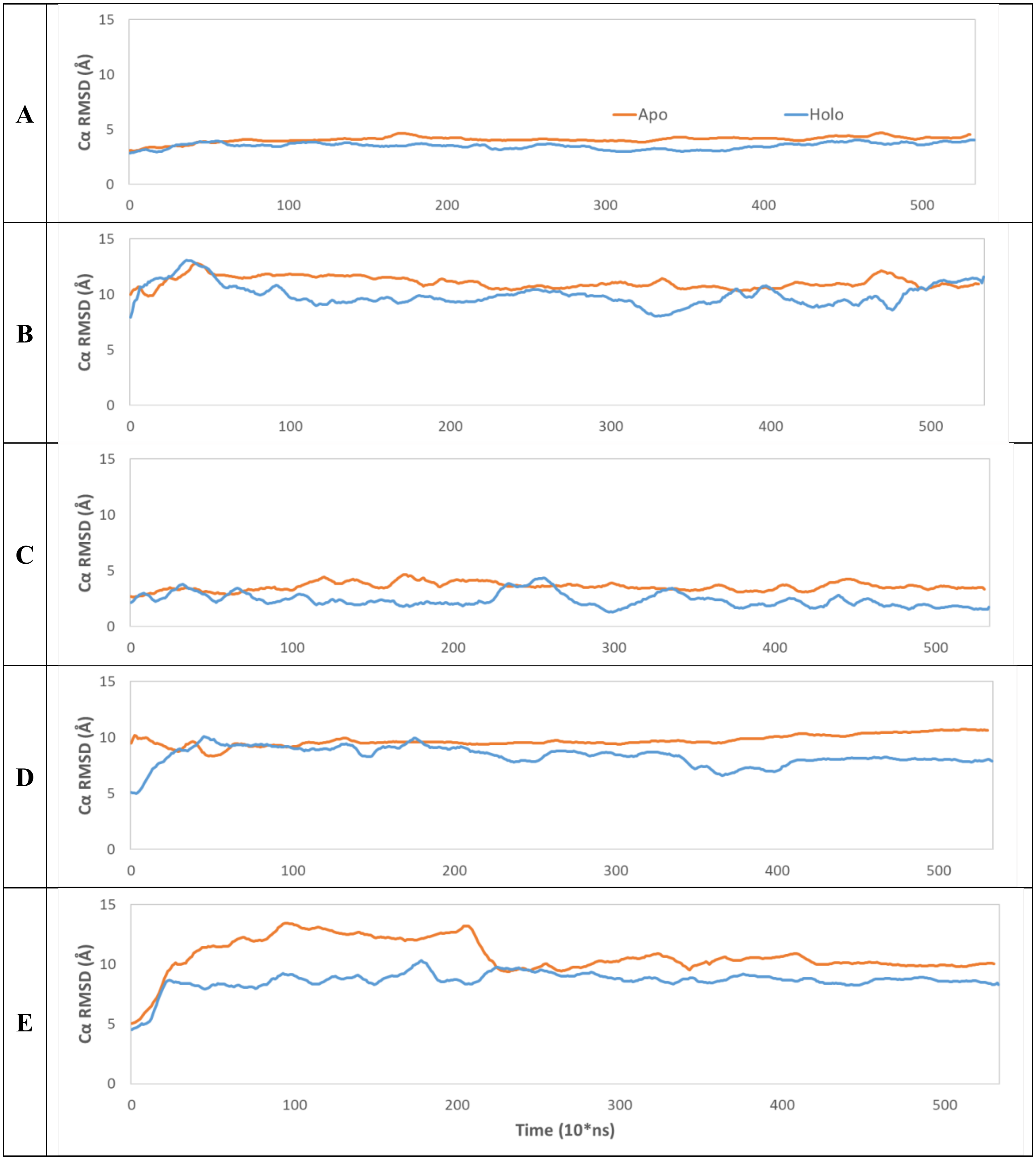

Figure S30. The development of the Cα-RMSD of the receptor domains in apo-form (orange) and holo-form (blue) by fitting to the domain self for the third trajectory. **A:** AB. **B**: HI. **C**: TM. **D**: CS. **E**: SI.

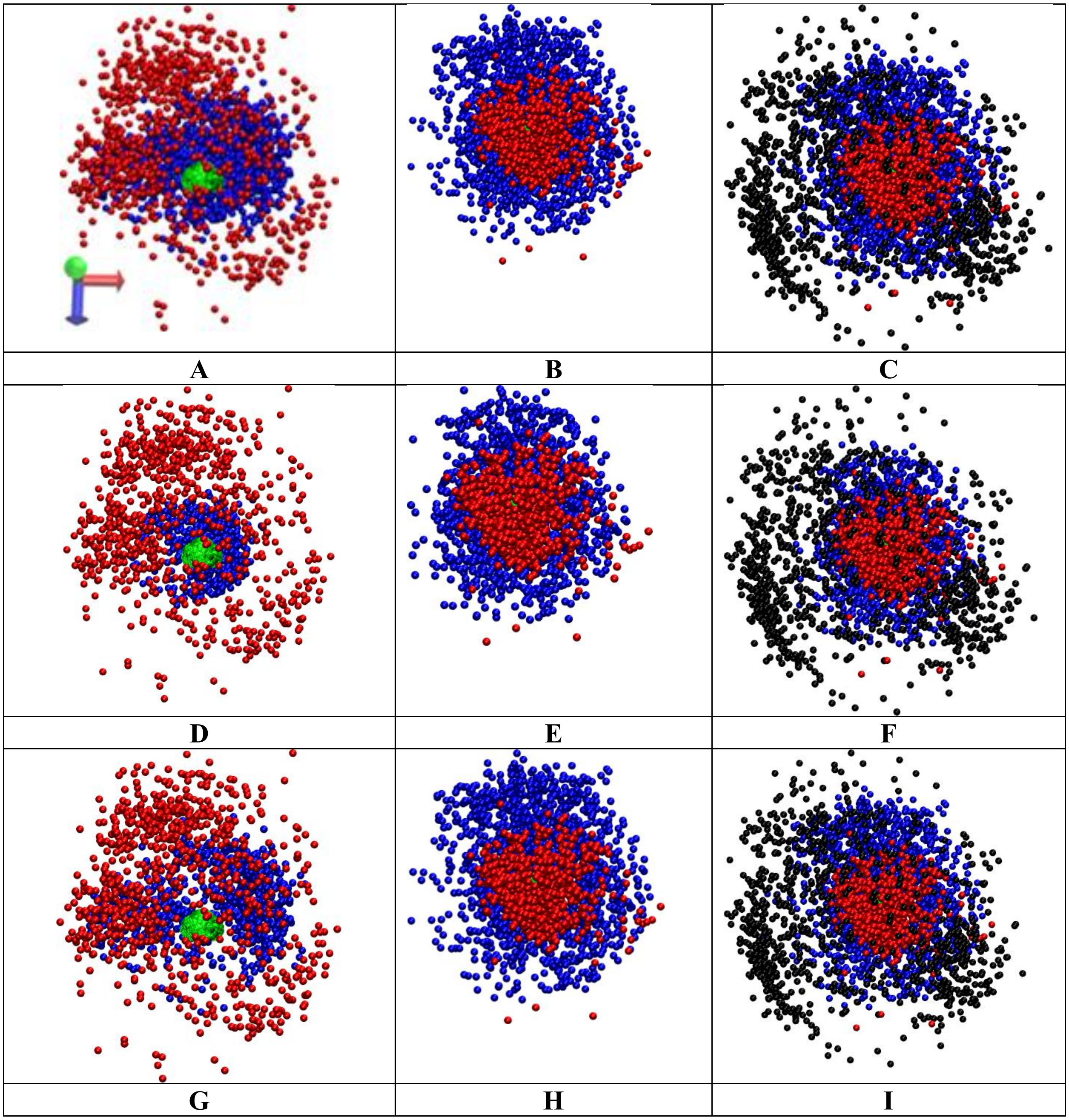

Figure S31. The center distribution change of the key domains of the receptor upon binding to AG for all three combined trajectories. Key domains presented from a top viewpoint (see **Figure 5, S31** for the other two viewpoints): AB (red); CS and SI (blue), and AG (black) with aligned TM (green). The side viewpoint is indicated by the three axes (A), in which Z (green) is the transmembrane direction; and X (red) and Y(blue) are the membrane plane. Domain distribution of CS and SI combined in apo-form (**A**) and holo-form without AG (**B**) for clarity and with AG (**C**), CS only in apo-form (**D**) and holo-form without AG (**E**) for clarity and with AG (**F**), and SI only in apo-form (**G**) and holo-form without AG (**H**) for clarity and with AG (**I**).

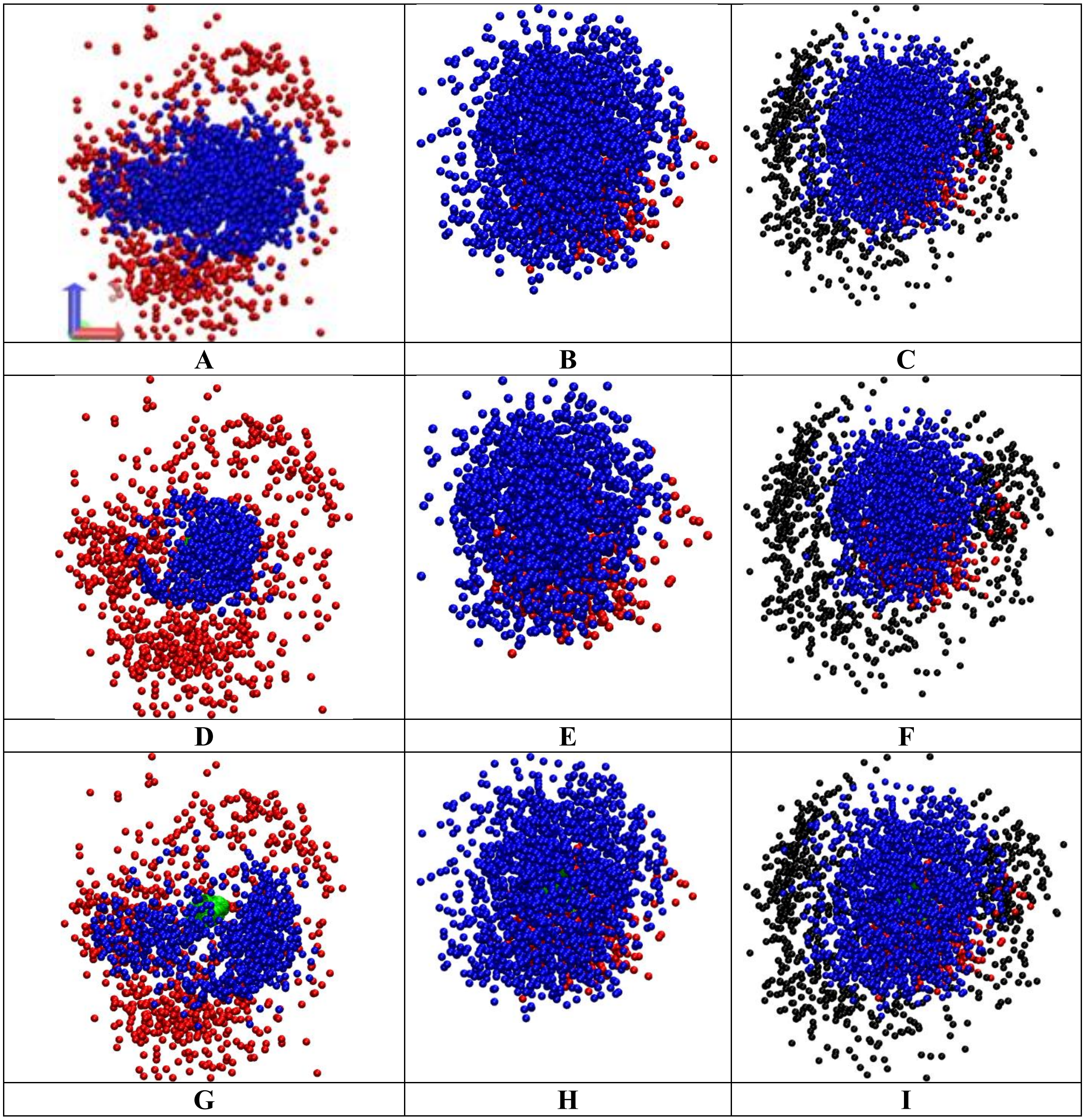

Figure S32. The center distribution change of the key domains of the receptor upon binding to AG for all three combined trajectories. Key domains presented from a bottom viewpoint (see **Figure 4, S32** for the other two viewpoints): AB (red); CS and SI (blue), and AG (black) with aligned TM (green). The side viewpoint is indicated by the three axes (A), in which Z (green) is the transmembrane direction; and X (red) and Y(blue) are the membrane plane. Domain distribution of CS and SI combined in apo-form (**A**) and holo-form without AG (**B**) for clarity and with AG (**C**), CS only in apo-form (**D**) and holo-form without AG (**E**) for clarity and with AG (**F**), and SI only in apo-form (**G**) and holo-form without AG (**H**) for clarity and with AG (**I**).

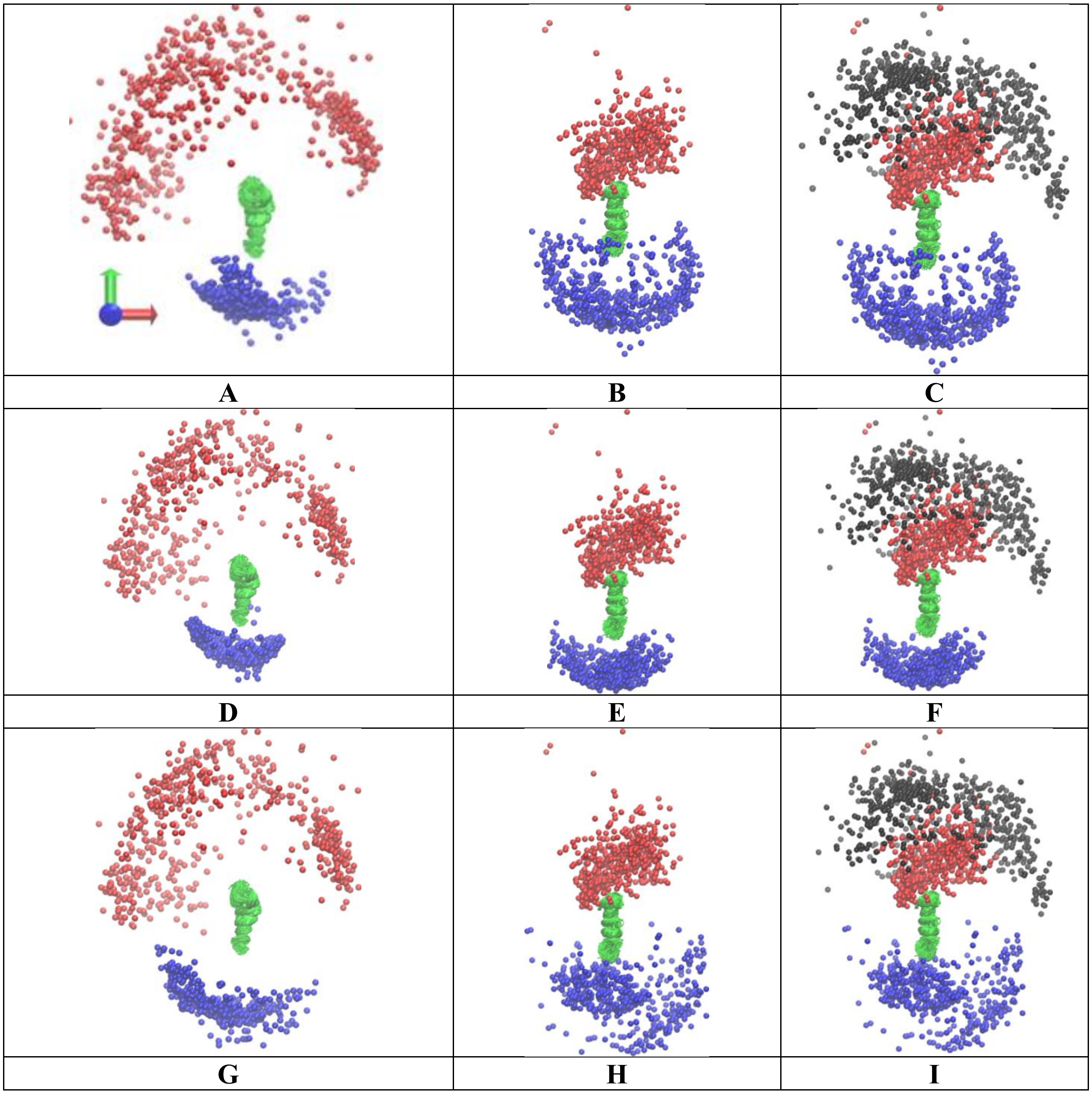

Figure S33. The center distribution change of the key domains of the receptor upon binding to AG for the first trajectory. Key domains presented from a side viewpoint (see **Fig. S34-35** for the other two viewpoints): extracellular AB (red); intracellular CS and SI (blue), and AG (black) with aligned TM (green) in membrane. The side viewpoint is indicated by the three axes (**A**), in which Z (green) is the transmembrane direction; and X (red) and Y(blue) are the membrane plane. The center distribution of combined CS and SI domains in apo-form (**A**) and holo-form without showing AG (**B**) for clarity and with AG (**C**), CS only in apo-form (**D**) and holo-form without showing AG (**E**) for clarity and with AG (**F**), and SI only in apo-form (**G**) and holo-form without showing AG (**H**) for clarity and with AG (**I**).

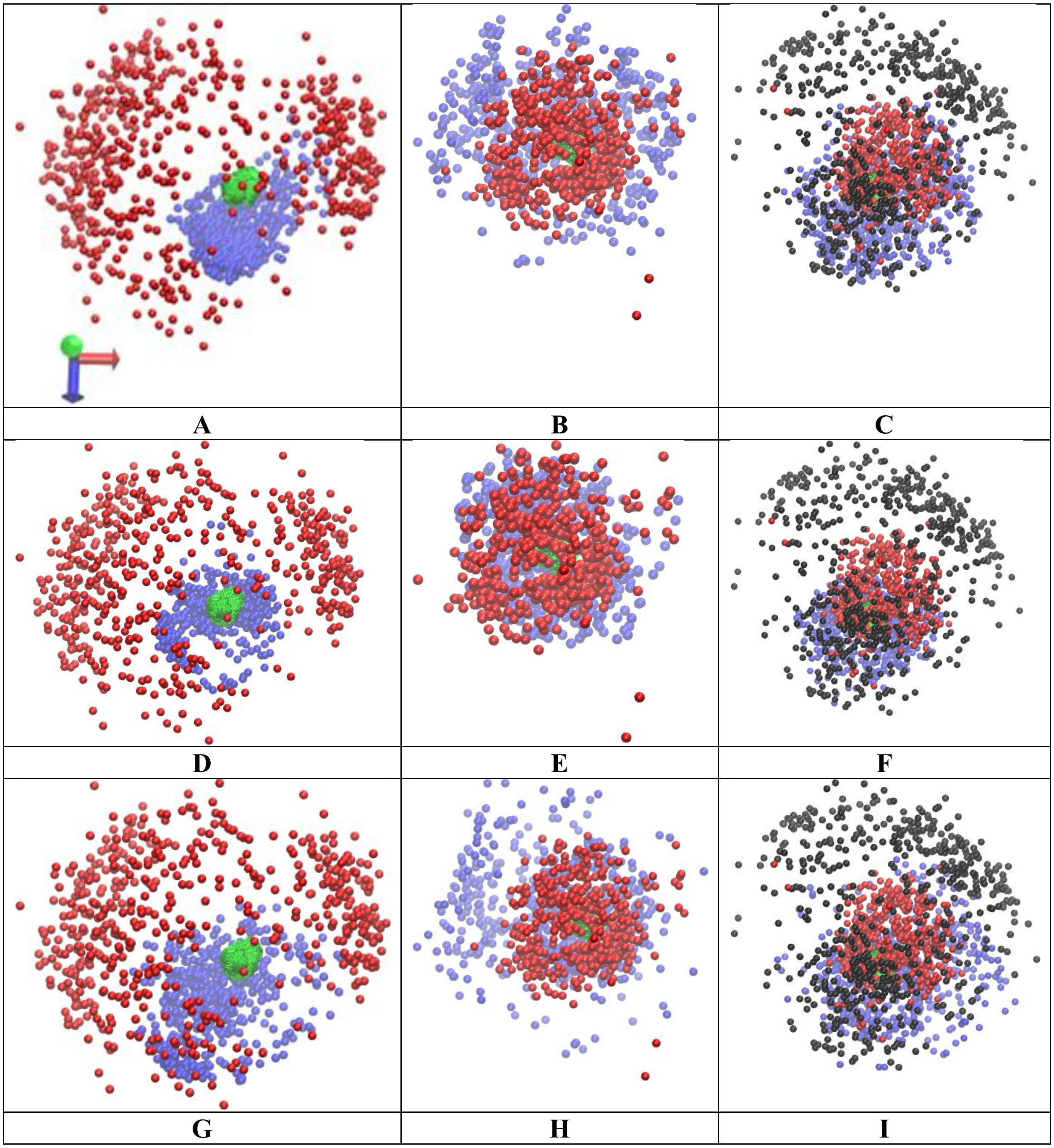

Figure S34. The center distribution change of the key domains of the receptor upon binding to AG **for the first trajectory**. Key domains presented from a top viewpoint (see **Figure S32, S33** for the other two viewpoints): AB (red); CS and SI (blue), and AG (black) with aligned TM (green). The side viewpoint is indicated by the three axes (A), in which Z (green) is the transmembrane direction; and X (red) and Y(blue) are the membrane plane. Domain distribution of CS and SI combined in apo-form (**A**) and holo-form without AG (**B**) for clarity and with AG (**C**), CS only in apo-form (**D**) and holo-form without AG (**E**) for clarity and with AG (**F**), and SI only in apo-form (**G**) and holo-form without AG (**H**) for clarity and with AG (**I**).

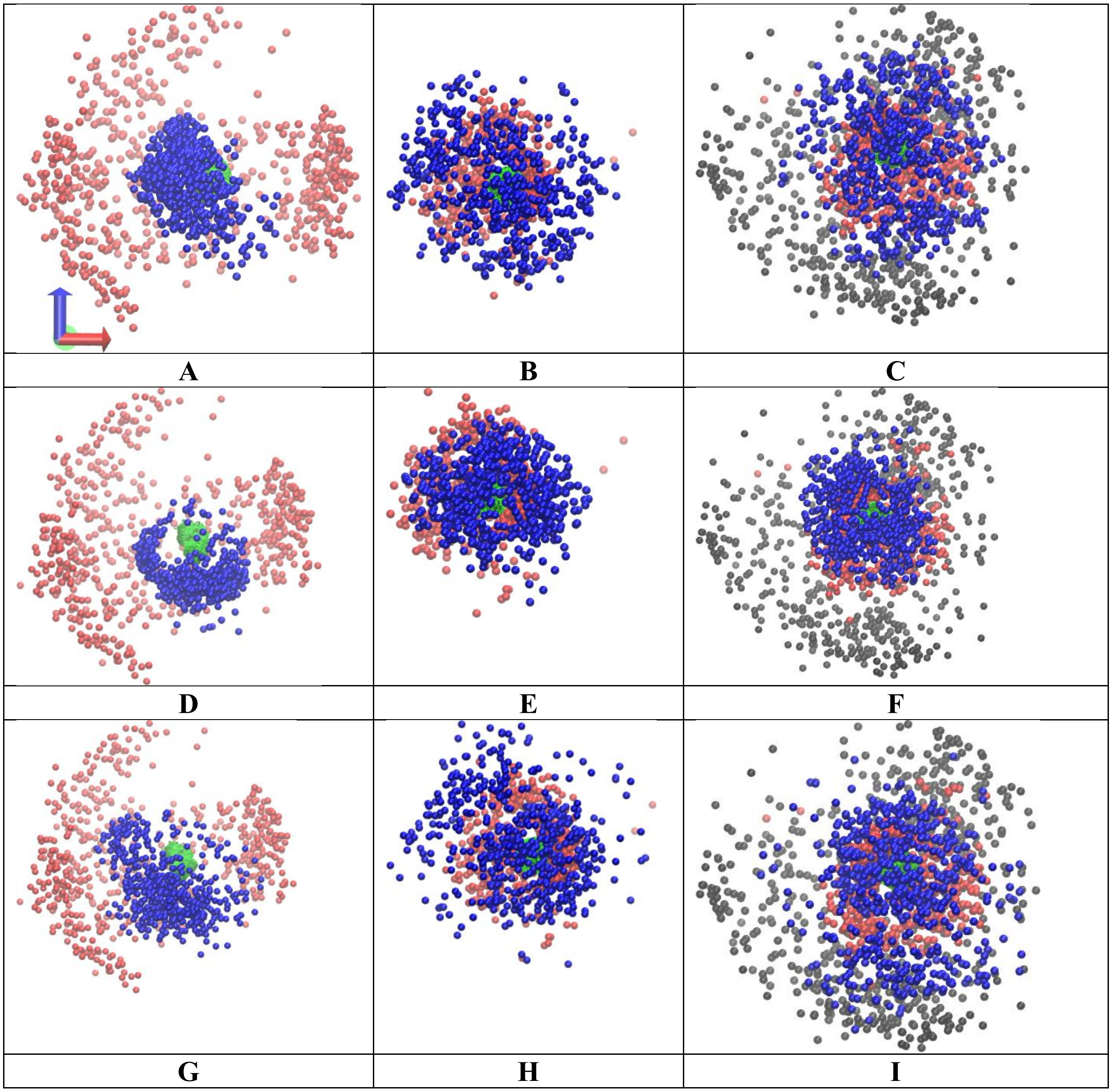

Figure S35. The center distribution change of the key domains of the receptor upon binding to AG for the first trajectory. Key domains presented from a bottom viewpoint (see **Figure S32-33** for the other two viewpoints): AB (red); CS and SI (blue), and AG (black) with aligned TM (green). The side viewpoint is indicated by the three axes (A), in which Z (green) is the transmembrane direction; and X (red) and Y(blue) are the membrane plane. Domain distribution of CS and SI combined in apo-form (**A**) and holo-form without AG (**B**) for clarity and with AG (**C**), CS only in apo-form (**D**) and holo-form without AG (**E**) for clarity and with AG (**F**), and SI only in apo-form (**G**) and holo-form without AG (**H**) for clarity and with AG (**I**).

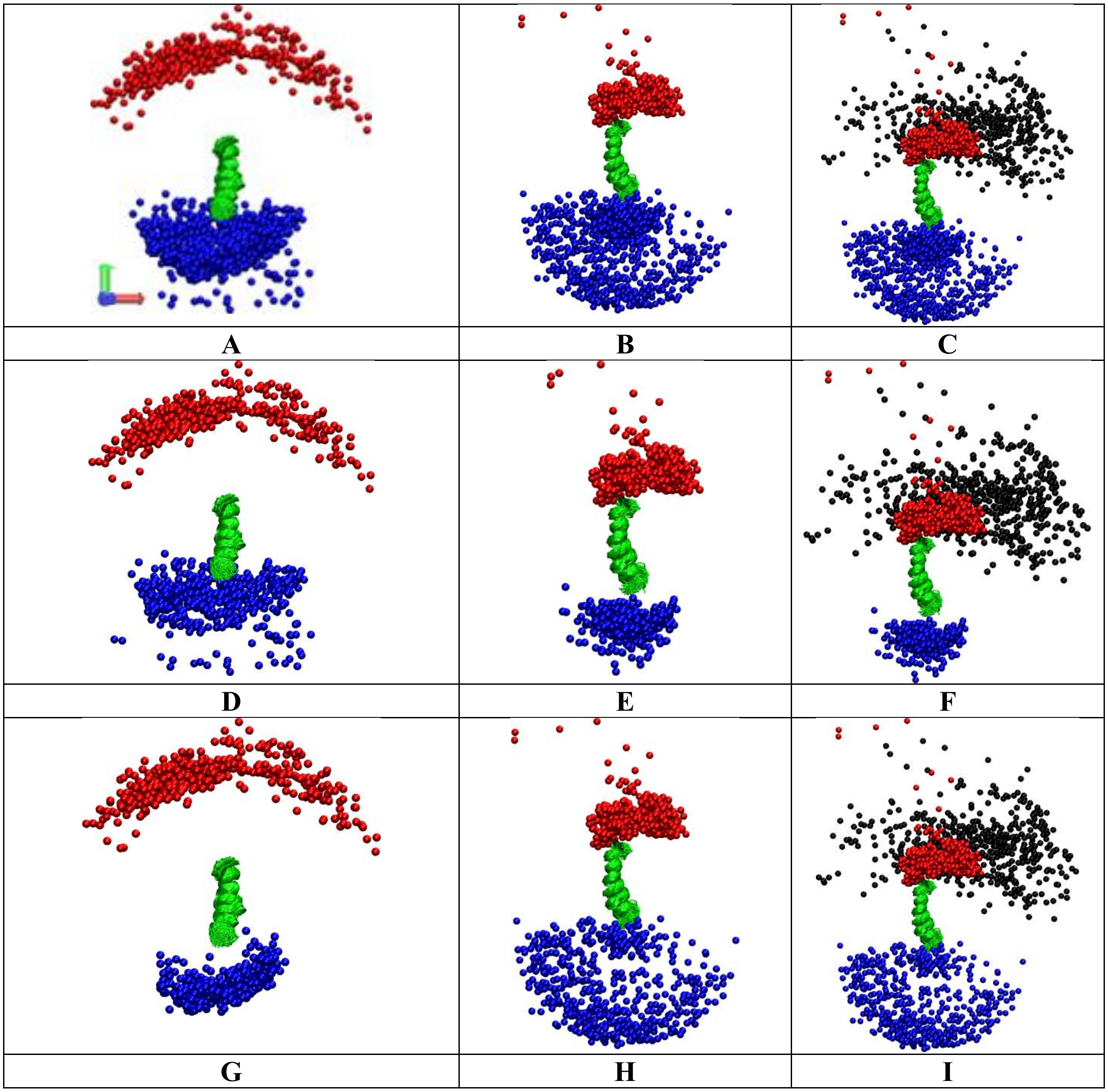

Figure S36. The center distribution change of the key domains of the receptor upon binding to AG for the second trajectory. Key domains presented from a side viewpoint (see **Fig. S36-37** for the other two viewpoints): extracellular AB (red); intracellular CS and SI (blue), and AG (black) with aligned TM (green) in membrane. The side viewpoint is indicated by the three axes (**A**), in which Z (green) is the transmembrane direction; and X (red) and Y(blue) are the membrane plane. Domain distribution of CS and SI combined in apo-form (**A**) and holo-form without AG (**B**) for clarity and with AG (**C**), CS only in apo-form (**D**) and holo-form without AG (**E**) for clarity and with AG (**F**), and SI only in apo-form (**G**) and holo-form without AG (**H**) for clarity and with AG (**I**).

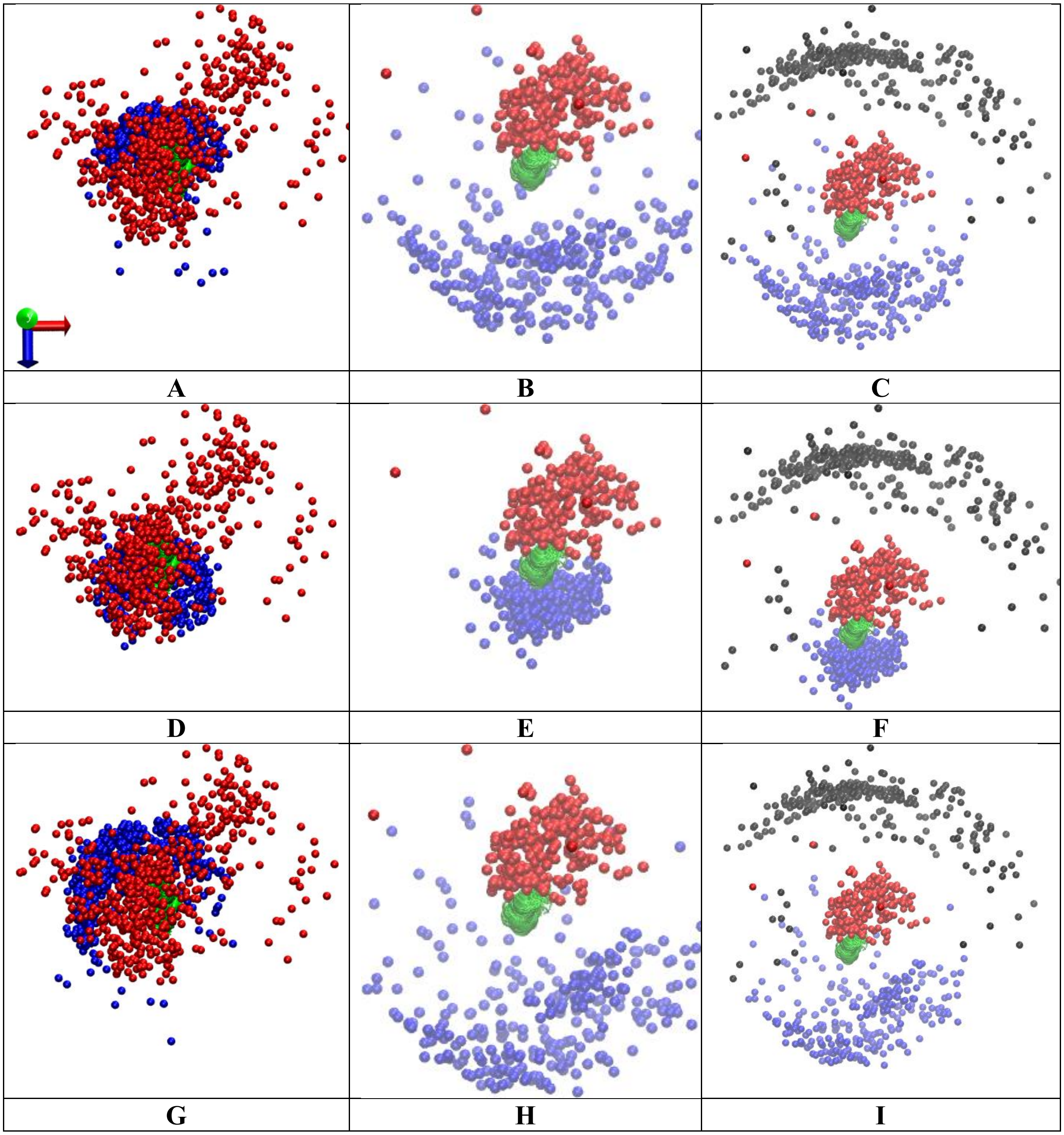

Figure S37. The center distribution change of the key domains of the receptor upon binding to AG for the second trajectory. Key domains presented from a top viewpoint (see **Figure S35, S37** for the other two viewpoints): AB (red); CS and SI (blue), and AG (black) with aligned TM (green). The side viewpoint is indicated by the three axes (A), in which Z (green) is the transmembrane direction; and X (red) and Y(blue) are the membrane plane. Domain distribution of CS and SI combined in apo-form (**A**) and holo-form without AG (**B**) for clarity and with AG (**C**), CS only in apo-form (**D**) and holo-form without AG (**E**) for clarity and with AG (**F**), and SI only in apo-form (**G**) and holo-form without AG (**H**) for clarity and with AG (**I**).

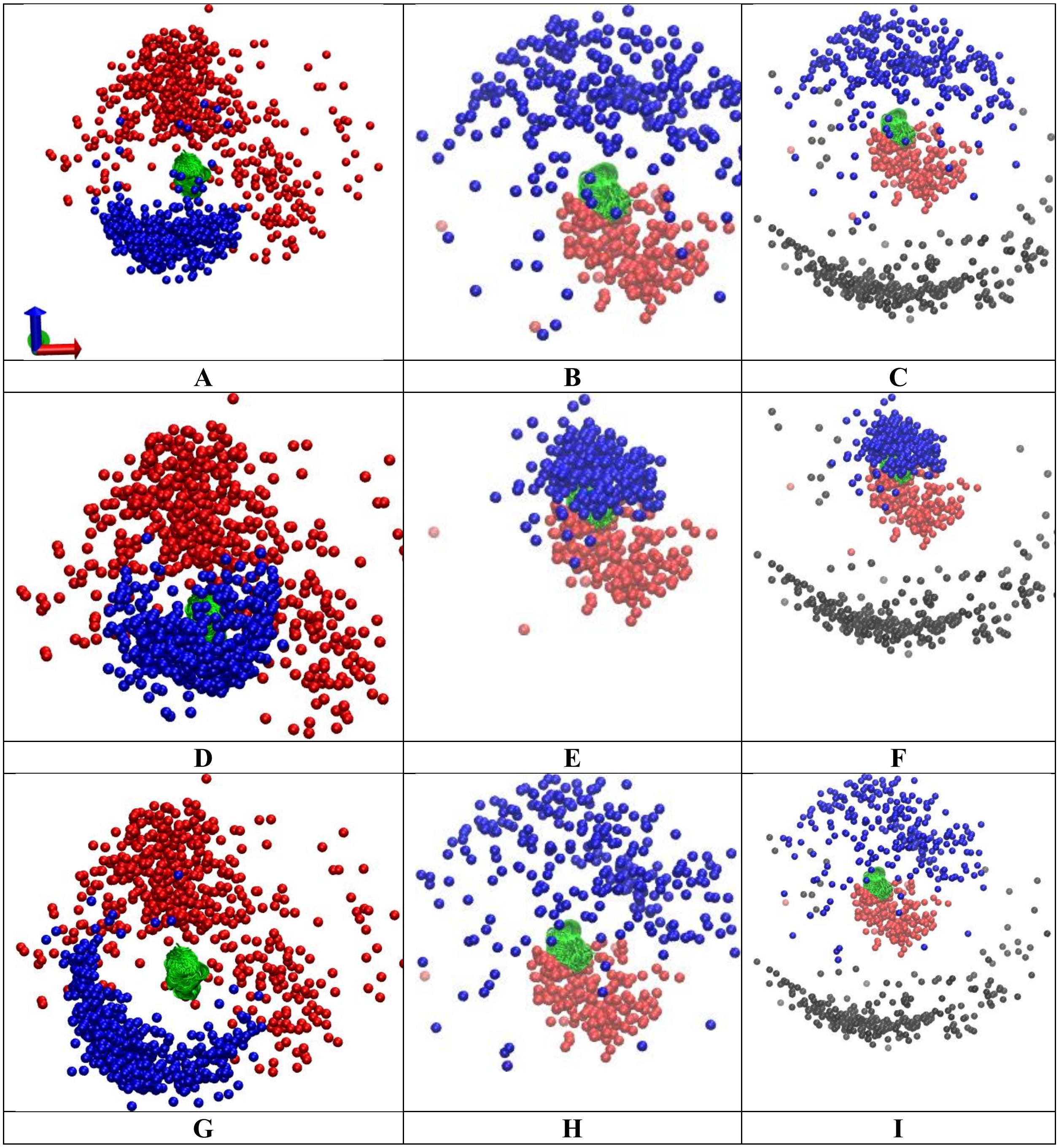

Figure S38. The center distribution change of the key domains of the receptor upon binding to AG for the second trajectory. Key domains presented from a bottom viewpoint (see **Figure S35-36** for the other two viewpoints): AB (red); CS and SI (blue), and AG (black) with aligned TM (green). The side viewpoint is indicated by the three axes (A), in which Z (green) is the transmembrane direction; and X (red) and Y(blue) are the membrane plane. Domain distribution of CS and SI combined in apo-form (**A**) and holo-form without AG (**B**) for clarity and with AG (**C**), CS only in apo-form (**D**) and holo-form without AG (**E**) for clarity and with AG (**F**), and SI only in apo-form (**G**) and holo-form without AG (**H**) for clarity and with AG (**I**).

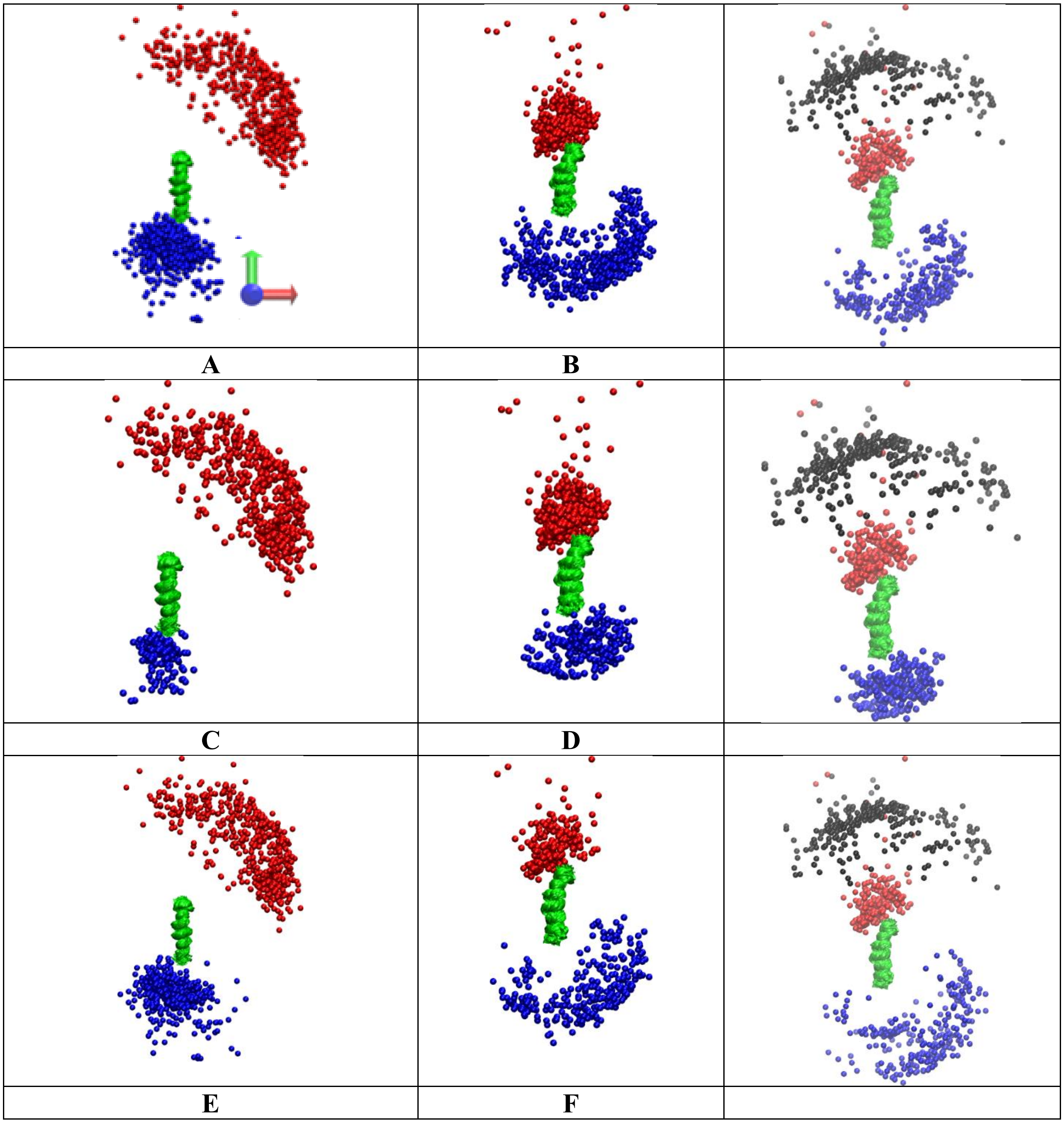

Figure S39. The center distribution change of the key domains of the receptor upon binding to AG for the third trajectory. Key domains presented from a side viewpoint (see **Fig. S39-40** for the other two viewpoints): extracellular AB (red); intracellular CS and SI (blue), and AG (black) with aligned TM (green) in membrane. The side viewpoint is indicated by the three axes (**A**), in which Z (green) is the transmembrane direction; and X (red) and Y(blue) are the membrane plane. Domain distribution of CS and SI combined in apo-form (**A**) and holo-form without AG (**B**) for clarity and with AG (**C**), CS only in apo-form (**D**) and holo-form without AG (**E**) for clarity and with AG (**F**), and SI only in apo-form (**G**) and holo-form without AG (**H**) for clarity and with AG (**I**).

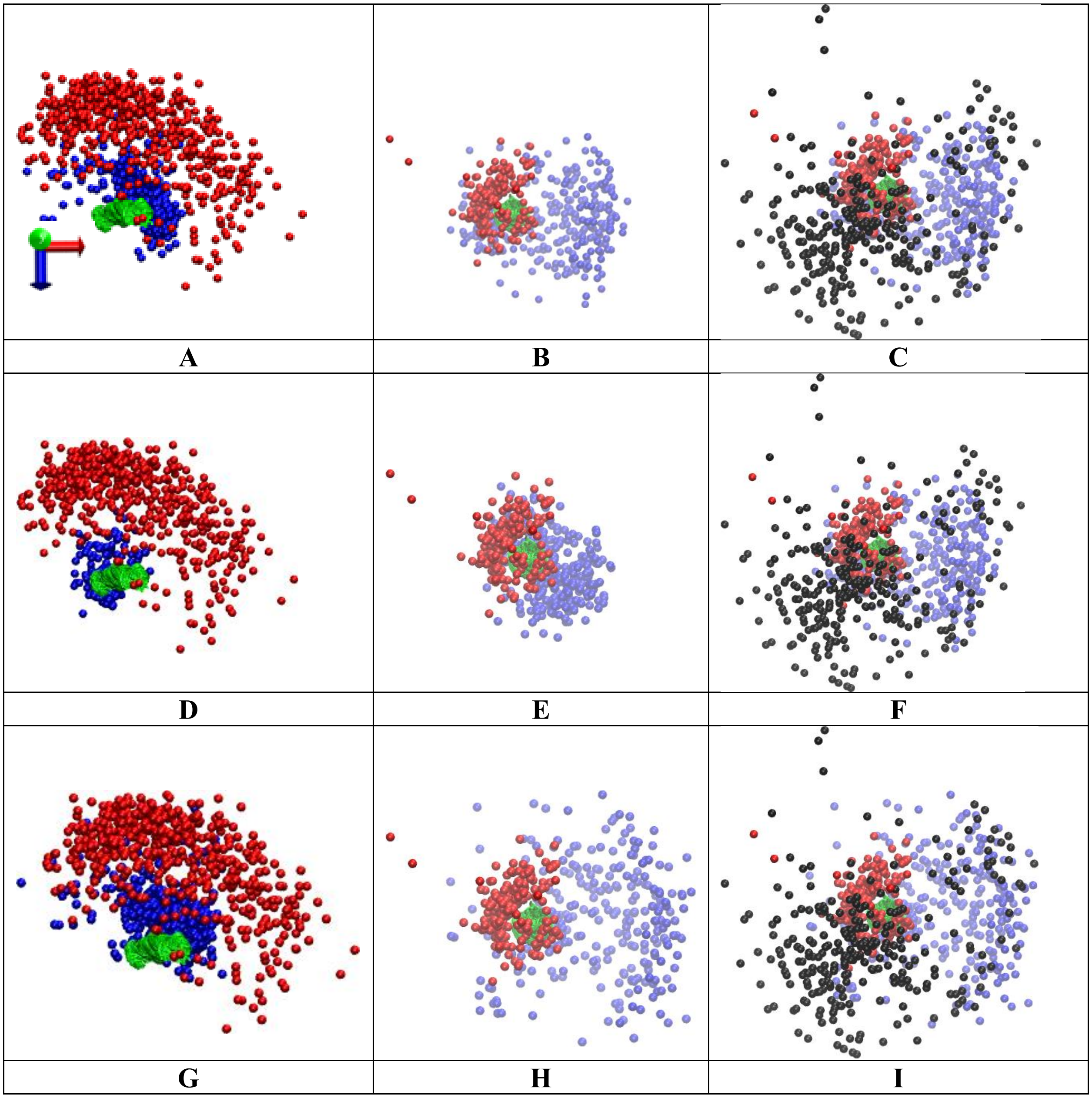

Figure S40. The center distribution change of the key domains of the receptor upon binding to AG for the third trajectory. Key domains presented from a top viewpoint (see **Figure S38, S40** for the other two viewpoints): AB (red); CS and SI (blue), and AG (black) with aligned TM (green). The side viewpoint is indicated by the three axes (A), in which Z (green) is the transmembrane direction; and X (red) and Y(blue) are the membrane plane. Domain distribution of CS and SI combined in apo-form (**A**) and holo-form without AG (**B**) for clarity and with AG (**C**), CS only in apo-form (**D**) and holo-form without AG (**E**) for clarity and with AG (**F**), and SI only in apo-form (**G**) and holo-form without AG (**H**) for clarity and with AG (**I**).

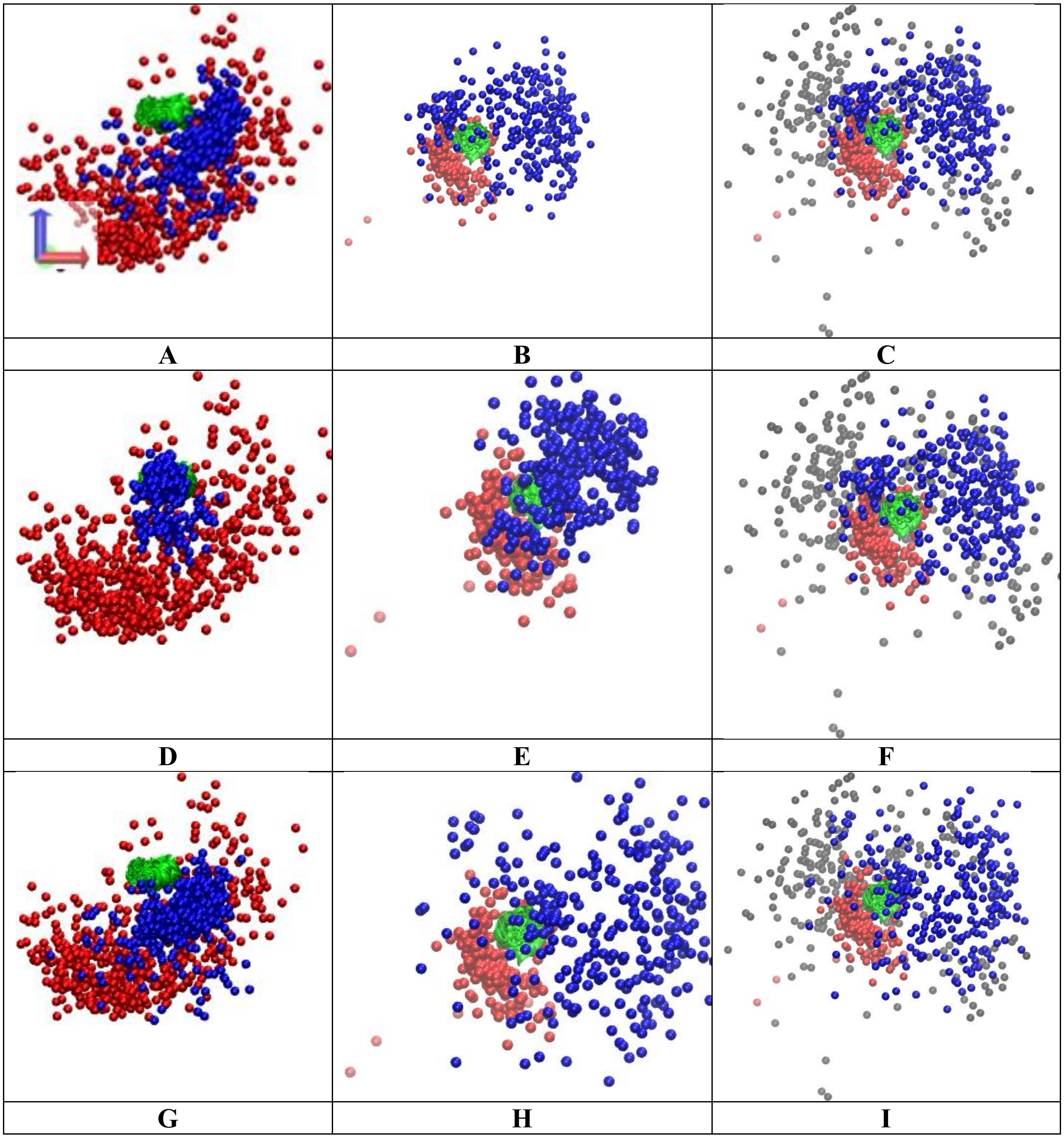

Figure S41. The center distribution change of the key domains of the receptor upon binding to AG for the third trajectory. Key domains presented from a bottom viewpoint (see **Figure S38-39** for the other two viewpoints): AB (red); CS and SI (blue), and AG (black) with aligned TM (green). The side viewpoint is indicated by the three axes (A), in which Z (green) is the transmembrane direction; and X (red) and Y(blue) are the membrane plane. Domain distribution of CS and SI combined in apo-form (**A**) and holo-form without AG (**B**) for clarity and with AG (**C**), CS only in apo-form (**D**) and holo-form without AG (**E**) for clarity and with AG (**F**), and SI only in apo-form (**G**) and holo-form without AG (**H**) for clarity and with AG (**I**).

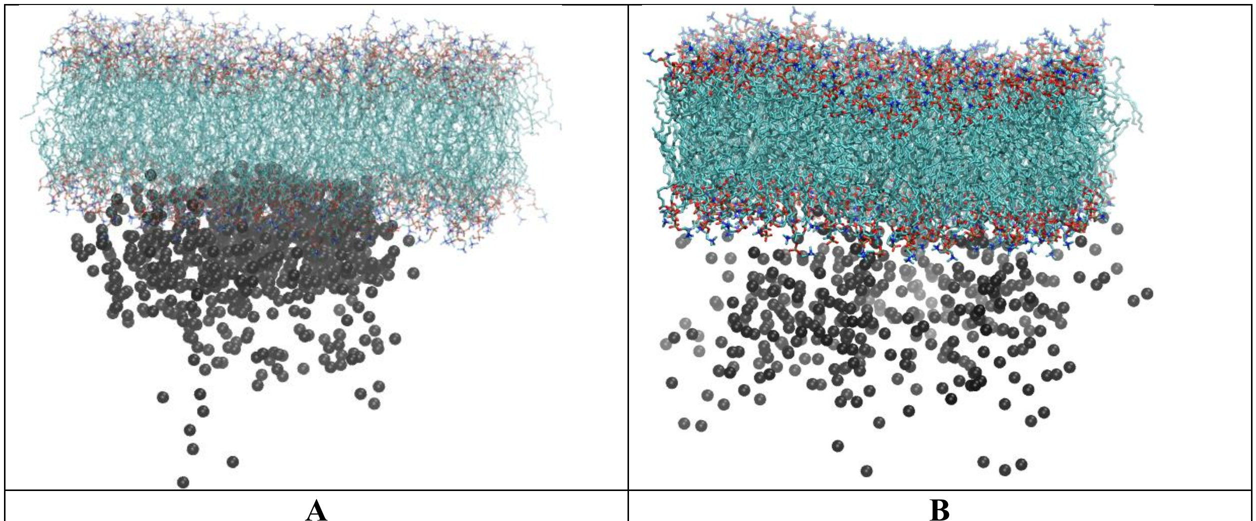

A	B

Figure S42. The center distribution change of the positively charged amino group of key SI polybasic residues upon binding to AG in apo-form (**A**) and holo-form (**B**) for the first trajectory. Nitrogen of the amino group of arginine and lysine of PBR 1 (residue 395 to 399) and PBR 2 (residue 406 to 410) in black.

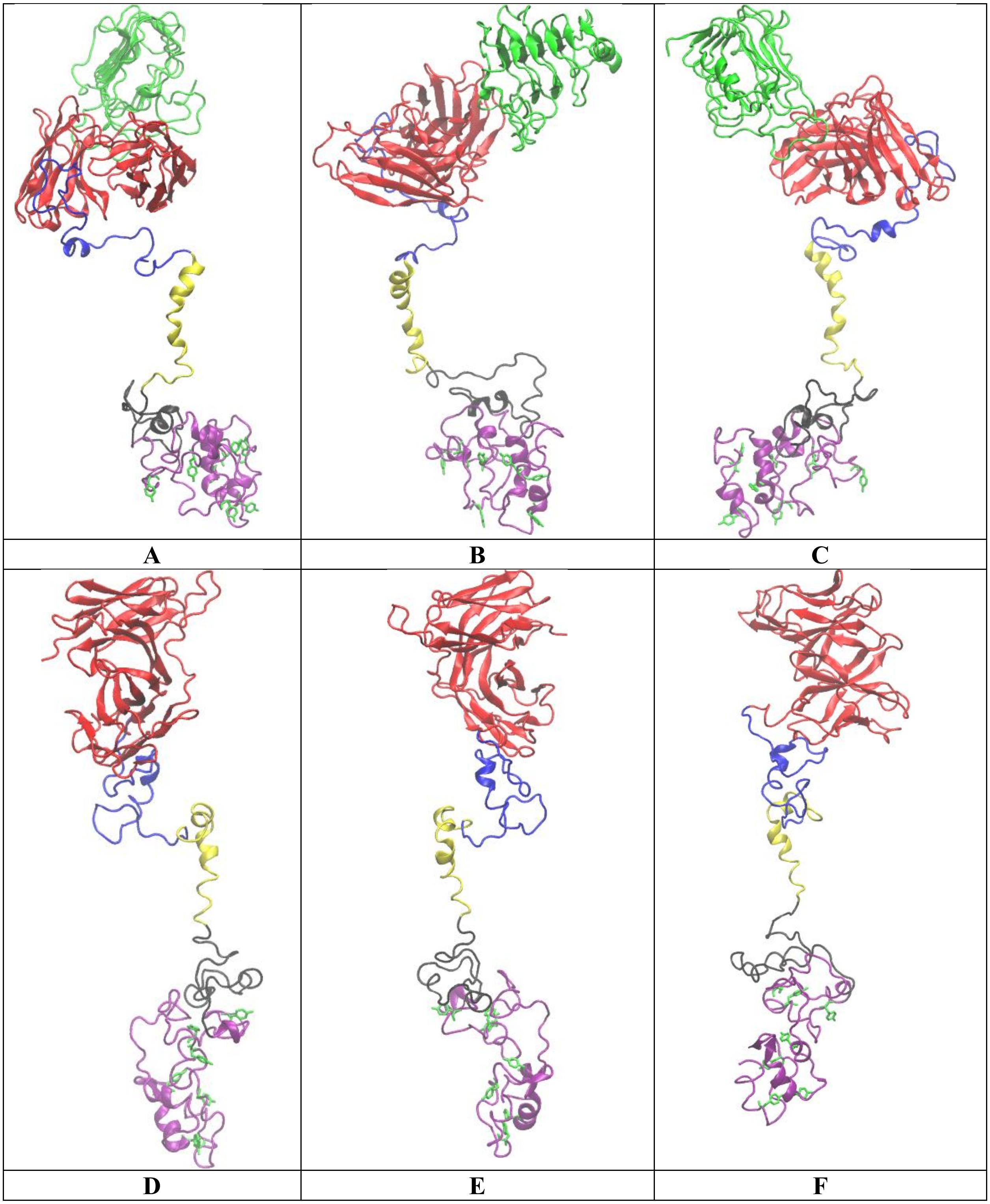

Figure S43. Last Snapshot of the simulation of holo-form (**A-C**) and apo-form (**D-F**) in three viewpoints in the first trajectory. AG in green, AB in red, HI in blue, TM in yellow, CS in pink, SI in purple with tyrosine residues in green.

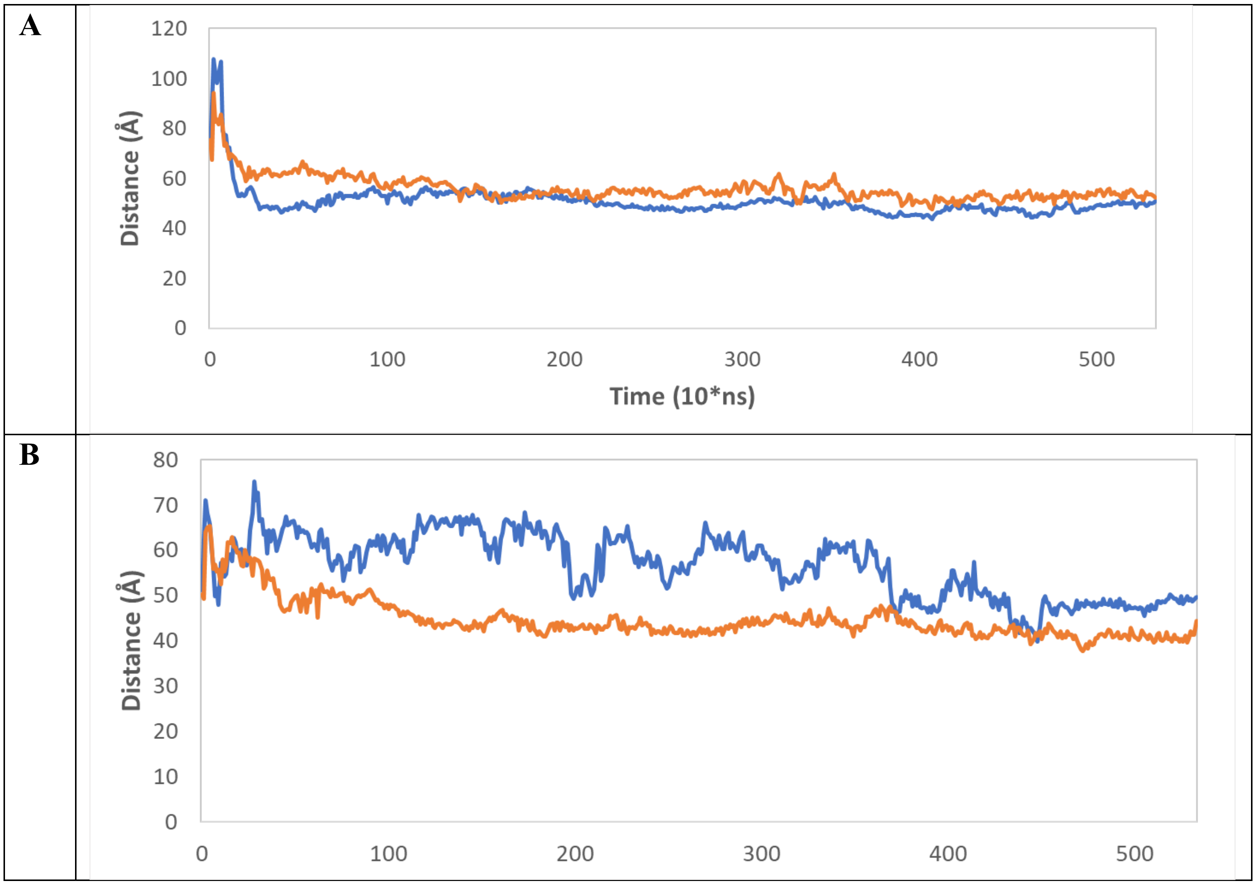

Figure S44. The development of the distance between various parts of the anti-HER2 CAR in the simulation of the apo-form in orange and the holo-form in blue **A**: the center of AB to the center of TM; **B**: the center of CS+SI to the center of TM.

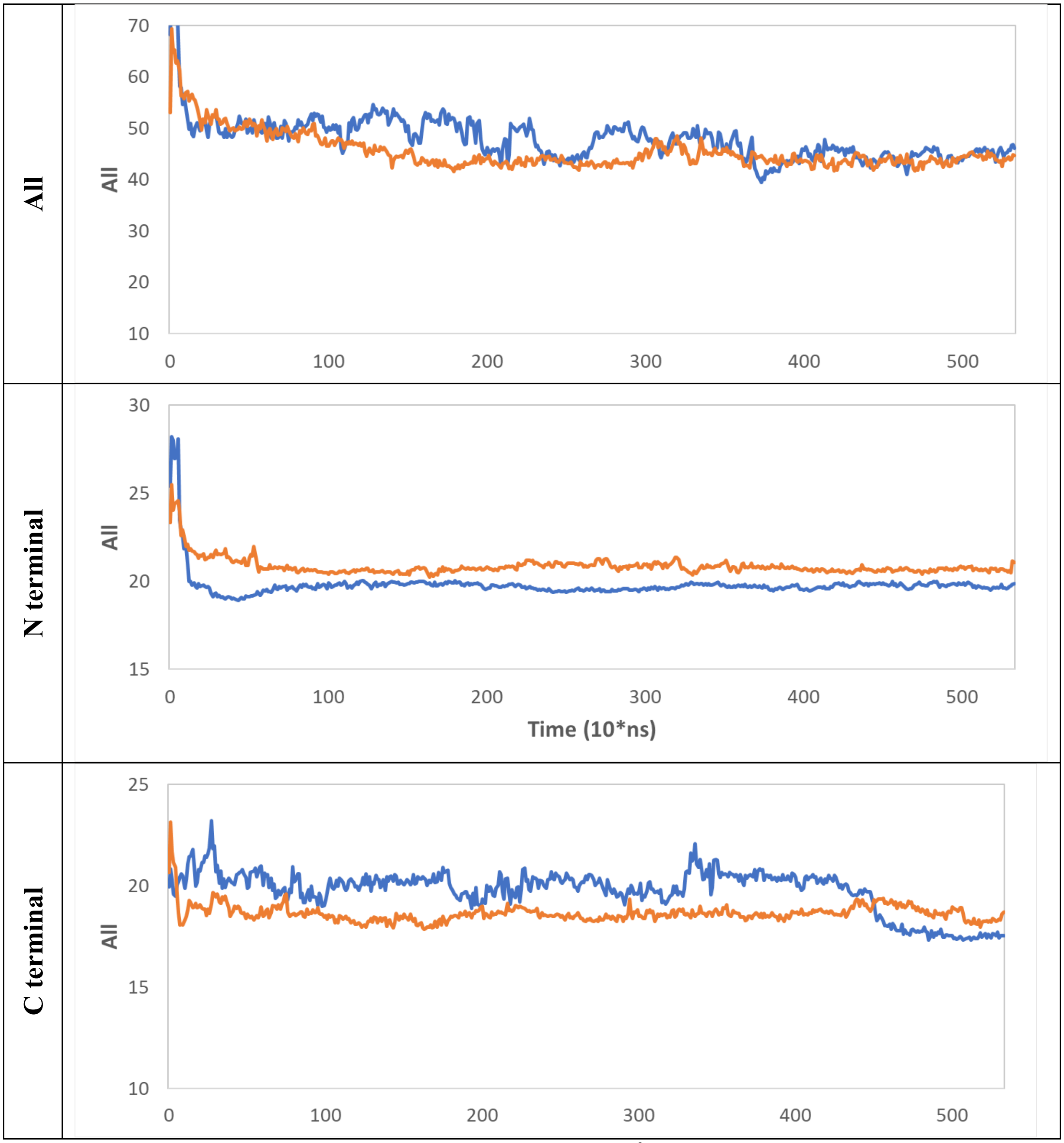

Figure S45. The development of the radius of gyration (Å) of various parts of the anti-HER2 CAR in the simulation of the apo-form in orange and the holo-form in blue **for all three combined trajectories.**

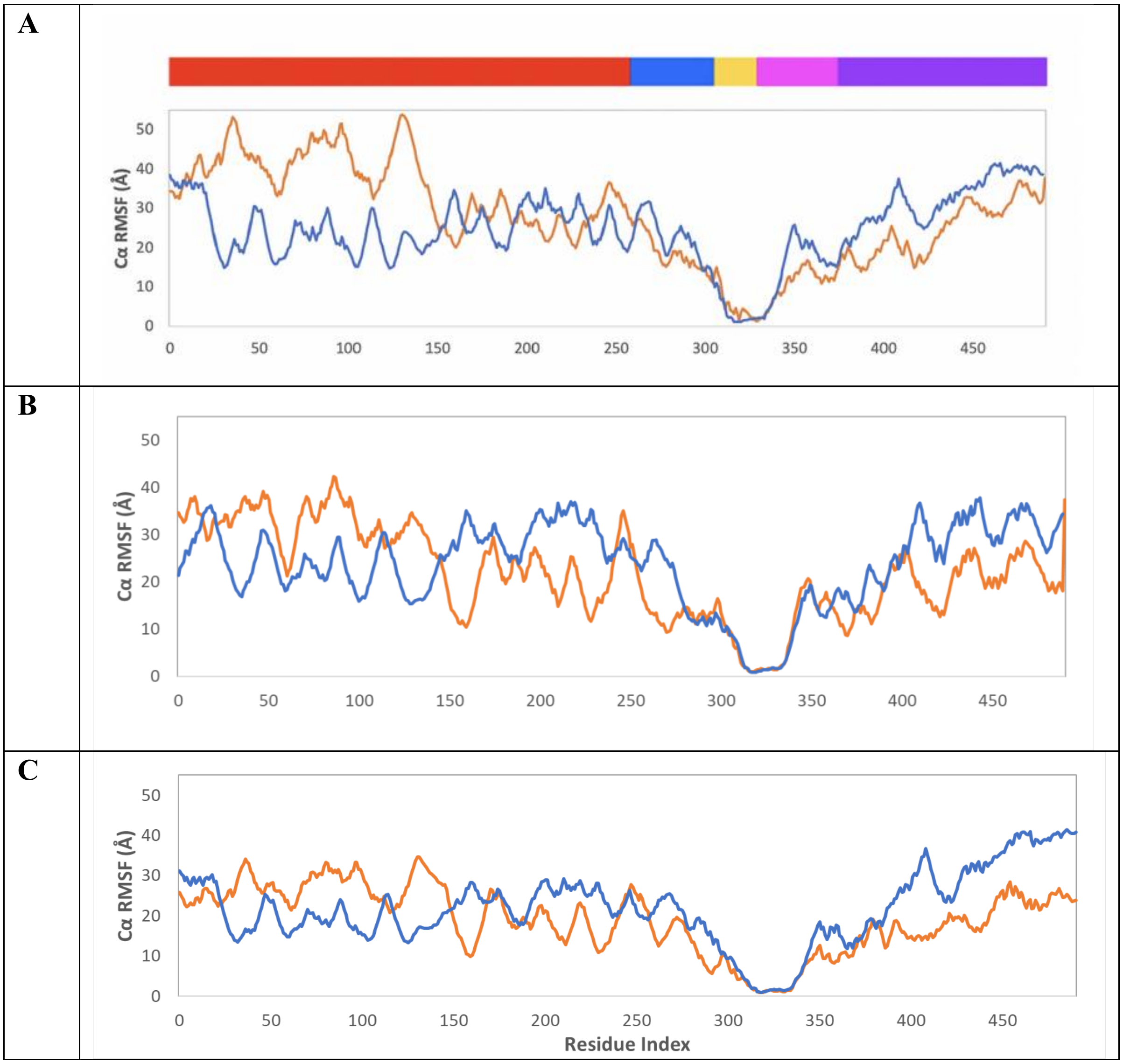

Figure S46. The average of Cα Root Mean Square Fluctuation (RMSF) profiles of the apo-form in orange and holo-form in blue **for the first (A), second (B), and third trajectories (D)**. Five structure domains are color coded starting from N-terminal region: AB in red, HI in blue, TM in yellow, CS in pink, SI in purple.

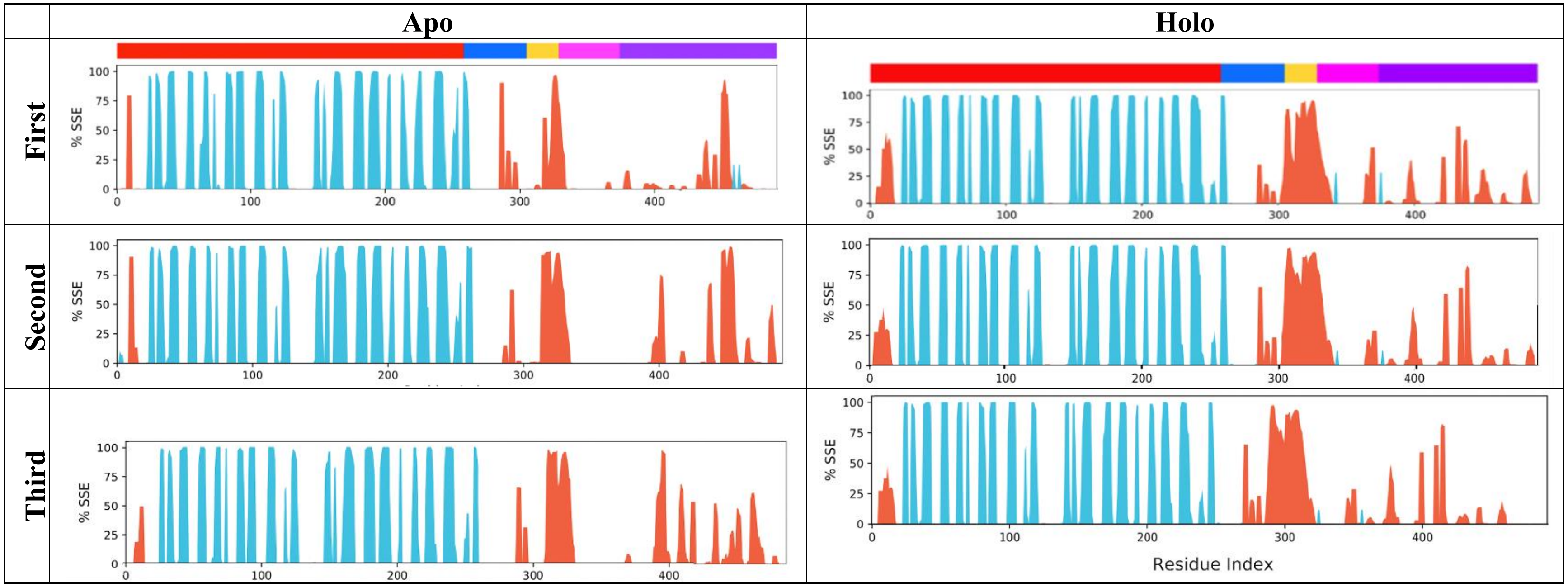

Figure S47. Secondary structure elements (SSE) of the apo-form and holo-form of the receptor for three individual trajectories**. A-B:** SSE distribution by residue index throughout the protein structure. **A:** Apo-form. **B:** Holo-form. The secondary structure is coded by color: α-helix in orange, β-sheet in blue, coil in white. Five structure domains are color coded starting from N-terminal region: AB in red, HI in blue, TM in yellow, CS in pink, SI in purple all 3 combined trajectories. Five structure domains are color coded starting from N-terminal region: AB in red, HI in blue, TM in yellow, CS in pink, SI in purple. All three combined trajectories are presented in **Figure 7.**

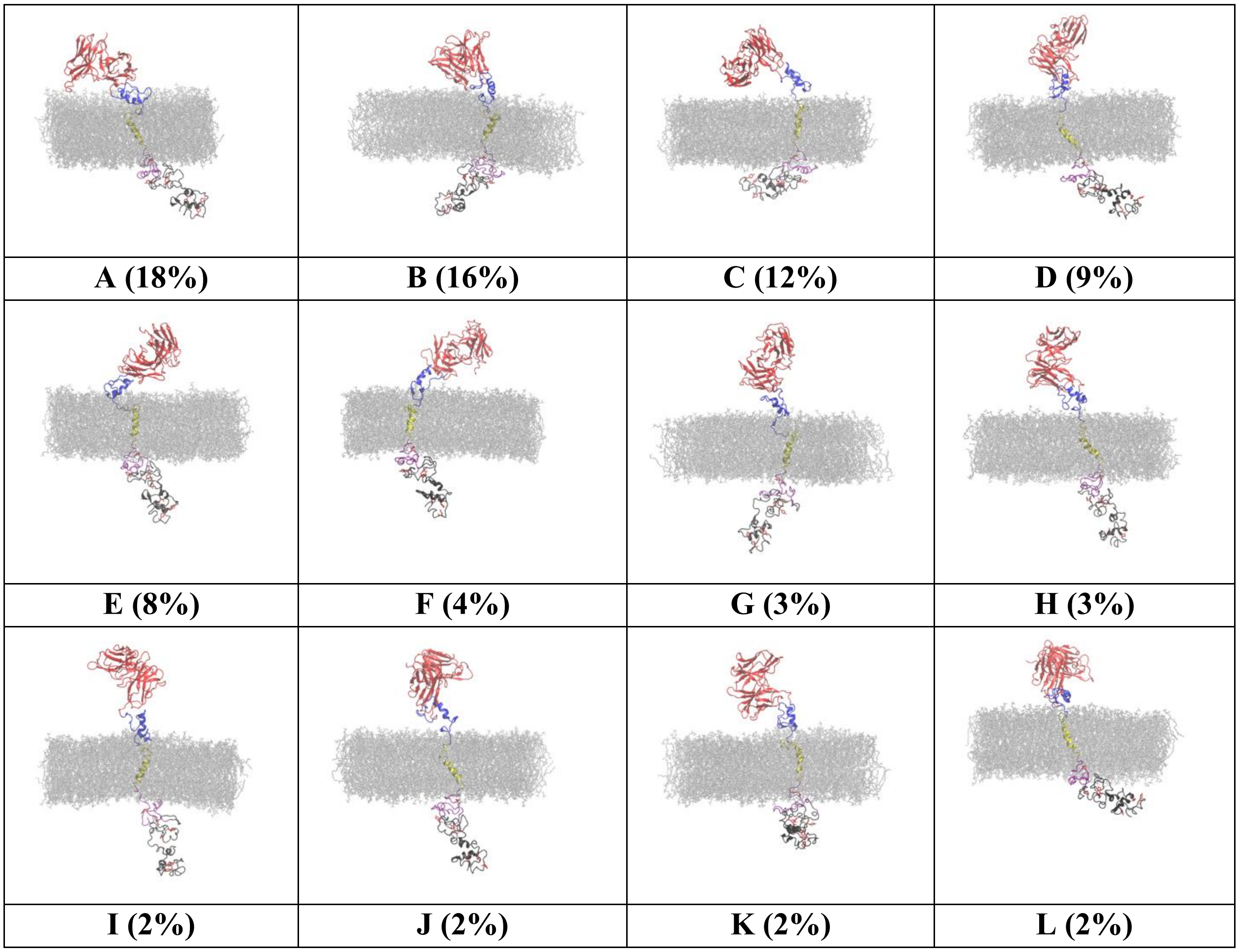

Figure S48. The representative structure of the most abundant clusters for apo-form in membrane accounting for ≥ 2% of total population from the first 5.4 μs trajectory. The abundance is annotated.

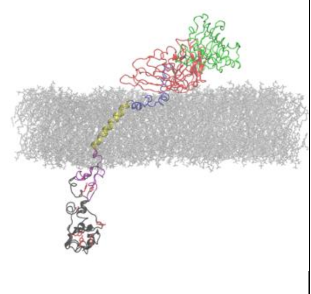	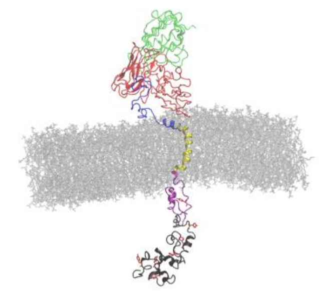	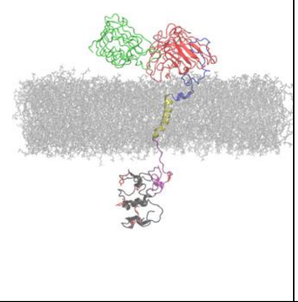	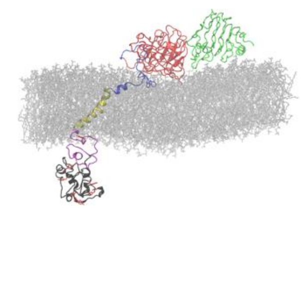
**A (8%)**	**B (7%)**	**C (6%)**	**D (6%)**
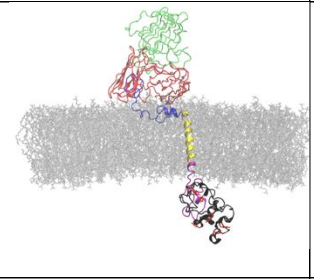	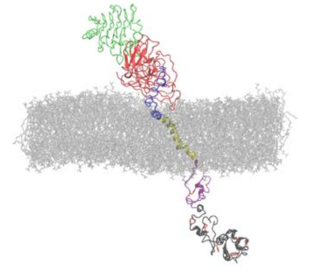	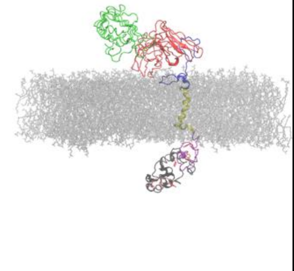	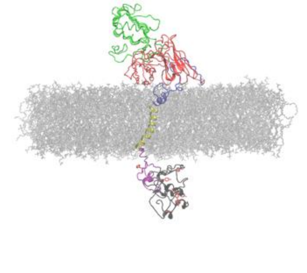
**E (6%)**	**F (6%)**	**G (5%)**	**H (4%)**
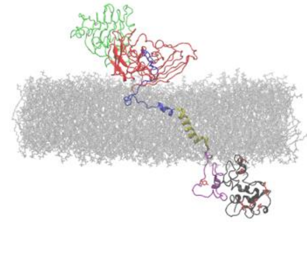	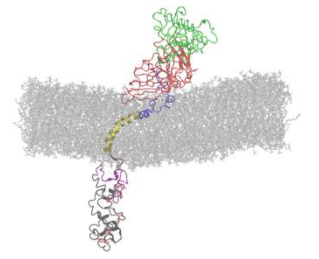	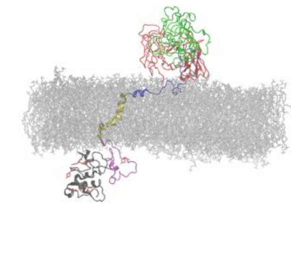	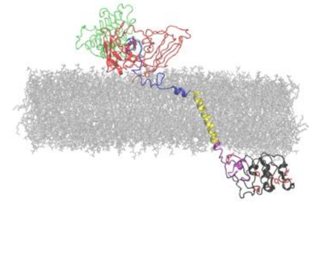
**I (4%)**	**J (4%)**	**K (4%)**	**L (4%)**
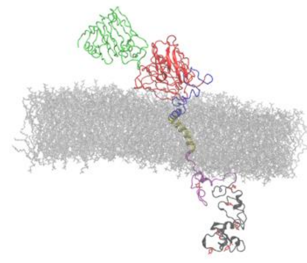	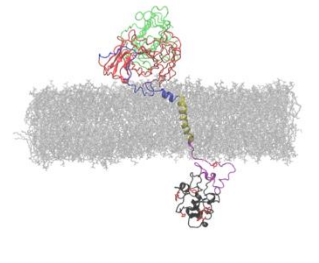	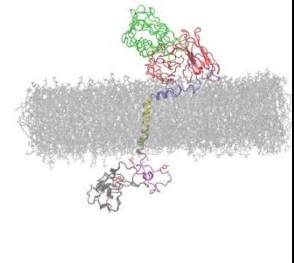	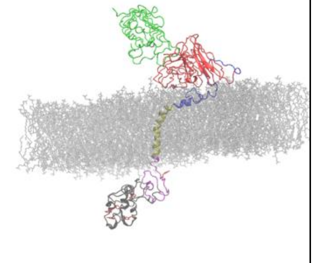
**M (3%)**	**N (3%)**	**O (3%)**	**P (3%)**
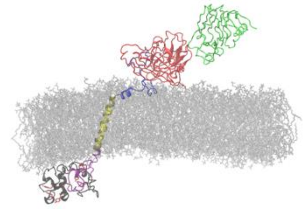	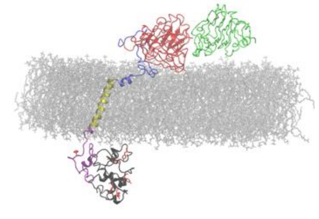	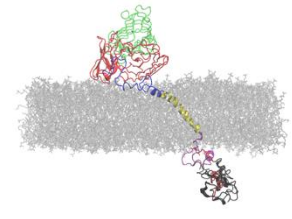	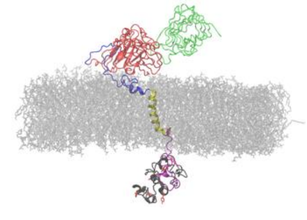
**Q (3%)**	**R (3%)**	**S (3%)**	**T (3%)**

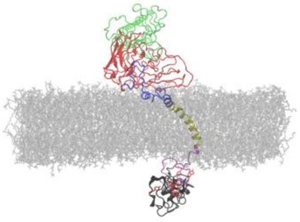	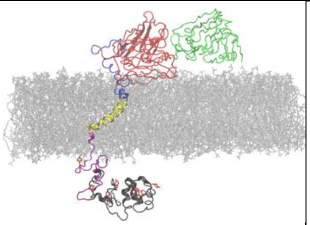		
**U (2%)**	**V (2%)**		

Figure S49. The representative structure of the most abundant clusters for holo-form in membrane of the first trajectory (10.5 us) accounting for ≥ 2% of total population. The abundance is annotated.

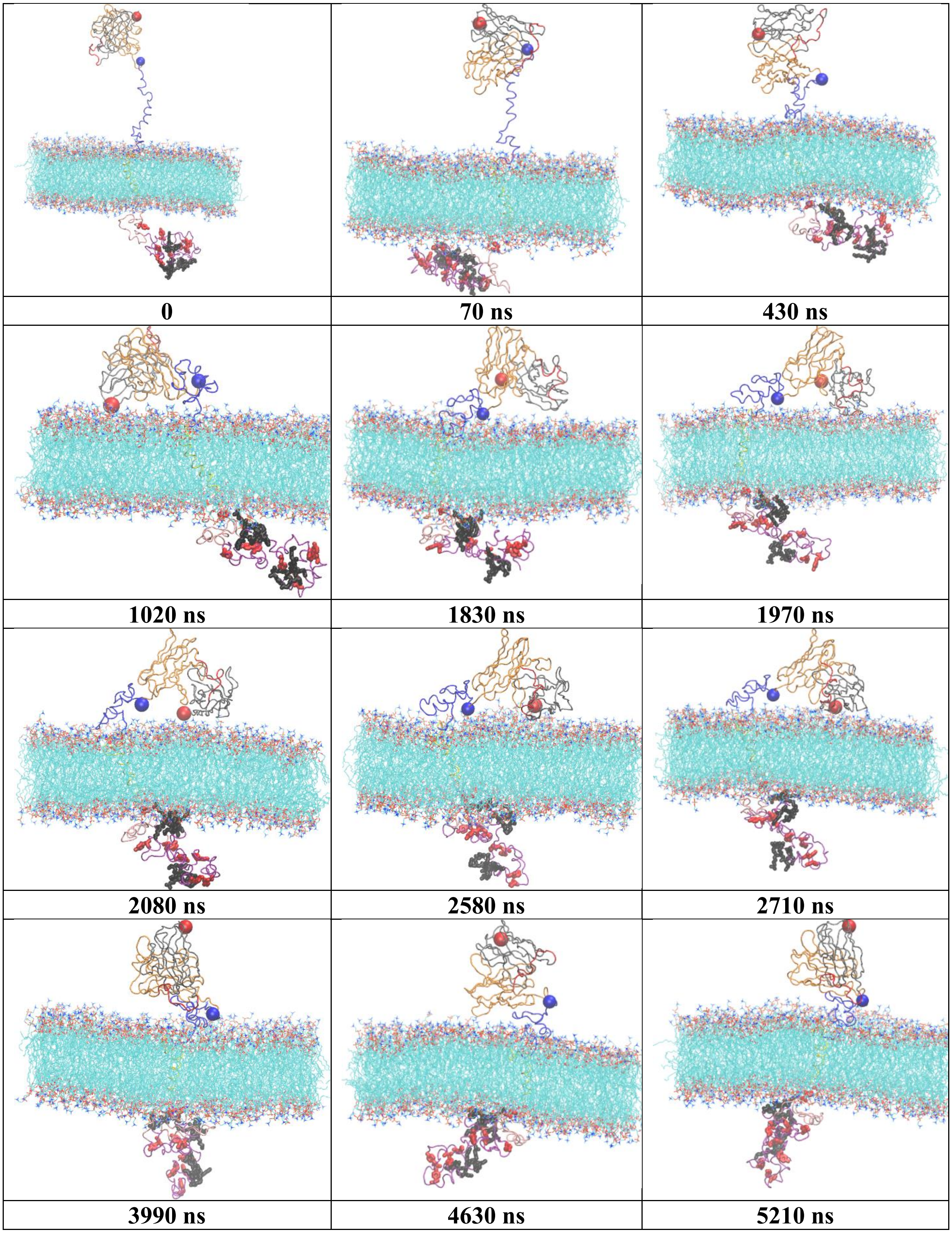

Figure S50. Interaction between polybasic regions of CD3ζ (black) and membrane for apo-form in the first trajectory of the apo-form system.

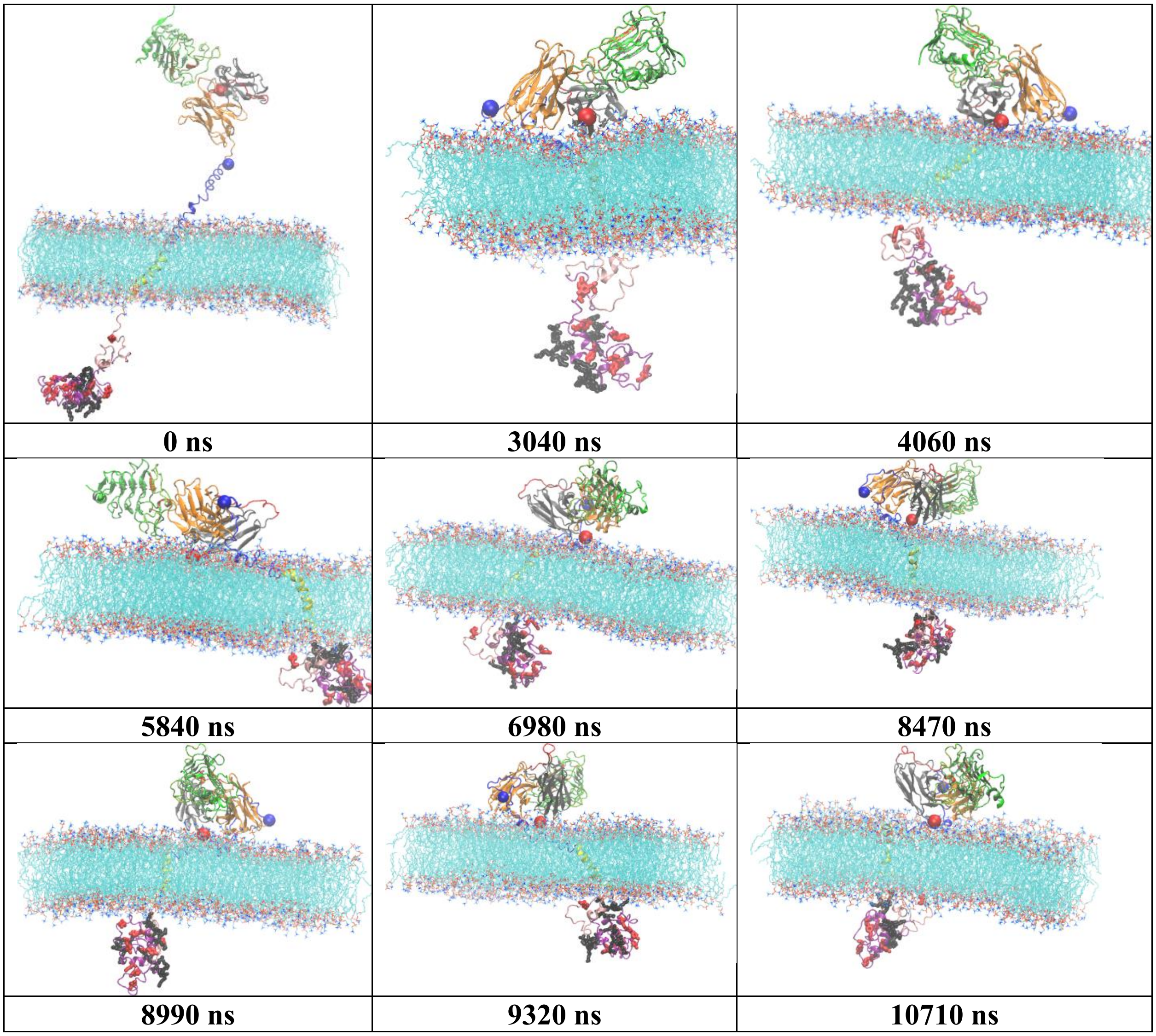

Figure S51. Interaction between polybasic regions of CD3ζ (black) and membrane for holo-form in the first trajectory.

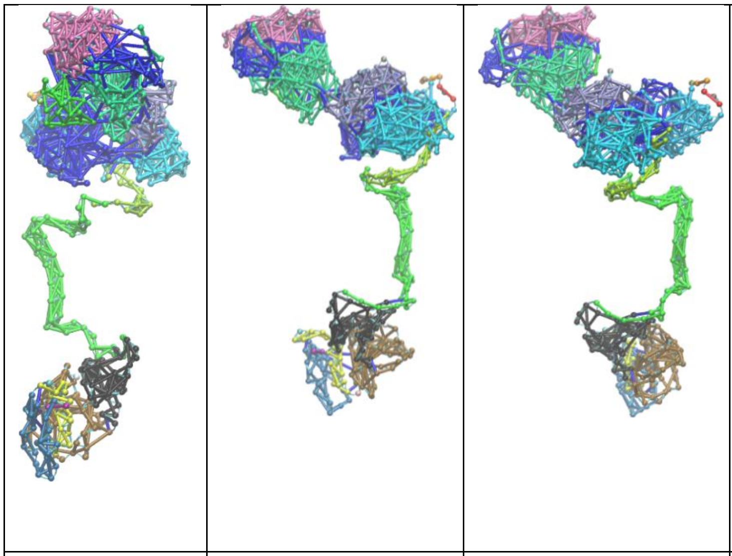

A B C D

E

Community #	Domain
1	AB
2	AB
3	AB
4	AB
5	AB
6	AB
7	AB
8	HI+TM+CS
9	SI
10	SI
11	SI
12	SI
13	AG
14	AG
15	SI
16	AB+AG
17	CS+SI
18	SI
19	AB+HI
20	AG
21	AG
22	AB
23	AB
24	SI
25	AG

Holo

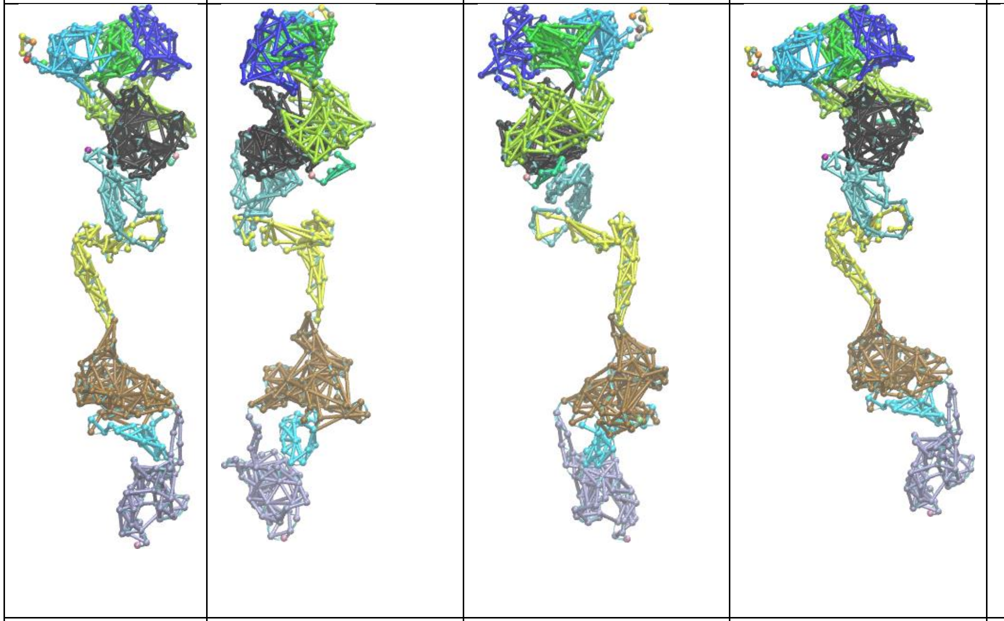

F G H I

J

Community #	Domain
1	AB
2	AB
3	AB
4	AB
5	AB
6	AB
	AB
8	AB
9	AB
10	AB
11	AB+HI
12	AB
13	SI
14	SI
15	HI+CS+SI
16	SI
17	AB
18	HI+TM
19	AB
20	AB
21	AB
22	SI
23	AB

Apo

Figure S52. Network communities for the receptor in holo-form (**A-E**) and apo-form (**F-J**) colored by community in different viewpoints.

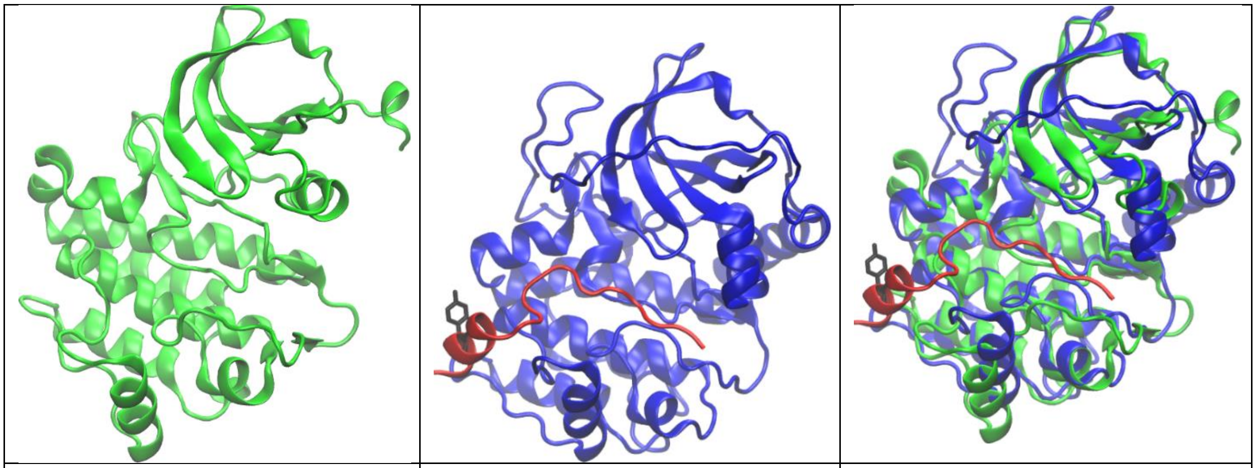

**A**	**B**	**C**

Figure S53. Structures of the Lck and kinase A in complex with pseudo-substrate peptide SP20. **A**: Lck (PDB: 3LCK); **B**: cAMP dependent protein kinase A in complex with pseudo-substrate peptide SP20 (PDB: 4IAC); **C**: 3LCK and 4IAC structures aligned. 3LCK in green, 4IAC kinase in blue, 4IAC peptide in red with a tyrosine residue in black.

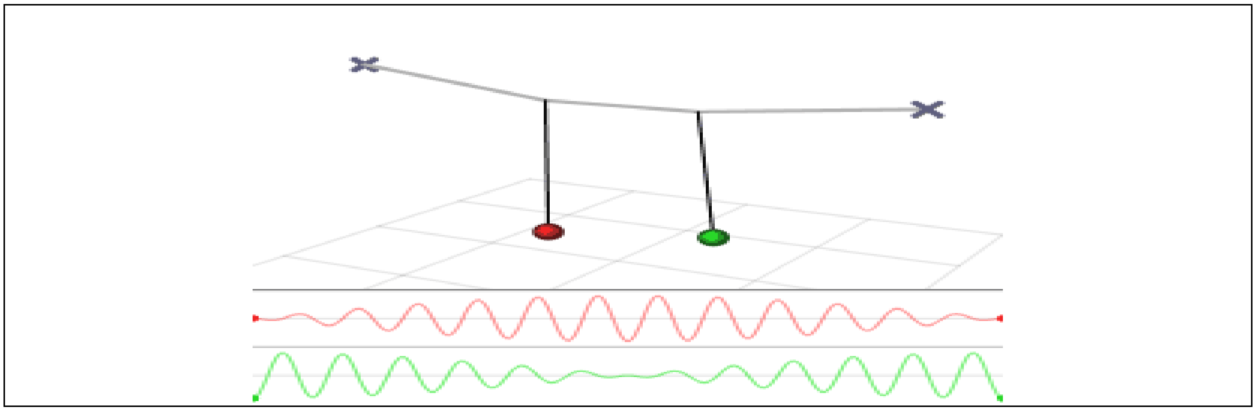

Figure S54. The model of a coupled pendulum representing Binding Induced Domain Flexibility Switch.

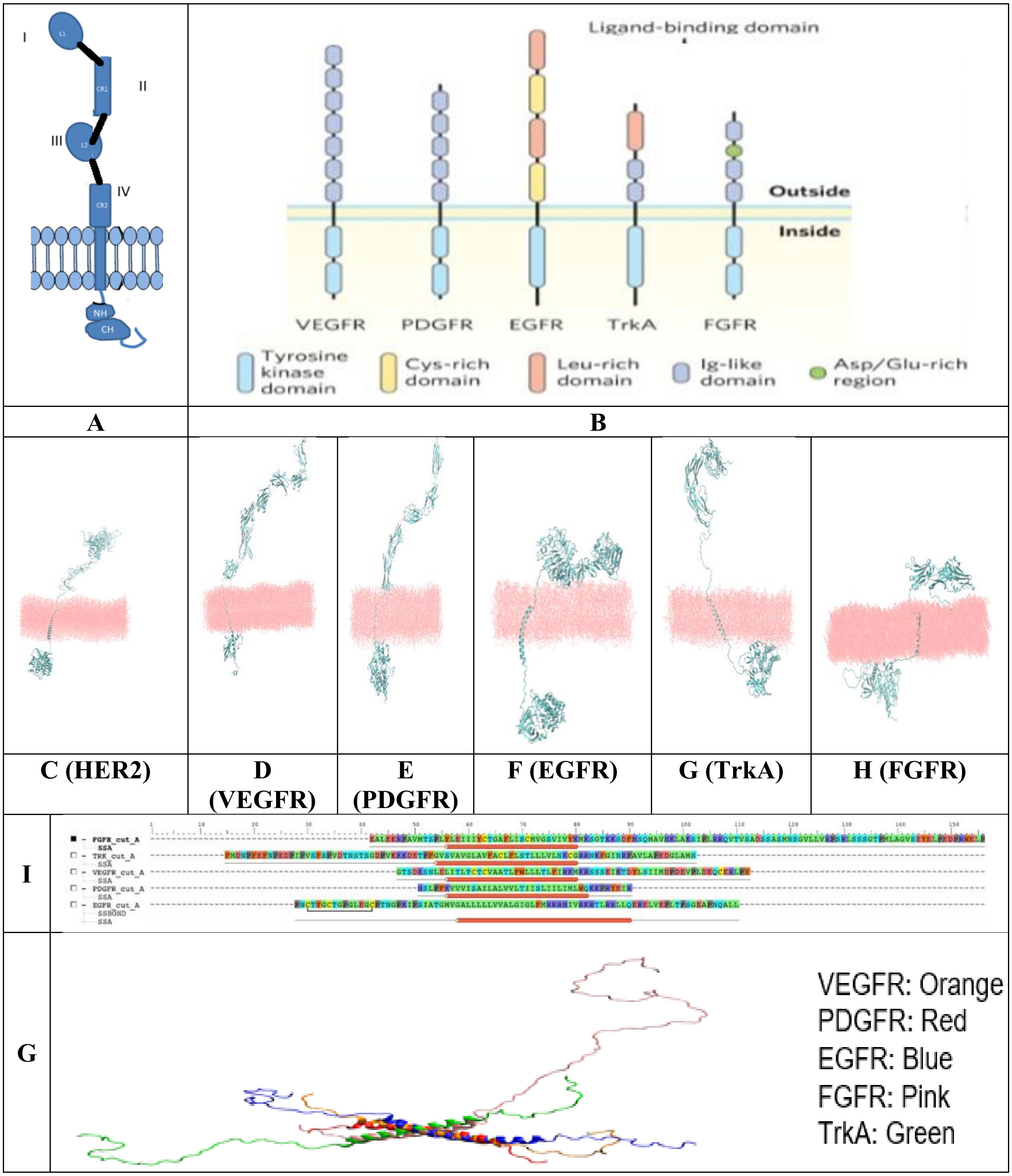

Figure S55. Diagram of receptor tyrosine kinases (**A-B**) and its high-resolution structure modified from Alpha Fold prediction (**C-H**). **A**: The domain of HER2 comprised of the extracellular domain, including two leucine-rich domains (I/L1 and III/L2) and two cysteine-rich domains (II/CR1, IV/CR2), the transmembrane domain, and intracellular domain, including the amino- (NH) and carboxyl-terminal lobes of the kinase domain and the carboxyl-terminal tail.[14]

B: The domain diagram of receptor tyrosine kinases: VEGFR, PDGFR, EGFR, TrkA, FGFR with tyrosine kinase domain in blue, cysteine-rich domain in yellow, leucine-rich domain in pink, Ig-like domain in purple, and Asp-Glu-rich region in green.[15] **C**: HER2 AlphaFold structure (P04626). **D**: VEGFR Alpha Fold structure (UniProt ID: P17948). **E**: PDGFR Alpha Fold structure (UniProt ID: P09616). **F**: EGFR Alpha Fold structure (UniProt ID: P00533). **G**: TrkA Alpha Fold structure (UniProt ID: P04629). **H**: FGFR Alpha Fold structure (UniProt ID: P11362). **I**: Multiple sequence alignment by the TM domain for these receptors. **G**: Multiple structure alignment by the TM domain for these receptors to show the common support string connecting extracellular AB domains and intracellular SI domains in the coupled pendulum model.

A

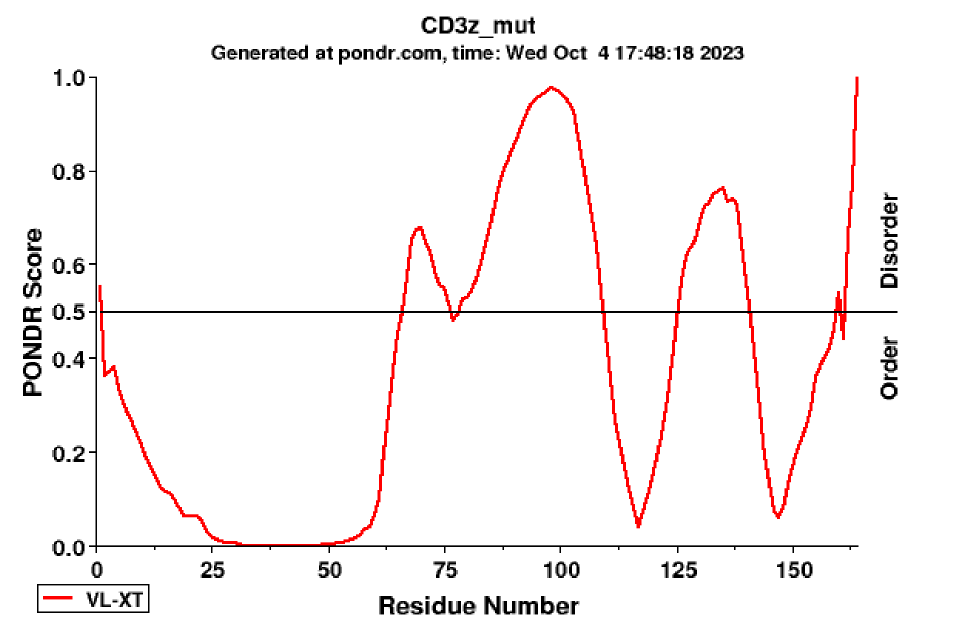

B

```
1        MKWKALFTAA ILQAQLPITE AQSFGLLDPK LCYLLDGILF IYGVILTALF
VLXT     D

51       LRVKFSRSAD APAYQQGQNQ LYNELNLGRR EEYDVLDAAA GADPEMGGAP
VLXT                       DDDD DDDDDD  DD DDDDDDDDDD DDDDDDDDDD

101      QAAANPQEGL YNELQKDKMA EAYSEIGMAG EAAAGAGHDG LYQGLSTATK
VLXT     DDDDDDDDD                  DDDDD DDDDDDDDDD

151      DTYDALHMQA LPPR
VLXT              D  DDD
```

Figure S56. Disordered regions of CD3ζ with alanine mutations of lysine and arginine of PBRs predicted by PONDR **A**: PONDR prediction diagram of ordered and disordered regions. **B**: PONDR sequence output of ordered and disordered regions with the PBRs in red.

Reference

1. Dunker AK, Brown CJ, Lawson JD, Iakoucheva LM, Obradovic Z. Intrinsic disorder and protein function. *Biochemistry* **41**, 6573-6582 (2002).

2. Lyman E*, et al.* A role for a specific cholesterol interaction in stabilizing the Apo configuration of the human A(2A) adenosine receptor. *Structure* **17**, 1660-1668 (2009).

3. Mark P, Nilsson L. Structure and Dynamics of the TIP3P, SPC, and SPC/E Water Models at 298 K. *The Journal of Physical Chemistry A* **105**, 9954-9960 (2001).

4. Khatami MH, Saika-Voivod I, Booth V. All-atom molecular dynamics simulations of lung surfactant protein B: Structural features of SP-B promote lipid reorganization. *Biochimica et biophysica acta* **1858**, 3082-3092 (2016).

5. Khatami MH, Bromberek M, Saika-Voivod I, Booth V. Molecular dynamics simulations of histidine-containing cod antimicrobial peptide paralogs in self-assembled bilayers. *Biochimica et biophysica acta* **1838**, 2778-2787 (2014).

6. Shaw DE*, et al.* Anton 2: Raising the Bar for Performance and Programmability in a Special-Purpose Molecular Dynamics Supercomputer. In: *SC14: International Conference for High Performance Computing, Networking, Storage and Analysis*) (2014).

7. Ikeguchi M. Partial Rigid-Body Dynamics in NPT, NPAT and NPgammaT Ensembles for Proteins and Membranes. *J Comput Chem* **25**, 529-541 (2004).

8. Bailey AG, Lowe CP. MILCH SHAKE: An Efficient Method for Constraint Dynamics Applied to Alkanes. *J Comput Chem* **30**, 2485-2493 (2009).

9. Shan Y, Klepeis JL, Eastwood MP, Dror RO, Shaw DE. Gaussian Split Ewald: A Fast Ewald Mesh Method for Molecular Simulation. *J Chem Phys* **122**, 54101-54101 (2005).

10. Stuart SJ, Zhou R, Berne BJ. Molecular Dynamics with Multiple Time Scales: The Selection of Efficient Reference System Propagators. *J Chem Phys* **105**, 1426-1436 (1996).

11. Humphrey W, Dalke A, Schulten K. VMD: Visual molecular dynamics. *J Mol Graphics* **14**, 33-38 (1996).

12. Wu W, Yan CS, Shi XS, Li LY, Liu WL, Xu CQ. Lipid in T-cell receptor transmembrane signaling. *Progress in Biophysics & Molecular Biology* **118**, 130-138 (2015).

13. Brenchley JM, Bosselut R. CD4 and CD8 Molecules: Molecular Biology, Expression, and Function. In: *Reference Module in Biomedical Sciences*). Elsevier (2014).

14. Tse C, Gauchez AS, Jacot W, Lamy PJ. HER2 shedding and serum HER2 extracellular domain: Biology and clinical utility in breast cancer (vol 38, pg 133, 2012). *Cancer Treatment Reviews* **39**, 831-831 (2013).

15. Nelson DL, Cox MM. *Lehninger Principles of Biochemistry*, 8th edn. W. H. Freeman (2021).
